# Real-World Effectiveness and Safety of Allergen Immunotherapy with a Combination of Glutaraldehyde-Polymerized Allergen Extracts of *Dermatophagoides* spp. and *Lepidoglyphus destructor* in Adults and Children

**DOI:** 10.3390/ijms27167397

**Published:** 2026-08-19

**Authors:** Francisco Javier Carballada González, Fernando García González, Belén Hinojosa Jara, Fernando Rodríguez Fernández, Miguel Añó García, Antonio Maraví San Martín, Luis Alfredo González Guzmán, Irene García Gutiérrez, Sofía Alonso Juaristi, María Jesús Giménez Revilla, Miguel Casanovas, Judith Díaz-García, Yara Ruiz García

**Affiliations:** 1 Lucus Augusti University Hospital, 27003 Lugo, Spain; 2 University Hospital Basurto, 48013 Bilbao, Spain; 3 Belén Hinojosa Medical Clinic, 21001 Huelva, Spain; 4 University Hospital Marqués de Valdecilla, 39008 Santander, Spain; 5 Alergocantabria, 39002 Santander, Spain; 6 Centro Médico Sanitas Sevilla, 41010 Sevilla, Spain; 7 Inmunotek S.L., Punto Mobi 5, Alcalá de Henares, 28805 Madrid, Spain

**Keywords:** allergen immunotherapy, *Dermatophagoides*, *Lepidoglyphus destructor*, real-world evidence

## Abstract

Mite-induced allergic respiratory diseases are a major global health concern, and evidence on allergen immunotherapy (AIT) combining allergens from different mite families remains limited, particularly for *Lepidoglyphus destructor*. This study evaluated the effectiveness and safety of subcutaneous allergen immunotherapy containing glutaraldehydempolymerized extracts of *Dermatophagoides pteronyssinus*, *Dermatophagoides farinae*, and *L. destructor* in mite-allergic patients in Spain. This retrospective multicenter study included 199 patients with allergic rhinoconjunctivitis, with or without asthma. Effectiveness outcomes included combined symptom and medication scores (CSMS), symptom scores, and medication use. Safety outcomes included adverse reactions. Mixed models for repeated measures showed significant improvements in rhinoconjunctivitis and asthma outcomes (all *p* < 0.001). Adjusted LS-mean rhinoconjunctivitis CSMS decreased by 50.7% at T1 and 76.1% at T4; symptom and medication scores decreased by 85.3% and 69.3%, respectively, at T4. Asthma CSMS decreased by 43.1% at T1 and 89.0% at T5, while symptom and medication scores decreased by 93.4% and 88.5%, respectively. Similar patterns were observed in children. No systemic reactions occurred. Twenty mild local reactions were recorded in 17 patients (8.5%), corresponding to an estimated rate of 0.32% across 6169 injections. This AIT was associated with sustained clinical improvement and a favorable safety profile in adults and children.

## 1. Introduction

Allergic respiratory diseases caused by mites represent a major global health concern, with house dust mites (HDMs) being among the most common perennial allergens associated with allergic rhinitis and asthma. Sensitization to HDMs is highly prevalent worldwide and frequently begins early in life, contributing to the development and persistence of respiratory allergic disease [1,2]. A study in 15 European countries estimated the prevalence of HDM sensitization to be 21.7% [3]. In addition, the occurrence of sensitization to this allergen is associated with an increase in respiratory pathologies in childhood [4].

In addition to *Dermatophagoides* spp., storage mites (SMs) such as *Lepidoglyphus destructor* have increasingly been recognized as relevant allergens. Their distribution varies according to climatic and environmental conditions, and sensitization to storage mites often occurs together with sensitization to HDMs. Recent studies suggest that the prevalence of sensitization to *L. destructor* has increased in recent years [5,6,7]. Importantly, recent studies suggest that allergy to *L. destructor* may be associated with more severe clinical manifestations and greater respiratory multimorbidity [2,6,8,9,10,11,12]. Furthermore, a recent study has shown that allergy to *L. destructor* is associated with a higher multimorbidity compared to patients allergic only to *Dermatophagoides* spp. [7], and monosensitization to these SMs is less frequent [12,13,14].

In Spain, few studies have reported the prevalence of sensitization to *L. destructor* [15,16,17,18,19], and only one clinical trial reported AIT evidence with this mite [20].

Although the efficacy and safety of AIT with *Dermatophagoides* spp. extracts have been widely demonstrated, evidence regarding AIT formulations combining HDMs with SMs remains limited [21,22,23,24,25]. This is particularly relevant as cross-reactivity between HDMs and storage mites is incomplete, suggesting that treatment targeting *Dermatophagoides* spp. alone may not fully address the clinical needs of polysensitized patients [26,27,28].

Therefore, this study aimed to assess the effectiveness and safety of subcutaneous immunotherapy with *Dermatophagoides* spp. and *L. destructor* in regions with high prevalence in Spain, such as Galicia, Cantabria, Andalucía, and País Vasco [29,30].

## 2. Results

### 2.1. Study Population

A total of 236 patients were identified from the clinical records. Following the application of predefined inclusion and exclusion criteria, 37 patients were excluded. The study population comprised a total of 199 patients, with a mean age of 25.7 years (95% CI: 23.8–27.6). Of these, 113 (56.8%) were male. The mean age was similar between patients with asthma (25.5 years) and those without asthma (26.1 years). Overall, 125 patients (62.8%) had asthma.

The χ2 tests provided statistically non-significant results, so no relationship can be established between age group and asthma diagnosis, nor between gender and asthma diagnosis.

The pediatric subgroup comprised 76 participants (38.2% of the total population), with a mean age of 12.3 years (95% CI: 11.5–13.1). In this subgroup, 57 patients (75.0%) were male and 19 (25.0%) female, and 50 children (65.8%) had asthma.

Again, there was no association between the gender and the diagnosis of asthma, as concluded from the χ2 tests.

### 2.2. Effectiveness

To evaluate longitudinal treatment effects while accounting for the correlation of repeated measurements within individuals, MMRM were applied to all quantitative outcomes. Overall, clinically relevant and statistically significant reductions in symptoms and medication use were observed during the treatment period.

**Figure 1:**
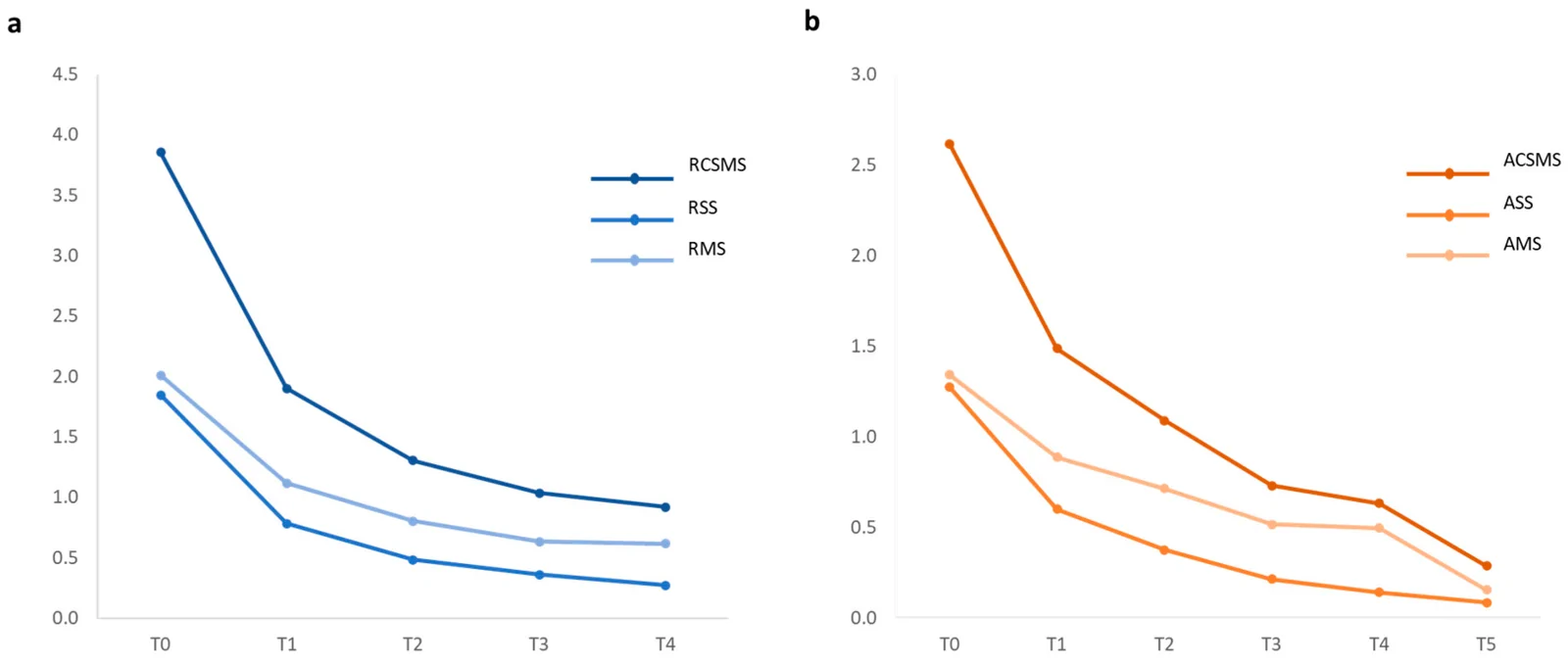
Longitudinal evolution of (**a**) rhinoconjunctivitis scores (RCSMS, RSS, RMS) and (**b**) asthma scores (ACSMS, ASS, AMS) estimated by the MMRM in the overall population. The numbers of evaluable patients at T0–T5 were 199, 199, 106, 61, 40, and 11, respectively, for rhinoconjunctivitis outcomes, and 125, 125, 70, 39, 28, and 7 for asthma outcomes.

For rhinoconjunctivitis outcomes, significant reductions were observed in RCSMS, RSS, and RMS. The effect of time was significant for RCSMS (F(4, 249.040) = 251.257, *p* < 0.001), RSS (F(4, 378.659) = 329.483, *p* < 0.001), and RMS (F(4, 211.579) = 95.555, *p* < 0.001). RCSMS adjusted LS-means decreased from 3.854 at baseline to 1.900 at T1, 1.303 at T2, 1.036 at T3, and 0.920 at T4, corresponding to reductions of 50.7%, 66.2%, 73.1%, and 76.1%, respectively. Comparisons between baseline and each follow-up time point from T1 to T4 were statistically significant after Sidak adjustment (all *p* < 0.001). RCSMS also decreased significantly between T1 and each subsequent time point, whereas differences among T2, T3, and T4 were not statistically significant. The adjusted marginal mean at T5 was not estimable and was therefore not included in the model-based interpretation. Similar trends were observed for RSS, with reductions from 57.5% at T1 to 85.3% at T4, and for RMS, for which medication use decreased by 44.5% at T1 and by approximately 69.3% at T4 (Figure 1a).

Among patients with asthma, significant improvements were also observed in asthma-related outcomes. The time effect was significant for ACSMS (F(5, 96.189) = 81.556, *p* < 0.001), ASS (F(5, 174.451) = 107.649, *p* < 0.001), and AMS (F(5, 71.749) = 35.608, *p* < 0.001). The estimated LS-mean differences showed a progressive reduction from baseline across visits. For ACSMS, reductions reached 43.1% at T1 and 89.0% at T5. ASS decreased from 52.8% at T1 to 93.4% at T5, while AMS declined from 34.0% at T1 to 88.5% at T5 (Figure 1b).

In the MMRM analysis, a significant effect of time was observed for both RCSMS (F(4, 249.040) = 251.257, *p* < 0.001) and ACSMS (F(5, 96.189) = 81.556, *p* < 0.001), indicating progressive changes during follow-up. For RCSMS, the interaction between time and age group was significant (F(5, 170.520) = 3.896, *p* = 0.002), whereas the main effect of age group did not reach statistical significance (*p* = 0.053). In contrast, for ACSMS, a significant effect of age group was detected (F(1, 157.268) = 6.701, *p* = 0.011), but the interaction with time was not significant (F(5, 171.719) = 0.486, *p* = 0.786). Overall, these findings suggest differences in overall score levels between pediatric and adult patients, while the temporal evolution during follow-up was broadly similar across age groups.

For RCSMS, the significant time-by-age-group interaction suggested that the trajectory of change differed statistically between pediatric and adult patients; however, the absence of a significant main age-group effect indicated no consistent overall difference in adjusted score levels across visits. For ACSMS, the significant age-group effect indicated different overall adjusted score levels, whereas the non-significant time-by-age-group interaction suggested broadly parallel changes during follow-up. These exploratory findings do not demonstrate a superior treatment response in either age group.

To assess robustness independent of distributional assumptions, complementary non-parametric analyses were performed using the Wilcoxon signed-rank test for T0–T1 comparisons and the Friedman test for repeated measures across available follow-up.

RCSMS showed a marked and sustained improvement over time, with progressive reductions relative to baseline. The median score decreased from 3.7 at T0 to 1.7 after one year of immunotherapy, corresponding to a 54% reduction. Improvements continued throughout follow-up, reaching a median of 0.2 at T5, representing an overall reduction of approximately 95% (Table 1).

**Figure 2:**
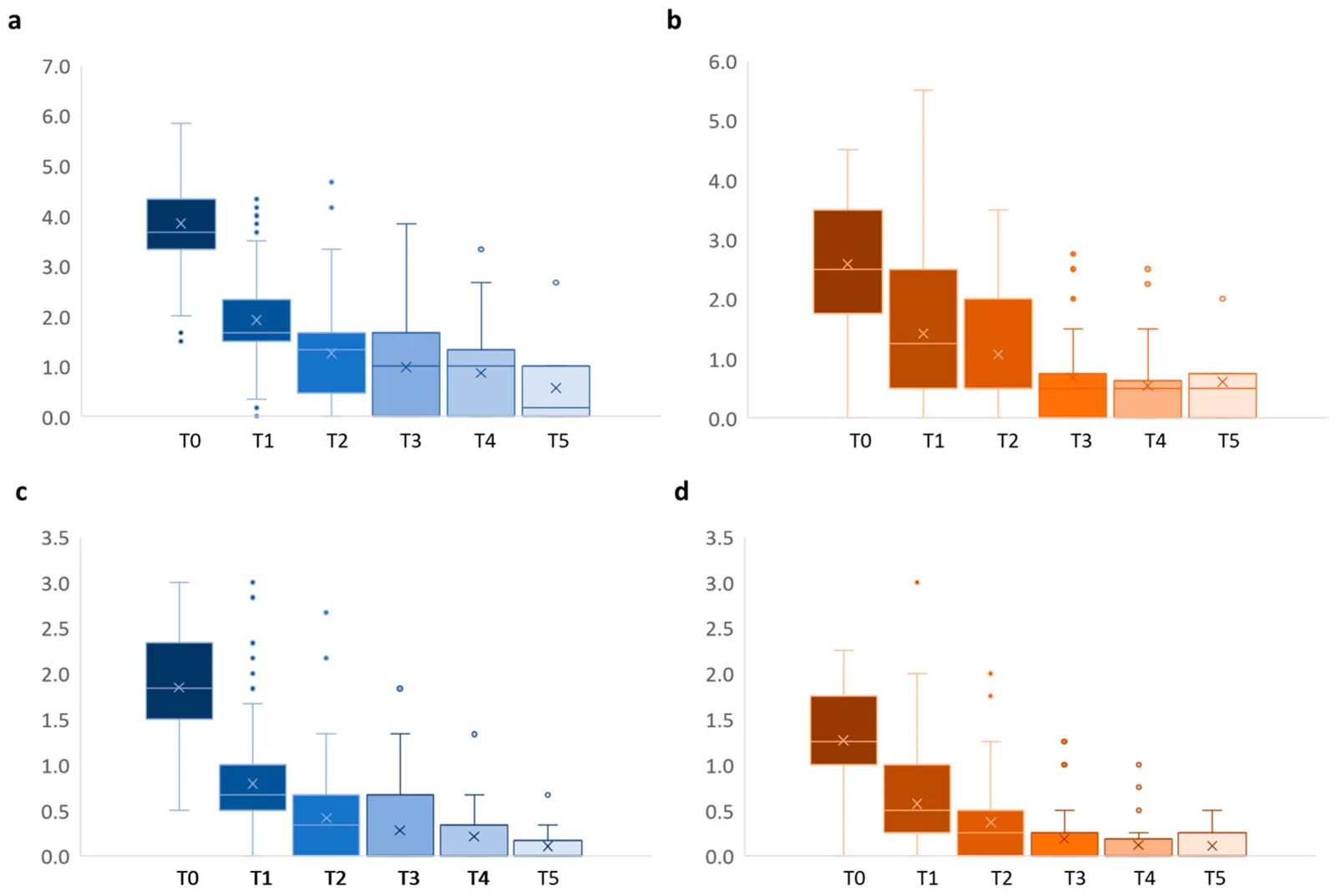
Box plot of the distribution of CSMS and SS of (**a**,**c**) Rhinoconjunctivitis and (**b**,**d**) Asthma respectively, across the study period. Dots represent individual outlying observations beyond the whiskers.

The interquartile ranges also shifted toward lower values, with the lower quartile reaching 0 from T3 onward, indicating that a substantial proportion of patients achieved minimal or no symptoms with little or no need for rescue medication. These findings support a sustained and clinically meaningful reduction in rhinoconjunctivitis disease burden during long-term immunotherapy (Figure 2a).

ACSMS also showed a sustained improvement over time, with progressive reductions in median scores. At T1, the median ACSMS was 1.3, indicating residual symptoms and medication use and a 48% reduction compared to T0. By T2, it decreased to 0.5, and this improvement was maintained throughout follow-up, resulting in a sustained reduction of about 80% (Table 1).

Interquartile ranges progressively shifted toward lower values, with the lower quartile reaching 0 from T3 onward, indicating that an increasing proportion of patients achieved complete asthma control without symptoms or rescue medication. Despite fewer evaluable patients at later visits, the persistence of low median CSMS values supports a durable improvement in asthma control during allergen immunotherapy (Figure 2b).

Effect size analysis showed a large and sustained treatment effect for rhinoconjunctivitis, with RCSMS remaining in the large range across follow-up. For asthma, ACSMS effect sizes were large during most of the follow-up and decreased to the moderate range at the latest time point, likely due to the smaller sample size, while still indicating clinically meaningful improvement in asthma control (Table 1).

In line with the combined score, RSS showed a progressive reduction during follow-up compared with baseline (T0). Median RSS decreased by 63.4% at T1 and 80.2% at T2, reaching 0 from T3 onward and remaining at this level through T5. The reduction between T0 and T1 was significant, and longitudinal analysis confirmed significant differences between T0 and all subsequent time points. Effect sizes were consistently large (Figure 2c, Table 2).

Similarly, ASS showed a marked reduction from T1 onward, with decreases of 60.0% at T1 and 80.0% at T2, reaching 100% reduction from T3 through the end of follow-up. Improvements between T0 and T1 were significant, and longitudinal analyses confirmed differences across follow-up visits. Effect sizes remained large throughout treatment, indicating clinically meaningful improvement in asthma symptoms. (Figure 2d, Table 2).

**Figure 3:**
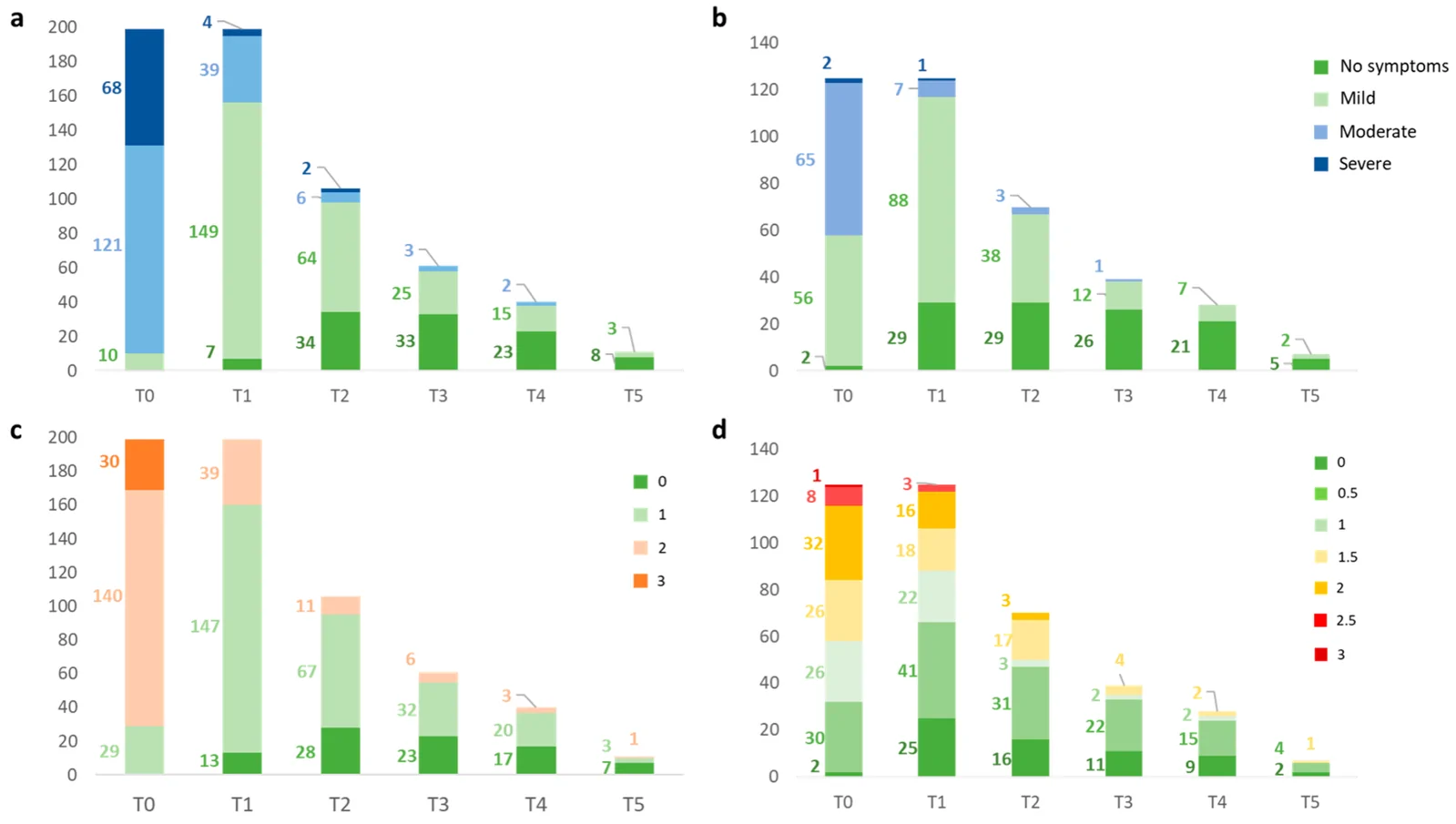
Distribution of rhinoconjunctivitis and asthma symptom severity ((**a**) RSS; (**b**) ASS) and medication use ((**c**) RMS; (**d**) AMS) categories from baseline to five years of allergen immunotherapy. Stacked bars show the distribution of patients across severity categories at each time point (T0–T5), with numbers within each coloured segment indicating the corresponding patient count.

To further characterize treatment effectiveness, the distribution of symptom and medication severity categories was analyzed across the study period to provide a detailed characterization of changes in disease burden for both rhinoconjunctivitis and asthma (Figure 3).

The distribution of symptom severity categories showed a clear improvement over time for both rhinoconjunctivitis and asthma. At baseline, most patients with rhinoconjunctivitis had moderate (60.8%) or severe symptoms (34.2%), with no symptom-free patients. After one year, most shifted to mild symptoms (74.9%), and, from T2 onward, an increasing proportion became symptom-free, reaching 54.1% at T3 and 72.7% at T5, with severe symptoms disappearing after T2 (Figure 3a). A similar pattern was observed for asthma, where symptom-free patients increased from 1.6% at baseline to 23.2% at T1 and to more than two-thirds from T3 onward, with no moderate or severe cases at T5 (Figure 3b).

Medication use followed a similar trend. At baseline, more than 85% of patients with rhinoconjunctivitis required moderate to high medication levels (score 2–3). After treatment initiation, medication requirements decreased rapidly, with most patients shifting to RMS score 1 at T1 and an increasing proportion requiring no medication from T2 onward, reaching 63.6% at T5 (Figure 3c). Asthma medication use also progressively declined, with 89.7% of patients receiving low-dose or no treatment and no patients requiring higher-step therapy from T3 onward (Figure 3d).

### 2.3. Effectiveness in Pediatric Patients

A sub-analysis in pediatric patients was performed separately. MMRM were applied to evaluate longitudinal changes in symptoms and medication scores as well. A significant effect of time was observed for all outcomes, indicating a progressive improvement during treatment.

**Figure 4:**
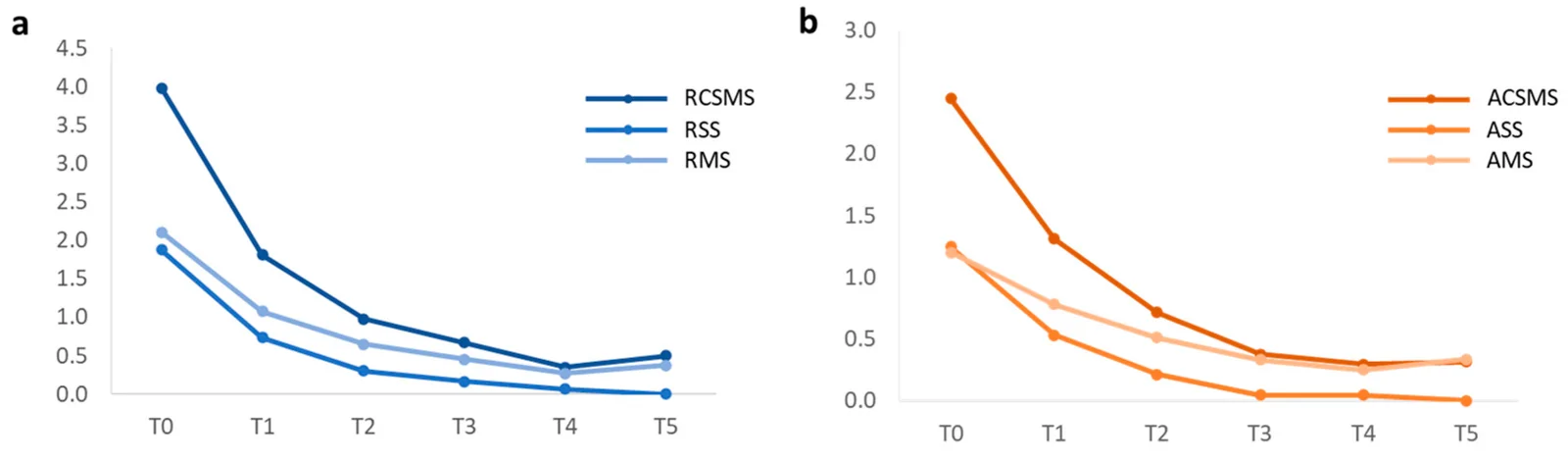
Longitudinal evolution of (**a**) rhinoconjunctivitis scores (RCSMS, RSS, RMS) and (**b**) asthma scores (ACSMS, ASS, AMS) estimated by the MMRM in the pediatric population.

For rhinoconjunctivitis outcomes, significant reductions were observed in the RCSMS, RSS, and RMS. The time effect was significant for RCSMS (F(5, 33.911) = 152.855, *p* < 0.001), RSS (F(5, 109.095) = 149.848, *p* < 0.001), and RMS (F(5, 33.574) = 82.797, *p* < 0.001). Compared with baseline, the estimated LS-mean differences increased progressively across follow-up visits. For RCSMS, reductions reached 54.5% at T1 and increased to 91.3% at T4, remaining high at T5 (87.5%). A similar pattern was observed for RSS, with reductions from 60.9% at T1 to 96.4% at T4 and complete symptom resolution at T5. Medication use also decreased substantially, with RMS reductions ranging from 48.8% at T1 to 87.3% at T4 and 82.3% at T5 (Figure 4a).

Among pediatric patients with asthma, significant improvements were also observed in asthma-related outcomes. The time effect was significant for ACSMS (F(5, 73.737) = 48.833, *p* < 0.001), ASS (F(5, 72.774) = 58.206, *p* < 0.001), and AMS (F(5, 72.364) = 17.626, *p* < 0.001). ACSMS showed progressive reductions from 46.4% at T1 to 88.0% at T4 and 87.2% at T5. ASS declined markedly during follow-up, with reductions from 57.4% at T1 to 96.5% at T3–T4 and complete resolution at T5. Similarly, AMS decreased over time, from 35.0% at T1 to 79.2% at T4 and 72.1% at T5 (Figure 4b).

Overall, the MMRM analysis in pediatric patients confirmed a marked and sustained reduction in both symptoms and medication use throughout the treatment period.

As previously done, complementary non-parametric analyses were conducted.

**Figure 5:**
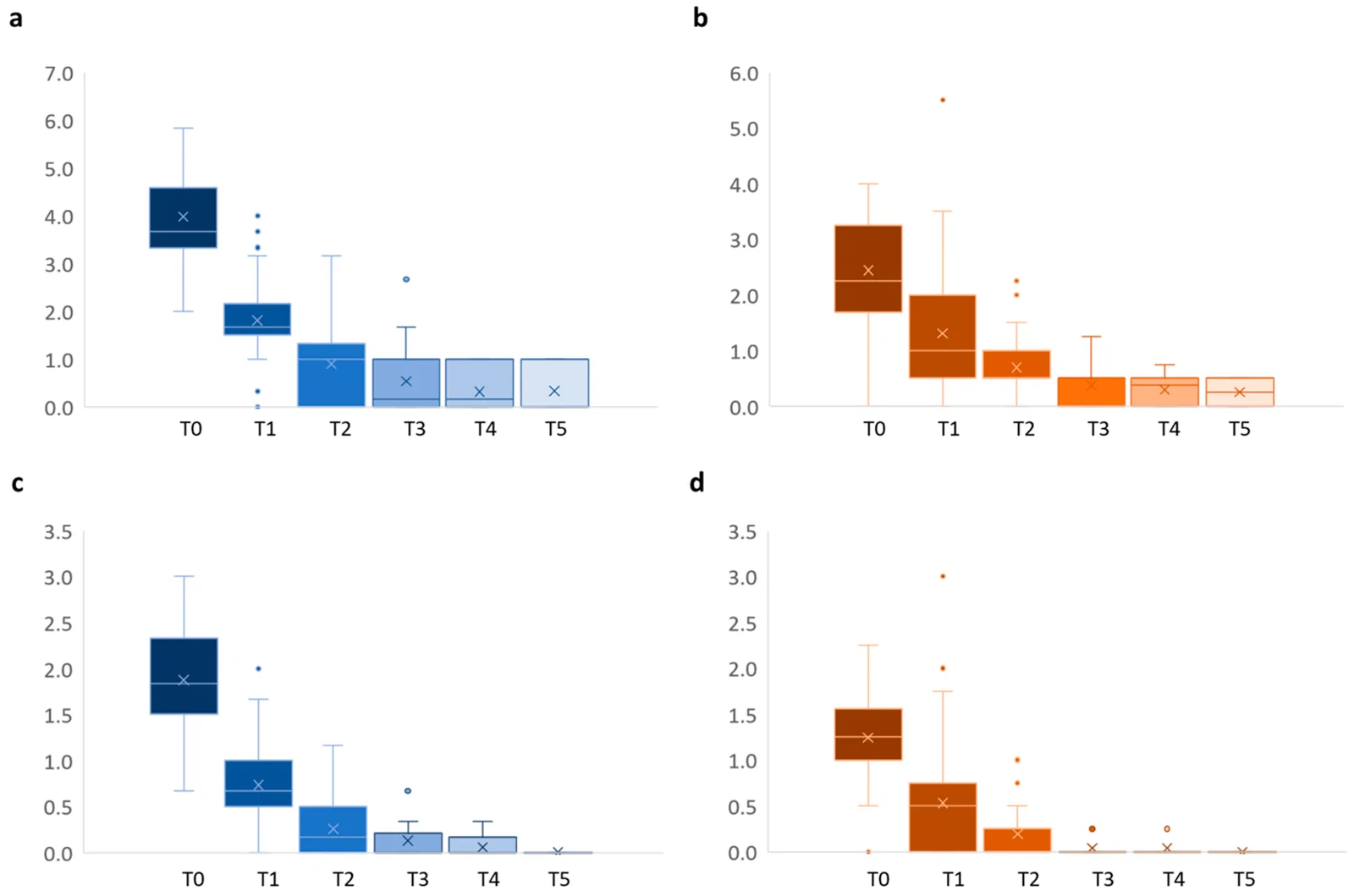
Box plots showing the distribution of CSMS and SS in pediatric patients with (**a**,**c**) rhinoconjunctivitis and (**b**,**d**) asthma, respectively, across the study period. Dots represent individual outlying observations beyond the whiskers.

At baseline, the median RCSMS was 3.7 (IQR: 3.3–4.4). A significant reduction was observed at T1 (median 1.7; IQR: 1.5–2.2), corresponding to a 54.5% decrease (*p* < 0.0001; large effect size). The median continued to decline to 1.0 at T2 (72.8% reduction) and 0.2 at T3–T4 (95.4% reduction), reaching 0.0 at T5 (IQR: 0–0.5), corresponding to a 100% reduction from baseline. Longitudinal analyses confirmed significant differences across follow-up visits (*p* < 0.0001), with consistently large effect sizes (Figure 5a, Table 2).

The median ACSMS was 2.5 (IQR: 1.8–3.4) at baseline and decreased significantly to 1.3 at T1 (IQR: 0.5–2.3), representing a 48.0% reduction (*p* < 0.0001; large effect size). From T2 onward, the median declined to 0.5 and remained stable through T5, corresponding to a sustained 80.0% reduction. Longitudinal analyses showed significant differences up to T4 (*p* < 0.0001), although significance was not reached at T5 (*p* = 0.105), likely due to the smaller sample size (Figure 5b, Table 2).

RSS showed a marked and sustained reduction during follow-up compared with baseline. A significant decrease was observed at T1 (63.4% reduction), which increased to 89.8% at T2. From T3 onward, the median RSS reached zero, indicating a 100% reduction maintained through T5. Effect sizes were consistently large, indicating a strong clinical improvement (Figure 5c, Table 2).

Similarly, ASS decreased substantially from the first follow-up visit. The median ASS was reduced by 60.0% at T1 and reached a 100% reduction at T2, which was maintained thereafter. Effect sizes remained large across follow-up, indicating clinically meaningful improvement (Figure 5d, Table 2).

**Figure 6:**
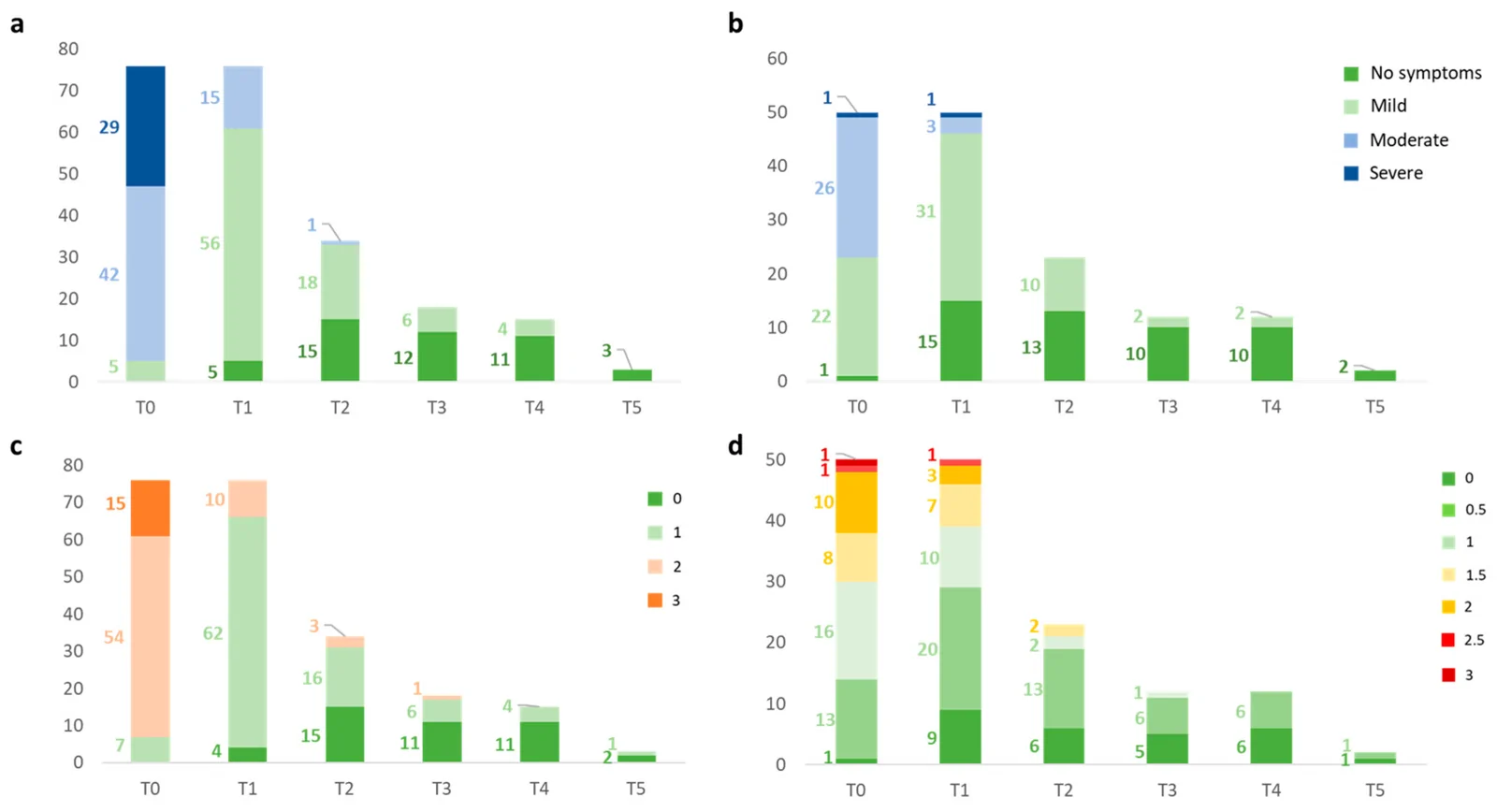
Distribution of rhinoconjunctivitis and asthma symptom severity ((**a**) RSS; (**b**) ASS) and medication use ((**c**) RMS; (**d**) AMS) categories from baseline to five years of allergen immunotherapy in pediatric patients. Stacked bars show the distribution of patients across severity categories at each time point (T0–T5), with numbers within each coloured segment indicating the corresponding patient count.

The distribution of symptom and medication severity categories among pediatric patients was analyzed across the study period to provide a detailed assessment of changes in disease burden (Figure 6).

At baseline, most children presented a moderate to severe symptom burden. For rhinoconjunctivitis, 55.3% of patients were classified as having moderate symptoms and 38.2% as severe, with no patients reporting absence of symptoms (Figure 6a). Similarly, asthma symptoms were predominantly moderate (52.0%), with only 2.0% of patients being symptom-free (Figure 6b). Following initiation of allergen immunotherapy, a rapid and pronounced shift toward milder symptom categories was observed. At T1, 73.7% of children with rhinoconjunctivitis were classified as having mild symptoms, and no severe symptoms were reported. At T2, 44.1% were symptom-free, and this proportion exceeded 70% at T4. Results at T5 were not interpreted because only three children with rhinoconjunctivitis and two children with asthma had evaluable data.

A similar trend was observed for asthma symptoms (Figure 6b). After one year of treatment, the proportion of children with no asthma symptoms increased to 30.0%, while moderate symptoms decreased to 6.0%. From T2 onward, no patients were classified as having moderate or severe asthma symptoms, and more than 80% of patients were symptom-free from T3 onward.

Changes in medication use paralleled the improvements observed in symptom severity. At baseline, most pediatric patients required moderate to high levels of medication for rhinoconjunctivitis, with over 70% classified as RMS score 2 and nearly 20% as score 3. After treatment initiation, medication requirements decreased substantially, with increasing proportions of patients requiring no medication from T2 onward. By T4, more than 70% of patients were medication-free (Figure 6c).

Asthma medication use showed a comparable pattern, with a progressive shift toward low-dose or no medication categories over time, and no patients requiring higher-step treatments at later follow-up visits (Figure 6d).

### 2.4. Safety

Regarding the safety of AIT with *Dermatophagoides*/*L. destructor*, no systemic reactions were recorded. A total of 20 mild local adverse reactions occurred in 17 of the 199 patients, corresponding to 8.5% of the study population. Seven reactions occurred in seven pediatric patients, whereas 13 reactions occurred in 10 adult patients. Thus, three adult patients experienced more than one reaction. All of them were reported during the first year of treatment. An estimated total of 6169 injections were administered during the study. This total accounts for the rush initiation schedule, consisting of two injections on the first treatment day, followed by monthly maintenance injections, and is based on a mean treatment duration of 2.5 years. Considering the number of injections was 6169, the proportion of adverse reactions was 0.32% in the study. The most frequent reactions consisted of mild local inflammatory reactions (erythema, edema, or inflammation at the injection site), with a short duration of approximately 0.5 h in the majority of cases. These were mainly immediate, although a small number of delayed local reactions (>15 cm) were also reported. All reactions were classified as mild, with no severe adverse events and no criteria of seriousness fulfilled. A total of 14 patients with these adverse reactions did not require pharmacological treatment, and when treatment was needed, it consisted of simple measures such as local cold application or antihistamines; in 30% of local reactions, pharmacological treatment was administered without modification of immunotherapy. Importantly, no discontinuation of treatment was required, and all patients fully recovered from the adverse reactions. Overall, the safety profile was favorable across age groups and genders, with complete resolution of all adverse reactions.

## 3. Discussion

While the prevalence and clinical impact of allergic respiratory diseases caused by HDMs have been extensively described worldwide, the role of sensitization to SMs, particularly *L. destructor*, has received comparatively less attention. Recent evidence indicates that this mite species is no longer confined to rural or occupational settings but is increasingly prevalent in both urban and domestic environments, contributing to more severe and complex clinical phenotypes of allergic rhinitis and asthma. In this context, the present study provides novel real-world evidence on the effectiveness and safety of AIT combining *Dermatophagoides* spp. and *L. destructor* in a region of high mite exposure.

These findings contribute to the limited real-world evidence on the effectiveness and safety of subcutaneous immunotherapy containing polymerized extracts of *Dermatophagoides* spp. and *L. destructor* in both adult and pediatric patients [30]. The inclusion of *L. destructor* is of particular clinical relevance, as previous studies have shown limited cross-reactivity between HDMs and SMs, suggesting that immunotherapy targeting *Dermatophagoides* spp. alone may be insufficient in polysensitized patients [7]. The present findings therefore support a personalized approach to AIT that reflects regional exposure patterns and individual sensitization profiles.

Regarding effectiveness, clinically meaningful and sustained reductions in disease burden were observed by the MMRM analyses and confirmed by the non-parametric tests. These longitudinal models showed a progressive decline in both symptoms and medication use over time for rhinoconjunctivitis and asthma outcomes, with improvements already evident after the first year of treatment and maintained throughout follow-up. In rhinoconjunctivitis, RCSMS showed a marked reduction together with parallel decreases in RSS and RMS, indicating a consistent improvement in clinical control. Similarly, among patients with asthma, symptom and medication scores, as well as their combined score, progressively declined during treatment.

The magnitude of the observed effects was further supported by consistently large effect sizes across most outcomes and time points. The early onset of clinical benefit, combined with its long-term persistence, reinforces the disease-modifying potential of allergen immunotherapy in mite-induced respiratory allergy. Nevertheless, given the retrospective nature of the study, these findings should be interpreted with caution.

The pediatric subgroup analysis is of particular relevance. Children exhibited early and sustained improvements comparable to those observed in adults, with large effect sizes maintained throughout follow-up. Given the established association between early mite sensitization, impaired lung function, and long-term asthma severity [4], these findings support the initiation of AIT at younger ages as a strategy not only for symptom control but also for modifying disease progression.

Age-related findings should be interpreted cautiously. The observed statistical differences may reflect differences in baseline score distributions, clinical characteristics, or the composition of the progressively smaller follow-up subsets rather than a distinct biological response to AIT. The study was not designed or powered to establish mechanistic differences between pediatric and adult patients.

The number of evaluable patients progressively decreased at later visits, particularly beyond three years. At T5, data were available for only 11 patients with rhinoconjunctivitis and 7 patients with asthma. Consequently, estimates at the latest time points have limited precision and may not be representative of the original cohort. Long-term findings should therefore be interpreted as exploratory observations from selected subsets of patients with longer available follow-up rather than as estimates generalizable to the full study population.

The safety profile observed in this study was consistent with previous reports on subcutaneous mite immunotherapy and aligns with the improved tolerability described for polymerized allergen extracts compared with conventional native formulations [21,22,23] and aligns with recent advances in modified allergen extracts [31,32]. However, direct comparative evidence of superior efficacy over conventional native extracts remains limited. No systemic reactions were recorded, and 20 mild local reactions occurred in 17 patients, representing 8.5% of the study population and an estimated rate of 0.32% per injection. No treatment discontinuations were required. These findings are in line with previous studies evaluating polymerized mite extracts and support the use of cluster schedules even in mixed mite formulations, including pediatric patients [33].

Combination AIT may provide broader allergen coverage in patients sensitized to both house dust and storage mites, especially when cross-reactivity is incomplete. However, it may increase allergen exposure, complicate attribution of adverse reactions, and include allergens of uncertain clinical relevance. In this context, the use of polymerized allergen extracts may facilitate combined AIT by reducing allergenic activity while allowing clinically relevant allergens from different mite sources to be administered within the same formulation. Therefore, combination formulations should be individualized and supported by clinical history, regional exposure, and sensitization findings.

The geographical origin of the study population should also be considered when interpreting the findings. Patients were recruited from regions including Galicia, Cantabria, Andalusia, and the Basque Country, in which clinically relevant sensitization to house dust and storage mites has been reported. This regional context may partly explain the high relevance of simultaneous sensitization to *Dermatophagoides* spp. and *L. destructor* in the study population and supports the selection of AIT according to local exposure patterns. However, these findings may not be directly generalizable to geographical areas in which exposure to *L. destructor* is less frequent.

This study has limitations inherent to its retrospective, observational design, including the absence of a control group and the progressive reduction in sample size at later follow-up visits. Another limitation is the requirement for at least one year of evaluable follow-up. Patients without an available one-year assessment within the predefined retrospective study period or with unavailable one-year follow-up data were excluded. Although these exclusions were not based on treatment discontinuation due to lack of effectiveness or adverse events, some degree of selection bias cannot be excluded. In addition, although the CSMS was based on the EAACI recommendation, it was retrospectively reconstructed from routine medical records rather than calculated from prospective daily patient diaries. Consequently, it may be affected by documentation bias and patient recall, particularly at baseline.

However, the within-patient longitudinal design, the use of standardized outcome measures, and the consistency of results across multiple endpoints and age groups strengthen the validity of the findings. Moreover, real-world evidence provides valuable complementary information to randomized clinical trials by reflecting routine clinical practice and heterogeneous patient populations.

In addition, AIT initiation was based on the treating physician’s clinical judgment rather than on randomized allocation or a uniform, study-mandated treatment algorithm. Therefore, confounding by indication and residual confounding related to base-line disease severity cannot be excluded. The absence of an untreated comparator group prevents determination of the direction or magnitude of this potential bias.

## 4. Materials and Methods

### 4.1. Study Design

This is a multicenter, observational, retrospective, and longitudinal study designed to evaluate the effectiveness and safety of AIT with *Dermatophagoides* and *L. destructor* in routine clinical practice. The study had two primary objectives: (1) to evaluate its effectiveness, assessed through clinical outcomes and changes in symptom control and medication use, and (2) to assess the safety of immunotherapy, based on the incidence, type, and severity of adverse reactions. In case of systemic adverse reactions, the grading system published by the WAO in 2024 was applied [34]. Data were collected retrospectively from clinical records and analyzed descriptively and inferentially according to the predefined objectives.

### 4.2. Study Population

Participants aged 5 to 65 years with clinically relevant allergic rhinoconjunctivitis with or without asthma were included. Clinical relevance was established by the treating allergist based on the concordance between respiratory symptoms, allergen exposure, and documented sensitization. Sensitization was confirmed by a positive skin prick test (≥3 mm) and/or positive molecular sensitization determined by specific IgE (sIgE ≥ 0.75 kU/L) measured using ImmunoCAP™ (Thermo Fisher Scientific, Uppsala, Sweden) and/or Allergy Xplorer (Macro Array Diagnostics GmbH, Vienna, Austria). Patients were sensitized to both mite species, with at least one molecular allergen identified for each species, and had received at least one year of treatment. Patients were excluded if they had previously received AIT containing *Dermatophagoides* spp. or *L. destructor*, if their first-year assessment fell outside the predefined retrospective study period, or if one-year follow-up data were unavailable.

Patients were recruited from allergy centers located in regions of Spain in which exposure and sensitization to house dust and storage mites are clinically relevant.

The decision to initiate AIT had been made before and independently of study in-clusion as part of routine clinical practice. No study-specific treatment allocation algo-rithm was applied. Treating allergists considered the clinical relevance of the respiratory symptoms together with documented sensitization to both mite sources; however, the final decision remained at the physician’s discretion.

### 4.3. Immunotherapy Preparation

Patients received subcutaneous immunotherapy with a combination of polymerized glutaraldehyde allergen extracts of *Dermatophagoides* (*D. pteronyssinus* and *D. farinae*) and *L. destructor*, produced by Inmunotek S.L. (Alcalá de Henares, Madrid, Spain), and supplied under the trade names of Clustoid^®^ (Inmunotek S.L., Alcalá de Henares, Madrid, Spain) and Clustek^®^/Alxoid^®^ (Inmunotek S.L., Alcalá de Henares, Madrid, Spain) in other countries. For the purposes of this study, these trade names are considered interchangeable, as they refer to the same medicinal product.

Dosing schedules, dose adjustments, and maintenance intervals were determined according to routine clinical practice and the treating physician’s judgment, with no protocol-mandated modifications. A rush up-dosing schedule was employed, consisting of an initial administration of 0.2 mL in one arm followed 30 min later by 0.3 mL in the contralateral arm, after which maintenance doses of 0.5 mL were administered every 30 days.

### 4.4. Study Endpoints

To assess effectiveness, the evolution of symptoms as well as medication prescriptions has been considered through the Combined Symptom and Medication Score (CSMS) for both rhinoconjunctivitis and asthma (RCSMS and ACSMS, respectively), which is calculated as the sum of the Symptom Score (SS) and Medication Score (MS).

In the case of rhinoconjunctivitis, the symptoms considered for the study were itchy nose, sneezing, runny nose, blocked nose, itchy/red eyes, and watery eyes. In the case of patients with asthma, these 4 symptoms were considered: cough, dyspnea, chest tightness, and wheezing. Rhinoconjunctivitis and asthma symptom scores (RSS and ASS, respectively) were calculated according to EAACI recommendations using standardized symptom scales. Each symptom was graded on a 0–3 scale according to severity [35].

In the case of medication, the same criteria from 0 to 3 were also used according to the need for medication for rhinoconjunctivitis through the Rhinoconjunctivitis Medication Score (RMS): no medication, oral or topical antihistamines (eyes and/or nose), intranasal corticosteroids with or without antihistamines, and oral corticosteroids with or without antihistamines. For asthma, the criteria to be followed to establish the Asthma Medication Score (AMS) were based on the therapeutic steps for the maintenance treatment of asthma published in GEMA (Spanish Guidelines for Asthma Management) [36]. The scoring system applied in this study has been previously described [37].

To assess effectiveness, the quantitative variables of SS and MS were considered separately and in combination for rhinoconjunctivitis and asthma. These allowed for assessing not only the clinical impact of the treatment on patients when analyzing rhinoconjunctivitis and asthma symptoms but also the effect on the use of medical resources, such as medications for these two allergic diseases. Treatment effectiveness was evaluated at baseline (T0, prior to initiation of AIT) and subsequently at annual intervals, with a minimum follow-up of one year (T1) and up to a maximum of five years (T5) for both rhinoconjunctivitis and asthma. T0 was defined as the last available clinical assessment before the initiation of AIT. Follow-up assessments were assigned to T1–T5 according to the routine clinical visit closest to each treatment anniversary, using a predefined window of ±6 months around 12, 24, 36, 48, and 60 months, respectively.

Safety was assessed by the systematic recording and characterization of adverse reactions (ARs) throughout the observation period. ARs were classified as local reactions when occurring at the injection site and were further categorized as immediate (≤30 min) or delayed (>30 min). They were considered clinically relevant if they exceeded 5 cm in diameter and occurred within 30 min of administration, or exceeded 10 cm in diameter when they occurred later. ARs were categorized as systemic AR when occurring away from the injection site, typically within minutes and rarely beyond 30 min after administration, and were graded according to the World Allergy Organization (WAO) five-grade severity classification [34].

### 4.5. Statistical Analysis

To show the population sociodemographic characteristics, a descriptive analysis was performed. The age was shown with the mean, standard deviation (SD), confidence interval (CI), median, and interquartile 1 and 3 (Q1, Q3). To assess the size of the pediatric population, the participants were divided into two groups: from 5 to 17 years of age (pediatric population) and from 18 to 65 years of age (adults). Regarding the qualitative variables (gender, age group, and asthma diagnosis), the absolute frequencies of these groups were shown for both the rhinoconjunctivitis and asthma subgroups.

Contingency tables were performed using the χ2 test to determine whether there was a relationship between age range and gender and the diagnosis of asthma.

For the evaluation of safety, an estimation of the proportion of adverse events and a description of each reaction reported were given.

Quantitative variables, including the SS and MS, were collected at multiple time points between diagnosis and throughout treatment. These longitudinal measurements reflected the temporal evolution of each patient, and therefore statistical analyses were based on comparisons of related (paired) variables over time to evaluate effectiveness.

The administration of AIT to modulate the evolution of allergic diseases is more challenging in the pediatric population, as the immune system remains immature. The effectiveness and safety might be reduced compared to the results from the adult cohort. Therefore, it was considered to perform a sub-analysis on participants aged 5 to 17 years.

All patients had available data at baseline (T0) and at the first-year follow-up (T1). The number of observations decreased at later timepoints because treatment duration varied among patients within the predefined retrospective study period. Therefore, analyses at T2–T5 reflect progressively smaller subsets of patients with longer available follow-up and should not be interpreted as representing the entire study cohort.

The distribution of quantitative variables was first assessed for normality through the Kolmogorov–Smirnov test or the Shapiro–Wilk test.

Based on the distributional characteristics, appropriate statistical tests were selected to evaluate the null hypothesis. As the analyses involved repeated measurements over time, Mixed Models for Repeated Measures (MMRM) were used as the primary analysis to evaluate longitudinal changes while accounting for within-subject correlations across repeated measurements [38,39].

For rhinoconjunctivitis outcomes (RCSMS, RSS, and RMS), fixed effects included time (categorical), gender, age (continuous), and asthma status (yes/no). Analyses of asthma outcomes (ACSMS, ASS, and AMS) were restricted to patients with asthma and included time, sex, and age as fixed effects. Within-subject correlations were modeled using a first-order autoregressive covariance structure (AR [1]), and parameters were estimated by restricted maximum likelihood (REML). Least-squares means (LS-means) with standard errors (SE) and 95% CI were obtained for each timepoint, and pairwise comparisons versus baseline were adjusted using the Sidak method. Potential effect modification was explored by testing interaction terms, including time-by-age and time-by-asthma status for RCSMS and time-by-age for ACSMS within the asthma subgroup.

To provide a distribution-free confirmation of within-subject changes, complementary non-parametric analyses were conducted for all six scores. Paired comparisons between baseline (T0) and the first-year follow-up (T1) were evaluated using the Wilcoxon signed-rank test, and the corresponding effect size (r) was calculated. For patients with data available at three or more consecutive timepoints, within-subject changes over time were assessed using the Friedman test. When overall differences were detected, post hoc pairwise comparisons were performed using the Nemenyi test. Effect size for Friedman analyses was reported as Kendall’s W. Long-term within-subject analyses were conducted in available-case subsets including patients with complete data at each follow-up time point.

The overall time effects from the MMRM analyses were considered the primary inferential analyses. Within each outcome, model-based pairwise comparisons with the baseline were adjusted using the Sidak method. Complementary non-parametric analyses were considered supportive, and post hoc pairwise comparisons following a significant Friedman test were adjusted using the Nemenyi procedure. Age-stratified analyses were conducted separately in mutually exclusive pediatric and adult datasets and were considered secondary analyses. No global multiplicity adjustment was applied across the six clinical outcomes or across age strata; therefore, results from the non-parametric and age-stratified analyses should be interpreted as supportive rather than confirmatory. Formal comparisons of longitudinal trajectories between pediatric and adult patients were based on the time-by-age-group interaction in the overall MMRM.

In all hypothesis tests, the null hypothesis was rejected with a type I error or error α < 0.05. The interpretation of effect sizes followed established conventions for correlation-based metrics, applying thresholds as follows: small = 0.10, moderate = 0.30, and large ≥ 0.50 for all r-derived statistics, including Wilcoxon r and Kendall’s W [40,41].

In addition, the comparison by time points allowed for more detail, and between the time points, there was a significant difference in the distribution of the variables.

The representation of the results was performed through a boxplot, with the median represented by its numerical value, offering the possibility to visualize and quantify the effectiveness of AIT with *Dermatophagoides*/*L. destructor*.

Data analysis was performed with SPSS version 20.0 software^®^ (IBM Corp., Armonk, NY, USA) and XLSTAT Life Sciences version 2024.2.2.1422 software^®^ (Addinsoft, Paris, France).

## 5. Conclusions

In conclusion, this real-world study demonstrates that *Dermatophagoides* spp./*L. destructor* allergen immunotherapy is associated with a favorable safety profile and clinically meaningful, durable improvements in rhinoconjunctivitis and asthma control, including in pediatric patients. These results support its use as an effective long-term therapeutic strategy in the management of allergic respiratory diseases.

## Figures and Tables

**Table 1:** Evolution of the RCSMS, ACSMS, RSS and ASS across the study period.

	T0	T1	T2	T3	T4	T5
	RCSMS					
Mean (SD)	3.9 (0.9)	1.9 (0.8)	1.3 (0.9)	1.0 (0.9)	0.9 (0.9)	0.6 (0.8)
Median (Q1, Q3)	3.7 (3.3, 4.3)	1.7 (1.5, 2.3)	1.3 (0.5, 1.7)	1.0 (0, 1.6)	1.0 (0, 1.3)	0.2 (0, 1)
*n*	199	199	106	61	40	11
%Reduction vs. T0Median	-	54.0	64.9	73.0	73.0	94.6
Wilcoxon T0–T1	-	<0.0001 ^a^	-	-	-	-
Friedman T0–T5	-	-	<0.0001 ^b^	<0.0001 ^c^	<0.0001 ^d^	<0.0001 ^e^
Effect size	-	0.859 ^a^	0.822 ^b^	0.779 ^c^	0.751 ^d^	0.799 ^e^
Interpretation effect size	-	Large ^a^	Large ^b^	Large ^c^	Large ^d^	Large ^e^
	ACSMS					
Mean (SD)	2.6 (1.0)	1.4 (1.1)	1.1 (1.0)	0.7 (0.7)	0.5 (0.6)	0.6 (0.7)
Median (Q1, Q3)	2.5 (1.8, 3.4)	1.3 (0.5, 2.3)	0.5 (0.5, 2.0)	0.5 (0, 0.8)	0.5 (0, 0.5)	0.5 (0.3, 0.6)
*n*	125	125	70	39	28	7
%Reduction vs. T0Median	-	48.0	80.0	80.0	80.0	80.0
Wilcoxon T0–T1	-	<0.0001 ^a^	-	-	-	-
Friedman T0–T5	-	-	<0.0001 ^b^	<0.0001 ^c^	<0.0001 ^d^	0.03 ^e^
Effect size	-	0.789 ^a^	0.721 ^b^	0.650 ^c^	0.612 ^d^	0.354 ^e^
Interpretation effect size	-	Large ^a^	Large ^b^	Large ^c^	Large ^d^	Moderate ^e^
	RSS					
Mean (SD)	1.8 (0.5)	0.8 (0.5)	0.4 (0.5)	0.3 (0.4)	0.2 (0.3)	0.1 (0.2)
Median (Q1, Q3)	1.8 (1.5, 2.3)	0.7 (0.5, 1.0)	0.3 (0.0, 0.7)	0.0 (0.0, 0.7)	0.0 (0.0, 0.3)	0.0 (0.0, 0.1)
*n*	199	199	106	61	40	11
%Reduction vs. T0Median	-	63.4	80.2	100	100	100
Wilcoxon T0–T1	-	<0.0001 ^a^	-	-	-	-
Friedman T0–T5	-	-	<0.0001 ^b^	<0.0001 ^c^	<0.0001 ^d^	<0.0001 ^e^
Effect size	-	0.855 ^a^	0.833 ^b^	0.780 ^c^	0.765 ^d^	0.783 ^e^
Interpretation effect size	-	Large ^a^	Large ^b^	Large ^c^	Large ^d^	Large ^e^
	ASS					
Mean (SD)	1.3 (0.5)	0.6 (0.5)	0.4 (0.4)	0.2 (0.3)	0.1 (0.3)	0.1 (0.2)
Median (Q1, Q3)	1.3 (1.0, 1.8)	0.5 (0.3, 1.0)	0.3 (0.0, 0.5)	0.0 (0.0, 0.3)	0.0 (0.0, 0.1)	0.0 (0.0, 0.1)
*n*	125	125	70	39	28	7
%Reduction vs. T0Median	-	60.0	80.0	100	100	100
Wilcoxon T0–T1	-	<0.0001 ^a^	-	-	-	-
Friedman T0–T5	-	-	<0.0001 ^b^	<0.0001 ^c^	<0.0001 ^d^	0.003 ^e^
Effect size	-	0.765 ^a^	0.730 ^b^	0.658 ^c^	0.642 ^d^	0.509 ^e^
Interpretation effect size	-	Large ^a^	Large ^b^	Large ^c^	Large ^d^	Large ^e^

^a^ Comparison performed in patients with available data at baseline and year 1. ^b^ Comparison performed in patients with available data at baseline and year 2. ^c^ Comparison performed in patients with available data at baseline and year 3. ^d^ Comparison performed in patients with available data at baseline and year 4. ^e^ Comparison performed in patients with available data at baseline and year 5. Effect sizes correspond to the Wilcoxon signed-rank test (T0–T1) or the Friedman test (T0–T5) within each respective subgroup. RCSMS: Rhinoconjunctivitis Symptom and Medication Score. ACSMS: Asthma Symptom and Medication Score. RSS: Rhinoconjunctivitis Symptom Score. ASS: Asthma Symptom Score. SD: Standard deviation. Q1: First quartile. Q3: Third quartile.

**Table 2:** Evolution of the RCSMS, ACSMS, RSS, and ASS across the study period in pediatric patients.

	T0	T1	T2	T3	T4	T5
	RCSMS					
Mean (SD)	4.0 (0.9)	1.8 (0.7)	0.9 (0.8)	0.5 (0.8)	0.3 (0.4)	0.3 (0.6)
Median (Q1, Q3)	3.7 (3.3, 4.4)	1.7 (1.5, 2.2)	1.0 (0, 1.3)	0.2 (0, 0.7)	0.2 (0, 0.7)	0.0 (0, 0.5)
*n*	76	76	34	18	15	3
%Reduction vs. T0Median	-	54.5	72.8	95.4	95.4	100.0
Wilcoxon T0–T1	-	<0.0001 ^a^	-	-	-	-
Friedman T0–T5	-	-	<0.0001 ^b^	<0.0001 ^c^	<0.0001 ^d^	<0.022 ^e^
Effect size	-	0.861 ^a^	0.914 ^b^	0.872 ^c^	0.890 ^d^	0.877 ^e^
Interpretation effect size	-	Large ^a^	Large ^b^	Large ^c^	Large ^d^	Large ^e^
	ACSMS					
Mean (SD)	2.6 (1.0)	1.4 (1.1)	1.1 (1.0)	0.7 (0.7)	0.5 (0.6)	0.6 (0.7)
Median (Q1, Q3)	2.5 (1.8, 3.4)	1.3 (0.5, 2.3)	0.5 (0.5, 2.0)	0.5 (0, 0.8)	0.5 (0, 0.5)	0.5 (0.3, 0.6)
*n*	50	50	23	12	12	2
%Reduction vs. T0Median	-	48.0	80.0	80.0	80.0	80.0
Wilcoxon T0–T1	-	<0.0001 ^a^	-	-	-	-
Friedman T0–T5	-	-	<0.0001 ^b^	<0.0001 ^c^	<0.0001 ^d^	0.105 ^e^
Effect size	-	0.782 ^a^	0.909 ^b^	0.875 ^c^	0.835 ^d^	0.910 ^e^
Interpretation effect size	-	Large ^a^	Large ^b^	Large ^c^	Large ^d^	Large ^e^
	RSS					
Mean (SD)	1.9 (0.5)	0.7 (0.4)	0.3 (0.3)	0.1 (0.2)	0.1 (0.1)	0.0 (0.0)
Median (Q1, Q3)	1.8 (1.5, 2.3)	0.7 (0.5, 1.0)	0.2 (0.0, 0.5)	0.0 (0, 0.2)	0.0 (0.0, 0.1)	0.0 (0.0, 0.0)
*n*	76	76	34	18	15	3
%Reduction vs. T0Median	-	63.4	89.8	100	100	100
Wilcoxon T0–T1	-	<0.0001 ^a^	-	-	-	-
Friedman T0–T5	-	-	<0.0001 ^b^	<0.0001 ^c^	<0.0001 ^d^	0.022 ^e^
Effect size	-	0.855 ^a^	0.918 ^b^	0.873 ^c^	0.865 ^d^	0.877 ^e^
Interpretation effect size	-	Large ^a^	Large ^b^	Large ^c^	Large ^d^	Large ^e^
	ASS					
Mean (SD)	1.3 (0.5)	0.5 (0.6)	0.2 (0.3)	0.0 (0.1)	0.0 (0.1)	0.0 (0.0)
Median (Q1, Q3)	1.3 (1.0, 1.5)	0.5 (0.0, 0.8)	0.0 (0.0, 0.3)	0.0 (0.0, 0.0)	0.0 (0.0, 0.0)	0.0 (0.0, 0.0)
*n*	50	50	23	12	12	2
%Reduction vs. T0Median	-	60.0	100	100	100	100
Wilcoxon T0–T1	-	<0.0001 ^a^	-	-	-	-
Friedman T0–T5	-	-	<0.0001 ^b^	<0.0001 ^c^	<0.0001 ^d^	0.142 ^e^
Effect size	-	0.729 ^a^	0.886 ^b^	0.844 ^c^	0.836 ^d^	0.826 ^e^
Interpretation effect size	-	Large ^a^	Large ^b^	Large ^c^	Large ^d^	Large ^e^

^a^ Comparison performed in patients with available data at baseline and year 1. ^b^ Comparison performed in patients with available data at baseline and year 2. ^c^ Comparison performed in patients with available data at baseline and year 3. ^d^ Comparison performed in patients with available data at baseline and year 4. ^e^ Comparison performed in patients with available data at baseline and year 5. Effect sizes correspond to the Wilcoxon signed-rank test (T0–T1) or the Friedman test (T0–T5) within each respective subgroup. RCSMS: Rhinoconjunctivitis Symptom and Medication Score. ACSMS: Asthma Symptom and Medication Score. RSS: Rhinoconjunctivitis Symptom Score. ASS: Asthma Symptom Score. SD: Standard deviation. Q1: First quartile. Q3: Third quartile.

## Data Availability

The aggregated data supporting the findings of this study are available within the article and its Supplementary Materials. Individual-level data are not publicly available due to privacy and ethical restrictions associated with the use of patient medical records.

## Supplementary Materials

The following supporting information can be downloaded at: https://www.mdpi.com/article/10.3390/ijms27167397/s1.

## Abbreviations

The following abbreviations are used in this manuscript:

ACSMS	Asthma Combined Symptom and Medication Score
AIT	Allergen Immunotherapy
AMS	Asthma Medication Score
AR	Adverse Reaction
AR(1)	First-Order Autoregressive Covariance Structure
ASS	Asthma Symptom Score
CI	Confidence Interval
CSMS	Combined Symptom and Medication Score
EAACI	European Academy of Allergy and Clinical Immunology
GEMA	Spanish Guideline on the Management of Asthma
HDM	House Dust Mite
IgE	Immunoglobulin E
IQR	Interquartile Range
LS-mean	Least-Squares Mean
MMRM	Mixed Model for Repeated Measures
MS	Medication Score
Q1	First Quartile
Q3	Third Quartile
RCSMS	Rhinoconjunctivitis Combined Symptom and Medication Score
REML	Restricted Maximum Likelihood
RMS	Rhinoconjunctivitis Medication Score
RSS	Rhinoconjunctivitis Symptom Score
SD	Standard Deviation
SE	Standard Error
sIgE	Specific Immunoglobulin E
SM	Storage Mite
SS	Symptom Score
T0–T5	Baseline and annual follow-up visits from year 1 to year 5
WAO	World Allergy Organization
