# Supplementary material for: Real-World Effectiveness and Safety of Allergen Immunotherapy with a Combination of Glutaraldehyde-Polymerized Allergen Extracts of Dermatophagoides spp. and Lepidoglyphus destructor in Adults and Children
Source: Int J Mol Sci. 2026 Aug 19;27(16):7397. doi: 10.3390/ijms27167397 (PMC13513068; doi:10.3390/ijms27167397)
Supplement: Supplementary file 1 [file ijms-27-07397-s001.zip › ijms-4489474-supplementary.pdf]

Supplementary Material

The present Supplementary Material provides box plots and descriptive tables for the total population and the pediatric subgroup, including only patients with complete follow-up data at each assessment time point. Results are shown for symptom scores, medication scores, and combined symptom–medication scores, including RSS, ASS, RMS, AMS, RCSMS, and ACSMS.

INDEX OF FIGURES

Figure S1. Estimated LS-means of the RCSMS over time obtained from the MMRM model (mean ± 95% CI). 9
Figure S2. Estimated LS-means of the RSS over time obtained from the MMRM model (mean ± 95% CI).......... 9
Figure S3. Estimated LS-means of the RMS over time obtained from the MMRM model (mean ± 95% CI).......... 10
Figure S4. Estimated LS-means of the ACSMS over time obtained from the MMRM model (mean ± 95% CI). 10
Figure S5. Estimated LS-means of the ASS over time obtained from the MMRM model (mean ± 95% CI).......... 11
Figure S6. Estimated LS-means of the AMS over time obtained from the MMRM model (mean ± 95% CI).......... 11
Figure S7. Estimated LS-means of the RCSMS over time in pediatric patients obtained from the MMRM model (mean ± 95% CI).......... 12
Figure S8. Estimated LS-means of the RSS over time in pediatric patients obtained from the MMRM model (mean ± 95% CI).......... 12
Figure S9. Estimated LS-means of the RMS over time in pediatric patients obtained from the MMRM model (mean ± 95% CI).......... 13
Figure S10. Estimated LS-means of the ACSMS over time in pediatric patients obtained from the MMRM model (mean ± 95% CI).......... 13
Figure S11. Estimated LS-means of the ASS over time in pediatric patients obtained from the MMRM model (mean ± 95% CI).......... 14
Figure S12. Estimated LS-means of the AMS over time in pediatric patients obtained from the MMRM model (mean ± 95% CI).......... 14
Figure S13. Box plot of the RSS at baseline (T0) and after one year of allergen immunotherapy (T1). 15
Figure S14. Box plot of the RSS at baseline, and after one and two years of immunotherapy in patients with complete two-year AIT data. 15
Figure S15. Box plot of the RSS at baseline, and after one, two, and three years of immunotherapy in patients with complete three-year AIT data.......... 16
Figure S16. Box plot of the RSS at baseline, and after one, two, three and four years of immunotherapy in patients with complete four-year AIT data.......... 16
Figure S17. Box plot of the RSS at baseline, and after one, two, three, four and five years of immunotherapy in patients with complete five-year AIT data.......... 17
Figure S18. Box plot of the ASS at baseline (T0) and after one year of allergen immunotherapy (T1). 17

Figure S19. Box plot of the ASS at baseline, and after one and two years of immunotherapy in patients with complete two-year AIT data. 18
Figure S20. Box plot of the ASS at baseline, and after one, two, and three years of immunotherapy in patients with complete three-year AIT data. 18
Figure S21. Box plot of the ASS at baseline, and after one, two, three and four years of immunotherapy in patients with complete four-year AIT data. 19
Figure S22. Box plot of the ASS at baseline, and after one, two, three, four and five years of immunotherapy in patients with complete five-year AIT data. 19
Figure S23. Box plot of the RCSMS at baseline (T0) and after one year of allergen immunotherapy (T1). 20
Figure S24. Box plot of the RCSMS at baseline, and after one and two years of immunotherapy in patients with complete two-year AIT data. 20
Figure S25. Box plot of the RCSMS at baseline, and after one, two, and three years of immunotherapy in patients with complete three-year AIT data. 21
Figure S26. Box plot of the RCSMS at baseline, and after one, two, three and four years of immunotherapy in patients with complete four-year AIT data. 21
Figure S27. Box plot of the RCSMS at baseline, and after one, two, three, four and five years of immunotherapy in patients with complete five-year AIT data. 22
Figure S28. Box plot of the ACSMS at baseline (T0) and after one year of allergen immunotherapy (T1). 22
Figure S29. Box plot of the ACSMS at baseline, and after one and two years of immunotherapy in patients with complete two-year AIT data. 23
Figure S30. Box plot of the ACSMS at baseline, and after one, two, and three years of immunotherapy in patients with complete three-year AIT data. 23
Figure S31. Box plot of the ACSMS at baseline, and after one, two, three and four years of immunotherapy in patients with complete four-year AIT data. 24
Figure S32. Box plot of the ACSMS at baseline, and after one, two, three, four and five years of immunotherapy in patients with complete five-year AIT data. 24
Figure S33. Box plot of the RSS at baseline (T0) and after one year of allergen immunotherapy (T1) in pediatric patients. 25
Figure S34. Box plot of the RSS at baseline, and after one and two years of immunotherapy in pediatric patients with complete two-year AIT data. 25
Figure S35. Box plot of the RSS at baseline, and after one, two, and three years of immunotherapy in pediatric patients with complete three-year AIT data. 26
Figure S36. Box plot of the RCSMS at baseline, and after one, two, three and four years of immunotherapy in pediatric patients with complete four-year AIT data. 26
Figure S37. Box plot of the RSS at baseline, and after one, two, three, four and five years of immunotherapy in pediatric patients with complete five-year AIT data. 27
Figure S38. Box plot of the ASS at baseline (T0) and after one year of allergen immunotherapy (T1) in pediatric patients. 27
Figure S39. Box plot of the ASS at baseline, and after one and two years of immunotherapy in pediatric patients with complete two-year AIT data. 28
Figure S40. Box plot of the ASS at baseline, and after one, two, and three years of immunotherapy in pediatric patients with complete three-year AIT data. 28
Figure S41. Box plot of the ASS at baseline, and after one, two, three and four years of immunotherapy in pediatric patients with complete four-year AIT data. 29
Figure S42. Box plot of the ASS at baseline, and after one, two, three, four and five years of immunotherapy in pediatric patients with complete five-year AIT data. 29

Figure S43. Box plot of the RCSMS at baseline (T0) and after one year of allergen immunotherapy (T1) in pediatric patients. 30
Figure S44. Box plot of the RCSMS at baseline, and after one and two years of immunotherapy in pediatric patients with complete two-year AIT data. 30
Figure S45. Box plot of the RCSMS at baseline, and after one, two, and three years of immunotherapy in pediatric patients with complete three-year AIT data. 31
Figure S46. Box plot of the RCSMS at baseline, and after one, two, three and four years of immunotherapy in pediatric patients with complete four-year AIT data. 31
Figure S47. Box plot of the RCSMS at baseline, and after one, two, three, four and five years of immunotherapy in pediatric patients with complete five-year AIT data. 32
Figure S48. Box plot of the ACSMS at baseline (T0) and after one year of allergen immunotherapy (T1) in pediatric patients. 32
Figure S49. Box plot of the ACSMS at baseline, and after one and two years of immunotherapy in pediatric patients with complete two-year AIT data. 33
Figure S50. Box plot of the ACSMS at baseline, and after one, two, and three years of immunotherapy in pediatric patients with complete three-year AIT data. 33
Figure S51. Box plot of the ACSMS at baseline, and after one, two, three and four years of immunotherapy in pediatric patients with complete four-year AIT data. 34
Figure S52. Box plot of the ACSMS at baseline, and after one, two, three, four and five years of immunotherapy in pediatric patients with complete five-year AIT data. 34

INDEX OF TABLES

Table S1. Demographic data from all participants and pediatric participants included in the study. 6
Table S2. Least-squares mean estimates (SE and 95% CI) and differences versus T0 for rhinoconjunctivitis and asthma outcomes over time from the mixed model for repeated measures (MMRM). All comparisons were statistically significant ($p < 0.05$). 7
Table S3. Least-squares mean estimates (SE and 95% CI) and differences versus T0 for rhinoconjunctivitis and asthma outcomes over time from the MMRM in pediatric patients. All comparisons were statistically significant ($p < 0.05$). 8
Table S4. Distribution of symptom and medication severity categories over the study period. 35
Table S5. Distribution of symptom and medication severity categories over the study period in pediatric patients. 36
Table S6. Descriptive statistics of the RCSMS at baseline (T0) and after one year of allergen immunotherapy (T1). 37
Table S7. Descriptive statistics of the RCSMS in the subset of patients who completed two years of AIT. 37
Table S8. Descriptive statistics of the RCSMS in the subset of patients who completed three years of AIT. 38
Table S9. Descriptive statistics of the RCSMS in the subset of patients who completed four years of AIT. 38
Table S10. Descriptive statistics of the RCSMS in the subset of patients who completed five years of AIT. 39
Table S11. Descriptive statistics of the ACSMS at baseline (T0) and after one year of allergen immunotherapy (T1). 39

Table S12. Descriptive statistics of the ACSMS in the subset of patients who completed two years of AIT. 40
Table S13. Descriptive statistics of the ACSMS in the subset of patients who completed three years of AIT. 40
Table S14. Descriptive statistics of the ACSMS in the subset of patients who completed four years of AIT. 41
Table S15. Descriptive statistics of the ACSMS in the subset of patients who completed five years of AIT. 41
Table S16. Descriptive statistics of the RSS at baseline (T0) and after one year of allergen immunotherapy (T1). 42
Table S17. Descriptive statistics of the RSS in the subset of patients who completed two years of AIT. 42
Table S18. Descriptive statistics of the RSS in the subset of patients who completed three years of AIT. 43
Table S19. Descriptive statistics of the RSS in the subset of patients who completed four years of AIT. 43
Table S20. Descriptive statistics of the RSS in the subset of patients who completed five years of AIT. 44
Table S21. Descriptive statistics of the ASS at baseline (T0) and after one year of allergen immunotherapy (T1). 44
Table S22. Descriptive statistics of the ASS in the subset of patients who completed two years of AIT. 45
Table S23. Descriptive statistics of the ASS in the subset of patients who completed three years of AIT. 45
Table S24. Descriptive statistics of the ASS in the subset of patients who completed four years of AIT. 46
Table S25. Descriptive statistics of the ASS in the subset of patients who completed five years of AIT. 46
Table S26. Descriptive statistics of the RCSMS at baseline (T0) and after one year of allergen immunotherapy (T1) in pediatric patients. 47
Table S27. Descriptive statistics of the RCSMS in the subset of pediatric patients who completed two years of AIT. 47
Table S28. Descriptive statistics of the RCSMS in the subset of pediatric patients who completed three years of AIT. 48
Table S29. Descriptive statistics of the RCSMS in the subset of pediatric patients who completed four years of AIT. 48
Table S30. Descriptive statistics of the RCSMS in the subset of pediatric patients who completed five years of AIT. 49
Table S31. Descriptive statistics of the ACSMS at baseline (T0) and after one year of allergen immunotherapy (T1) in pediatric patients. 49
Table S32. Descriptive statistics of the ACSMS in the subset of pediatric patients who completed two years of AIT. 50
Table S33. Descriptive statistics of the ACSMS in the subset of pediatric patients who completed three years of AIT. 50
Table S34. Descriptive statistics of the ACSMS in the subset of pediatric patients who completed four years of AIT. 51
Table S35. Descriptive statistics of the ACSMS in the subset of pediatric patients who completed five years of AIT. 51

Table S36. Descriptive statistics of the RSS at baseline (T0) and after one year of allergen immunotherapy (T1) in pediatric patients. 52
Table S37. Descriptive statistics of the RSS in the subset of pediatric patients who completed two years of AIT. 52
Table S38. Descriptive statistics of the RSS in the subset of pediatric patients who completed three years of AIT. 53
Table S39. Descriptive statistics of the RSS in the subset of pediatric patients who completed four years of AIT. 53
Table S40. Descriptive statistics of the RSS in the subset of pediatric patients who completed five years of AIT. 54
Table S41. Descriptive statistics of the ASS at baseline (T0) and after one year of allergen immunotherapy (T1) in pediatric patients. 54
Table S42. Descriptive statistics of the ASS in the subset of pediatric patients who completed two years of AIT. 55
Table S43. Descriptive statistics of the ASS in the subset of pediatric patients who completed three years of AIT. 55
Table S44. Descriptive statistics of the ASS in the subset of pediatric patients who completed four years of AIT. 56
Table S45. Descriptive statistics of the ASS in the subset of pediatric patients who completed five years of AIT. 56

Table S1. Demographic data from all participants and pediatric participants included in the study.

	All	Male	Female	Asthma	No asthma
**Mean age**	25.7	23.8	28.2	25.5	26.1
**C.I. <95%**	23.8	21.1	25.6	23	23
**C.I. >95%**	27.6	26.4	30.8	27.9	29.2
**S.D.**	13.5	14.2	12.1	13.7	13.3
**Median**	23	17	27,5	22	26.5
**Quartile 1**	14.5	12	19	15	14.3
**Quartile 3**	37	36	37	38	35
**Maximum**	59	59	55	59	55
**Minimum**	6	6	6	6	6
**n**	199	113	86	125	74
**Pediatric Patients**					
**Mean age**	12.3	12	13	12.5	11.8
**C.I. <95%**	11.5	11.1	11.3	11.5	10.4
**C.I. >95%**	13.1	12.9	14.7	13.5	13.1
**S.D.**	3.5	3.5	3.5	3.6	3.4
**Median**	12	12	13	12.5	11.5
**Quartile 1**	9	9	10	9	10
**Quartile 3**	16	16	16	16	14.8
**Maximum**	17	17	17	17	17
**Minimum**	6	6	6	6	6
**n**	76	57	19	50	26

S.D., Standard Deviation. C.I., Confidence Interval.

Table S2. Least-squares mean estimates (SE and 95% CI) and differences versus T0 for rhinoconjunctivitis and asthma outcomes over time from the mixed model for repeated measures (MMRM). All comparisons were statistically significant ($p < 0.05$).

Time	RCSMS				RSS				RMS			
	LS-mean	SE	95% CI	% Reduction *vs* T0	LS-mean	SE	95% CI	% Reduction *vs* T0	LS-mean	SE	95% CI	% Reduction *vs* T0
**T0**	1.85	0.06	1.77 – 1.92	-	1.85	0.04	1.77 - 1.92	-	2.01	0.04	1.94 - 2.08	-
**T1**	0.78	0.06	0.71 – 0.86	50.70	0.78	0.04	0.71 - 0.86	57.51	1.12	0.04	1.04 - 1.19	44.45
**T2**	0.48	0.10	0.39 – 0.58	66.19	0.48	0.05	0.39 - 0.58	73.77	0.80	0.06	0.68 - 0.93	60.03
**T3**	0.36	0.13	0.25 – 0.47	73.12	0.36	0.06	0.25 - 0.47	80.54	0.63	0.09	0.46 - 0.81	68.44
**T4**	0.27	0.17	0.12 – 0.42	99.98	0.27	0.08	0.12 - 0.42	85.31	0.62	0.11	0.39 - 0.84	69.29
**T5**	-	-	-	-	-	-	-	-	-	-	-	-
**Time**	**ACSMS**				**ASS**				**AMS**			
	LS-mean	SE	95% CI	% Reduction *vs* T0	LS-mean	SE	95% CI	% Reduction *vs* T0	LS-mean	SE	95% CI	% Reduction *vs* T0
**T0**	2.62	0.09	2.44 - 2.79	-	1.27	0.04	1.19 - 1.36	-	1.34	0.06	1.22 - 1.46	-
**T1**	1.49	0.10	1.29 - 1.68	43.14	0.60	0.05	0.51 - 0.69	52.79	0.89	0.06	0.76 - 1.01	33.98
**T2**	1.09	0.11	0.88 - 1.30	58.39	0.38	0.05	0.27 - 0.48	70.54	0.71	0.07	0.58 - 0.84	46.80
**T3**	0.73	0.11	0.52 - 0.95	72.08	0.21	0.06	0.10 - 0.33	83.19	0.52	0.07	0.38 - 0.65	61.62
**T4**	0.63	0.13	0.38 - 0.89	75.83	0.14	0.07	0.01 - 0.27	88.92	0.50	0.08	0.34 - 0.65	63.11
**T5**	0.287	0.27	-0.26 - 0.84	89.02	0.08	0.13	-0.18 - 0.35	93.40	0.15	0.18	-0.22 - 0.53	88.52

LS-mean: least-squares mean; SE: standard error; CI: confidence interval. RCSMS: Rhinoconjunctivitis Combined Symptom and Medication Score; RSS: Rhinoconjunctivitis Symptom Score; RMS: Rhinoconjunctivitis Medication Score; ACSMS: Asthma Combined Symptom and Medication Score; ASS: Asthma Symptom Score; AMS: Asthma Medication Score. LS-means were estimated using MMRM including time (categorical) as a fixed effect. For rhinoconjunctivitis outcomes, models additionally included age, gender and asthma status as fixed effects. Asthma outcomes were analysed in the asthma subgroup and included age and gender as fixed effects. Percent reduction represents the relative change in LS-mean values compared with baseline (T0).

Table S3. Least-squares mean estimates (SE and 95% CI) and differences versus T0 for rhinoconjunctivitis and asthma outcomes over time from the MMRM in pediatric patients. All comparisons were statistically significant ($p < 0.05$).

Time	RCSMS				RSS				RMS			
	LS-mean	SE	95% CI	% Reduction *vs* T0	LS-mean	SE	95% CI	% Reduction *vs* T0	LS-mean	SE	95% CI	% Reduction *vs* T0
**T0**	3.98	0.09	3.79 - 4.17	-	1.88	0.06	1.76 - 1.99	-	2.11	0.06	1.99 - 2.22	-
**T1**	1.81	0.08	1.65 - 1.97	54.49	0.73	0.04	0.64 - 0.82	60.94	1.08	0.05	0.98 - 1.18	48.75
**T2**	0.98	0.14	0.69 - 1.27	75.37	0.30	0.06	0.19 - 0.42	83.95	0.65	0.11	0.43 - 0.87	69.03
**T3**	0.67	0.19	0.28 - 1.06	83.15	0.16	0.08	0.01 - 0.32	91.21	0.45	0.14	0.18 - 0.73	78.55
**T4**	0.35	0.18	-0.01 - 0.71	91.28	0.07	0.08	-0.1 - 0.23	96.40	0.27	0.12	0.02 - 0.51	87.34
**T5**	0.50	0.44	-0.51 - 1.5	87.51	0.00	0.19	-0.37 - 0.37	100	0.37	0.30	-0.31 - 1.05	82.32

Time	ACSMS				ASS				AMS			
	LS-mean	SE	95% CI	% Reduction *vs* T0	LS-mean	SE	95% CI	% Reduction *vs* T0	LS-mean	SE	95% CI	% Reduction *vs* T0
**T0**	2.45	0.13	2.18 - 2.71	-	1.25	0.06	1.13 - 1.36	-	1.20	0.09	1.02 - 1.38	-
**T1**	1.31	0.15	1.01 - 1.62	46.42	0.53	0.08	0.36 - 0.70	57.43	0.78	0.09	0.61 - 0.95	35.00
**T2**	0.71	0.14	0.44 - 0.99	70.78	0.21	0.08	0.06 - 0.36	83.01	0.51	0.09	0.34 - 0.68	57.53
**T3**	0.37	0.16	0.05 - 0.70	84.67	0.04	0.09	-0.15 - 0.23	96.55	0.33	0.10	0.13 - 0.53	72.48
**T4**	0.29	0.16	-0.03 - 0.61	88.04	0.04	0.09	-0.14 - 0.23	96.51	0.25	0.10	0.05 - 0.45	79.21
**T5**	0.31	0.40	-0.49 - 1.11	87.24	0.00	0.23	-0.46 - 0.46	100.00	0.33	0.26	-0.18 - 0.85	72.13

LS-mean: least-squares mean; SE: standard error; CI: confidence interval. RCSMS: Rhinoconjunctivitis Combined Symptom and Medication Score; RSS: Rhinoconjunctivitis Symptom Score; RMS: Rhinoconjunctivitis Medication Score; ACSMS: Asthma Combined Symptom and Medication Score; ASS: Asthma Symptom Score; AMS: Asthma Medication Score. LS-means were estimated using MMRM including time (categorical) as a fixed effect. For rhinoconjunctivitis outcomes, models additionally included age, gender and asthma status as fixed effects. Asthma outcomes were analysed in the asthma subgroup and included age and gender as fixed effects. Percent reduction represents the relative change in LS-mean values compared with baseline (T0).

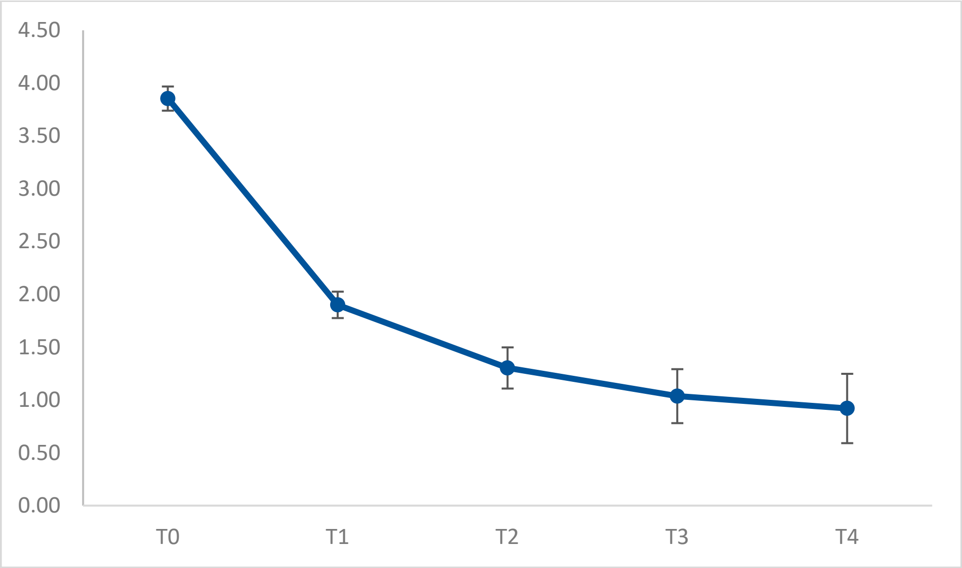

Figure S1. Estimated LS-means of the RCSMS over time obtained from the MMRM model (mean ± 95% CI).

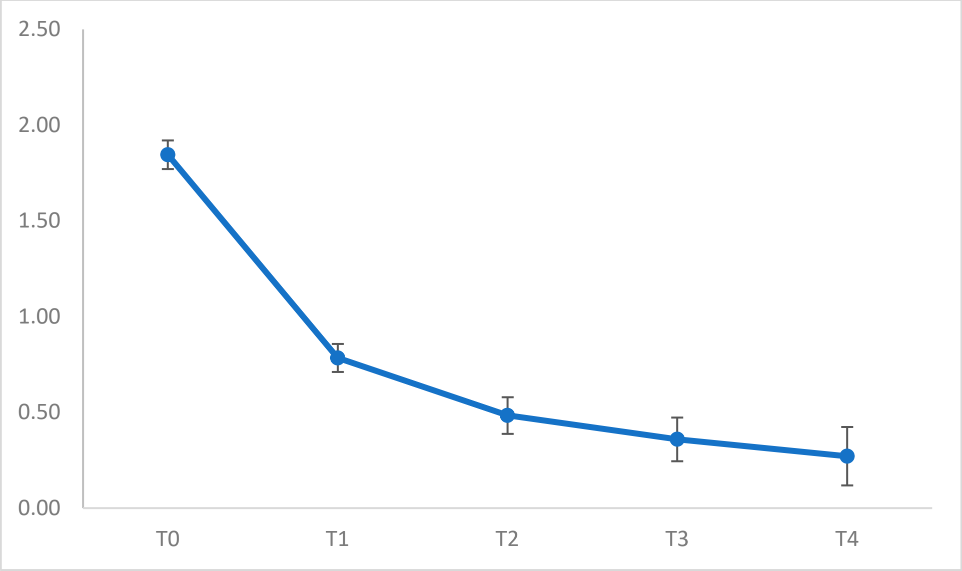

Figure S2. Estimated LS-means of the RSS over time obtained from the MMRM model (mean ± 95% CI).

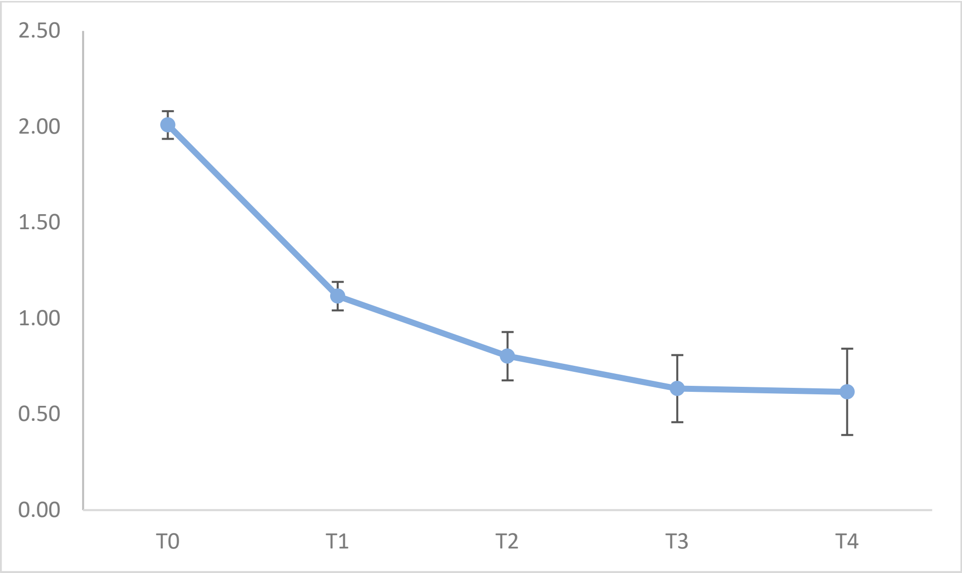

Figure S3. Estimated LS-means of the RMS over time obtained from the MMRM model (mean ± 95% CI).

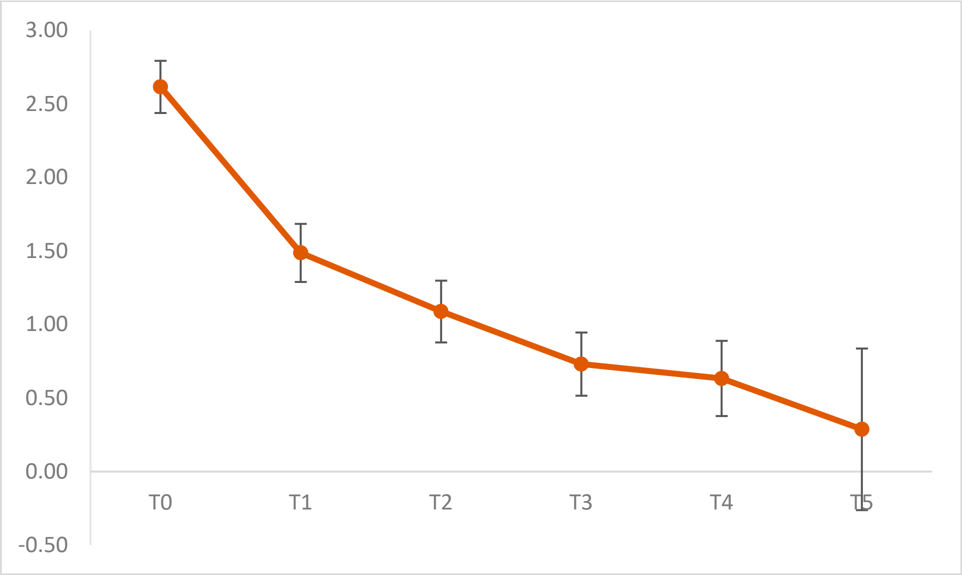

Figure S4. Estimated LS-means of the ACSMS over time obtained from the MMRM model (mean ± 95% CI).

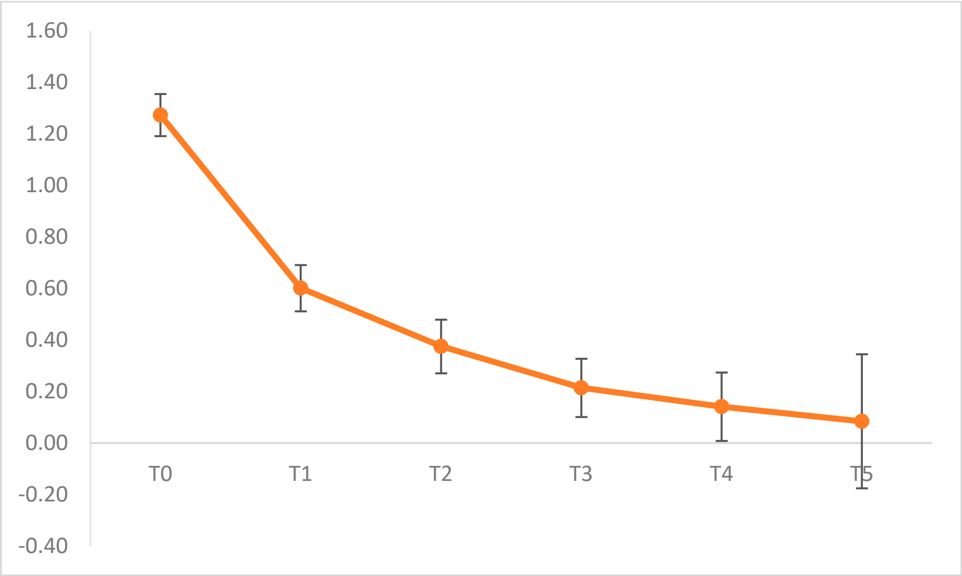

Figure S5. Estimated LS-means of the ASS over time obtained from the MMRM model (mean ± 95% CI).

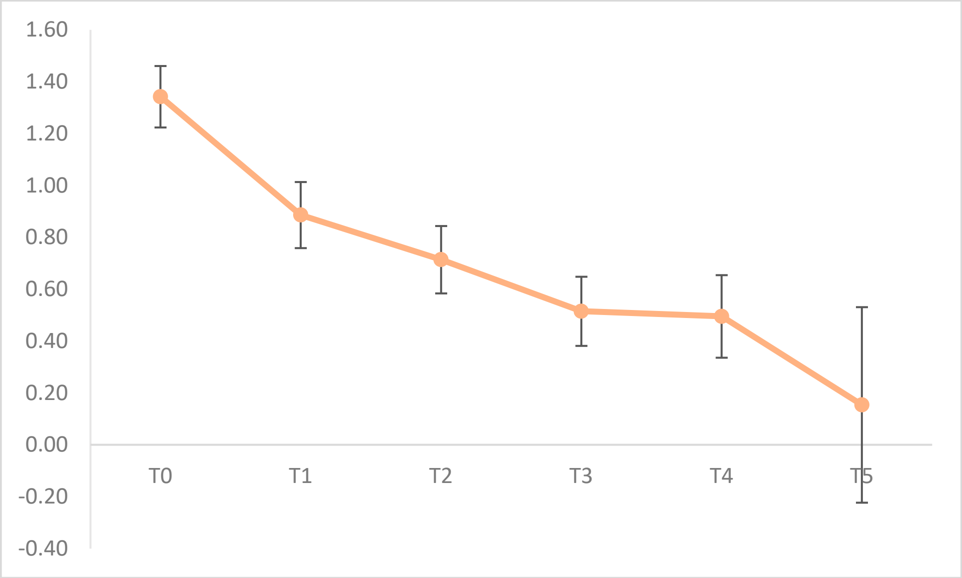

Figure S6. Estimated LS-means of the AMS over time obtained from the MMRM model (mean ± 95% CI).

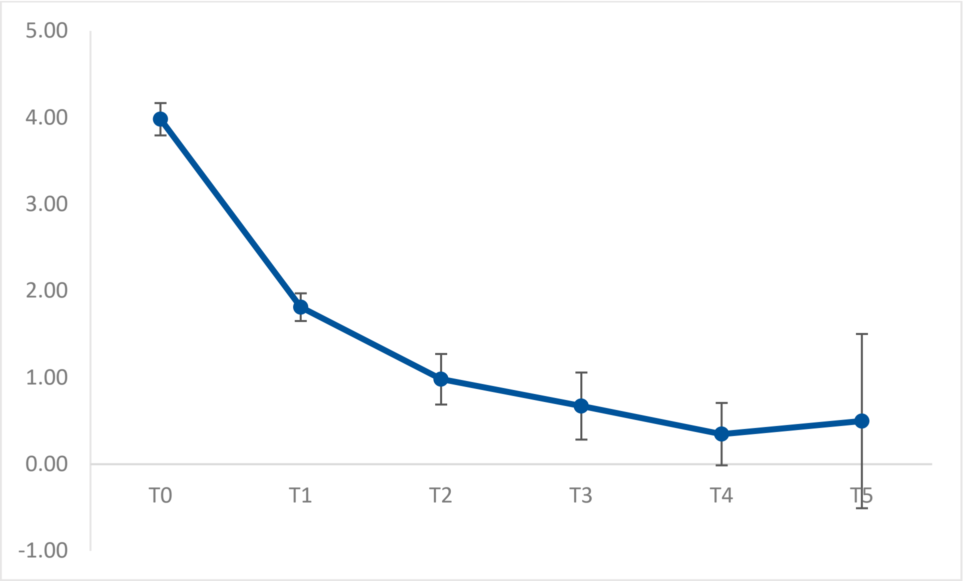

Figure S7. Estimated LS-means of the RCSMS over time in pediatric patients obtained from the MMRM model (mean ± 95% CI).

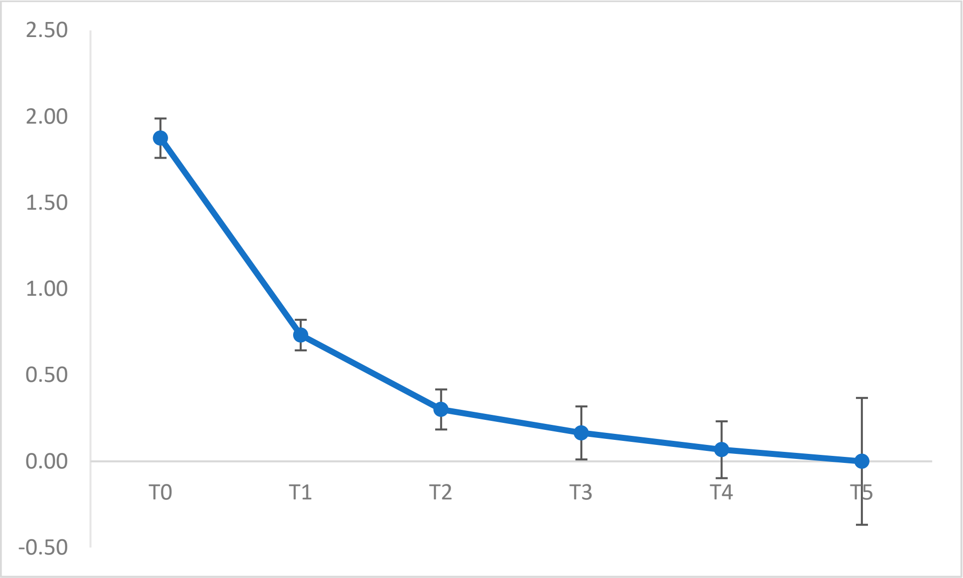

Figure S8. Estimated LS-means of the RSS over time in pediatric patients obtained from the MMRM model (mean ± 95% CI).

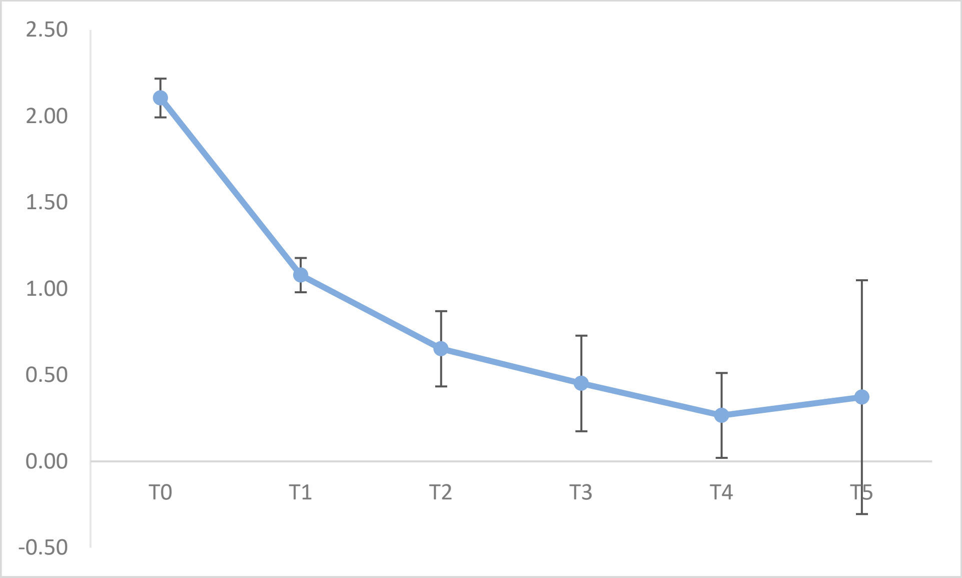

Figure S9. Estimated LS-means of the RMS over time in pediatric patients obtained from the MMRM model (mean ± 95% CI).

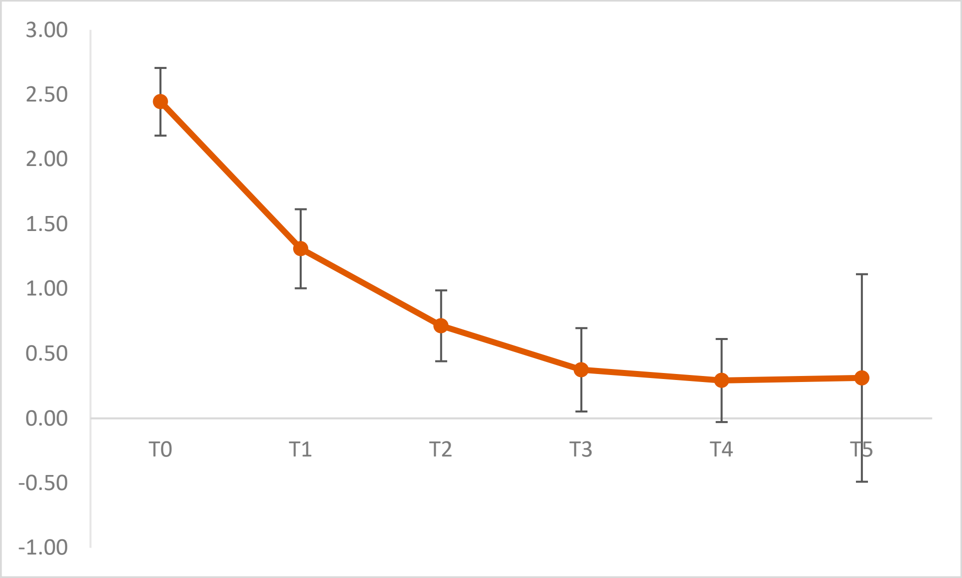

Figure S10. Estimated LS-means of the ACSMS over time in pediatric patients obtained from the MMRM model (mean ± 95% CI).

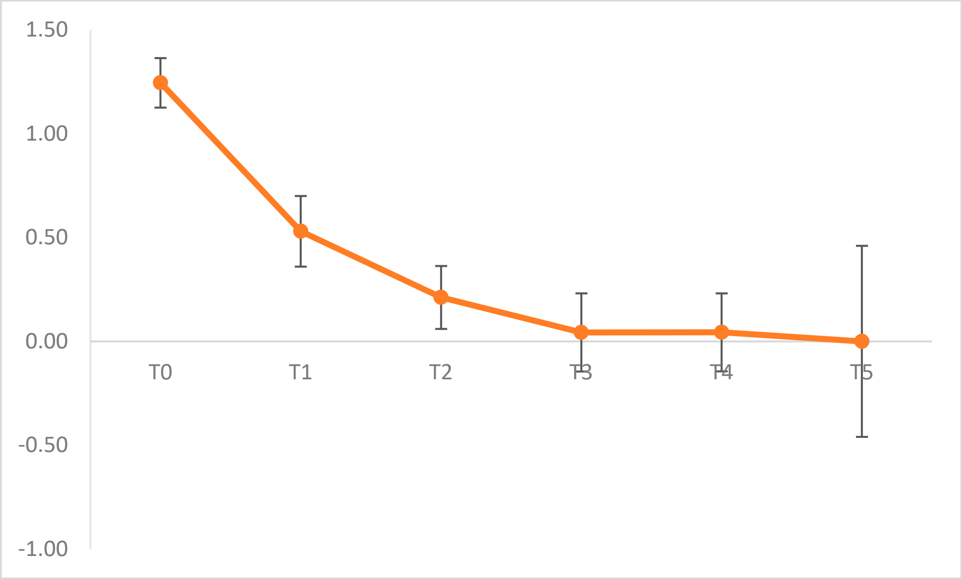

Figure S11. Estimated LS-means of the ASS over time in pediatric patients obtained from the MMRM model (mean ± 95% CI).

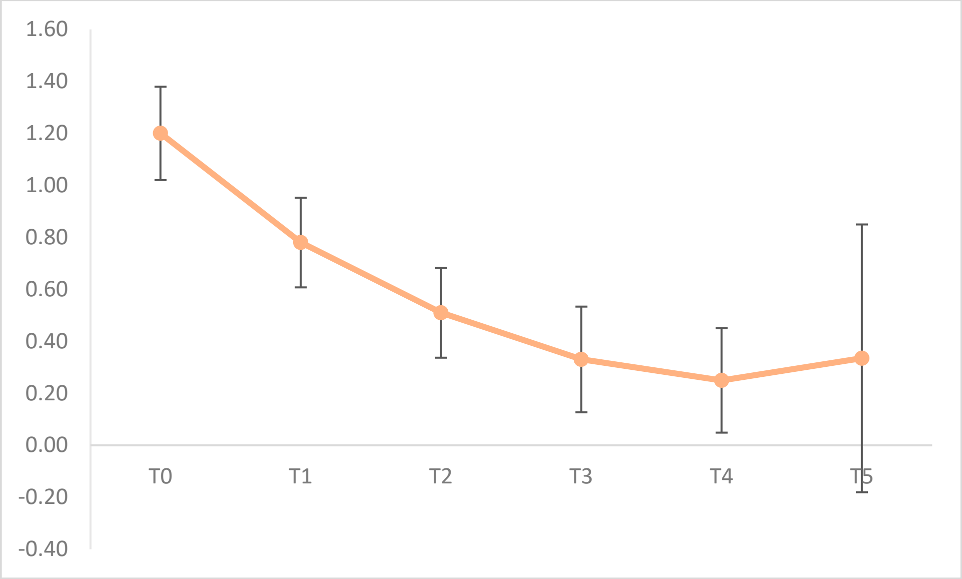

Figure S12. Estimated LS-means of the AMS over time in pediatric patients obtained from the MMRM model (mean ± 95% CI).

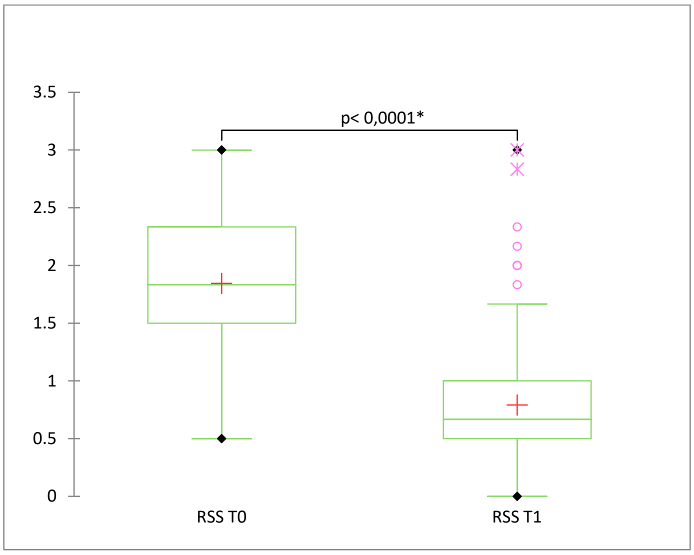

Figure S13. Box plot of the RSS at baseline (T0) and after one year of allergen immunotherapy (T1).

Boxes represent the interquartile range (IQR), the central horizontal line indicates the median, and whiskers denote minimum and maximum values. Red crosses indicate mean values, and pink symbols represent outliers. Pairwise comparisons between time points are shown with corresponding *p*-values. *Statistically significant differences compared with baseline (α = 0.05). RSS: Rhinoconjunctivitis Symptom Score.

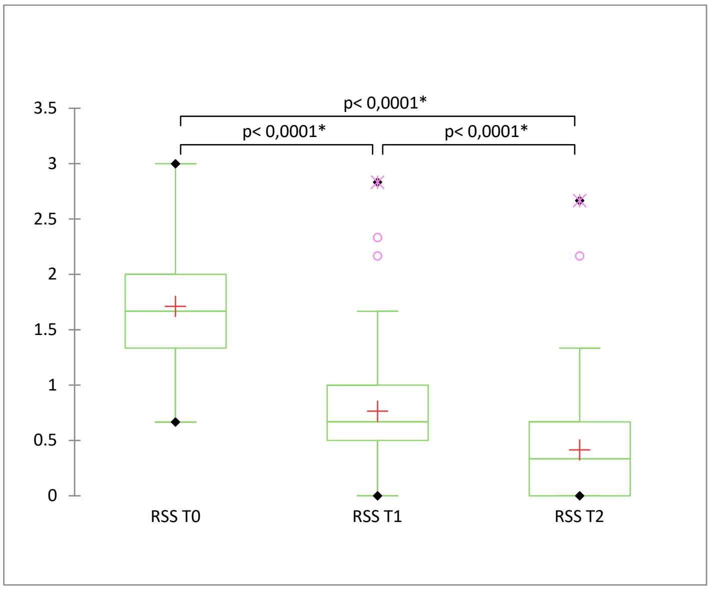

Figure S14. Box plot of the RSS at baseline, and after one and two years of immunotherapy in patients with complete two-year AIT data.

Boxes represent the interquartile range (IQR), the central horizontal line indicates the median, and whiskers denote minimum and maximum values. Red crosses indicate mean values, and pink symbols represent outliers. *Statistically significant differences compared with baseline (α = 0.05). RSS: Rhinoconjunctivitis Symptom Score.

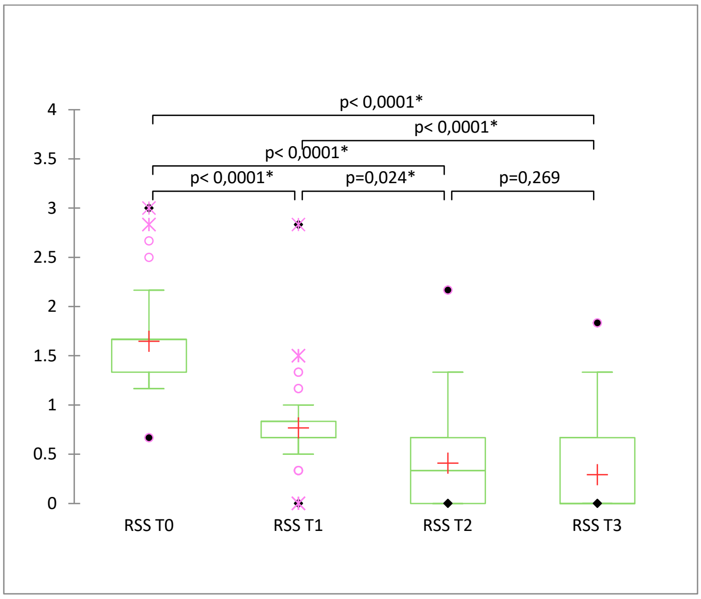

Figure S15. Box plot of the RSS at baseline, and after one, two, and three years of immunotherapy in patients with complete three-year AIT data.

Boxes represent the interquartile range (IQR), the central horizontal line indicates the median, and whiskers denote minimum and maximum values. Red crosses indicate mean values, and pink symbols represent outliers. Pairwise comparisons between time points are shown with corresponding p-values. *Statistically significant differences compared with baseline (α = 0.05). RSS: Rhinoconjunctivitis Symptom Score.

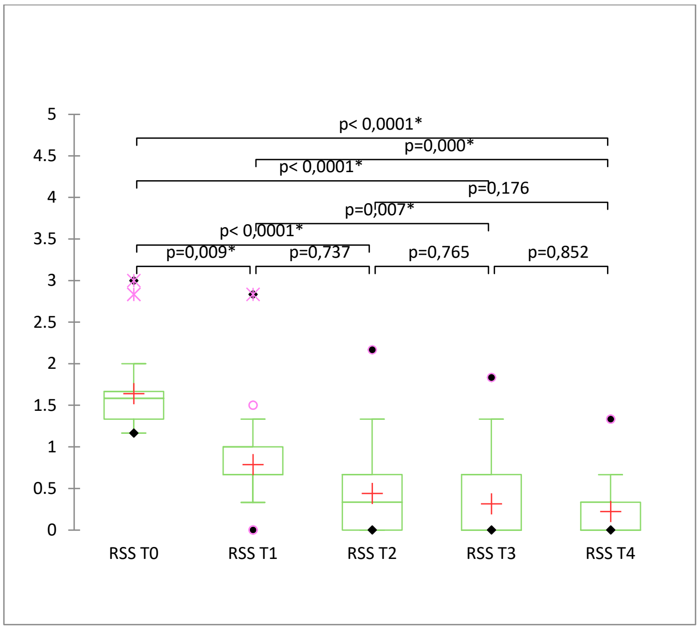

Figure S16. Box plot of the RSS at baseline, and after one, two, three and four years of immunotherapy in patients with complete four-year AIT data.

Boxes represent the interquartile range (IQR), the central horizontal line indicates the median, and whiskers denote minimum and maximum values. Red crosses indicate mean values, and pink symbols represent outliers. Pairwise comparisons between time points are shown with corresponding p-values. *Statistically significant differences compared with baseline (α = 0.05). RSS: Rhinoconjunctivitis Symptom Score.

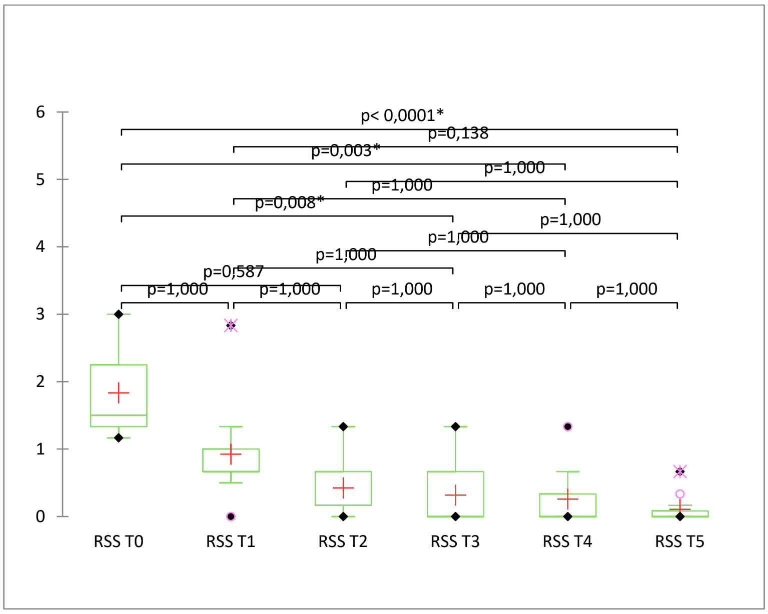

Figure S17. Box plot of the RSS at baseline, and after one, two, three, four and five years of immunotherapy in patients with complete five-year AIT data.

Boxes represent the interquartile range (IQR), the central horizontal line indicates the median, and whiskers denote minimum and maximum values. Red crosses indicate mean values, and pink symbols represent outliers. *Statistically significant differences compared with baseline (α = 0.05). RSS: Rhinoconjunctivitis Symptom Score.

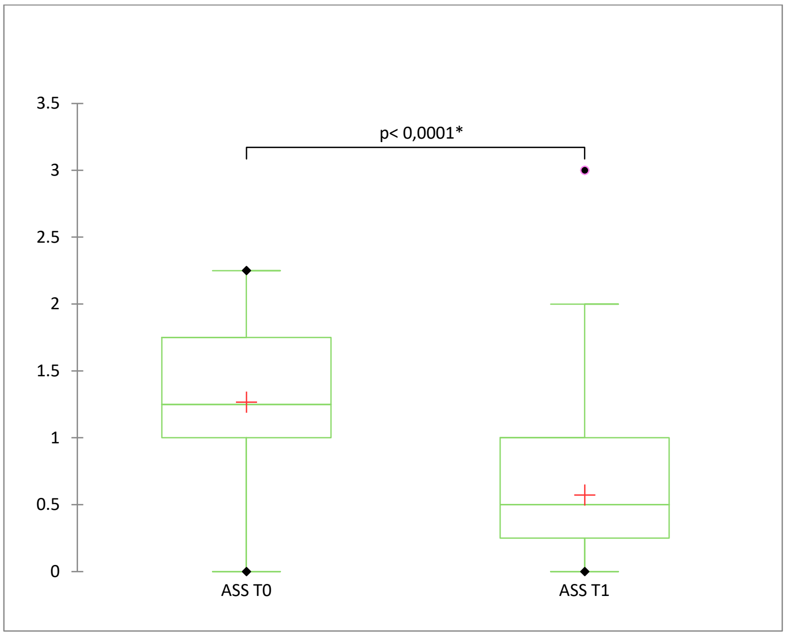

Figure S18. Box plot of the ASS at baseline (T0) and after one year of allergen immunotherapy (T1).

Boxes represent the interquartile range (IQR), the central horizontal line indicates the median, and whiskers denote minimum and maximum values. Red crosses indicate mean values, and pink symbols represent outliers. Pairwise comparisons between time points are shown with corresponding p-values. *Statistically significant differences compared with baseline (α = 0.05). ASS: Asthma Symptom Score.

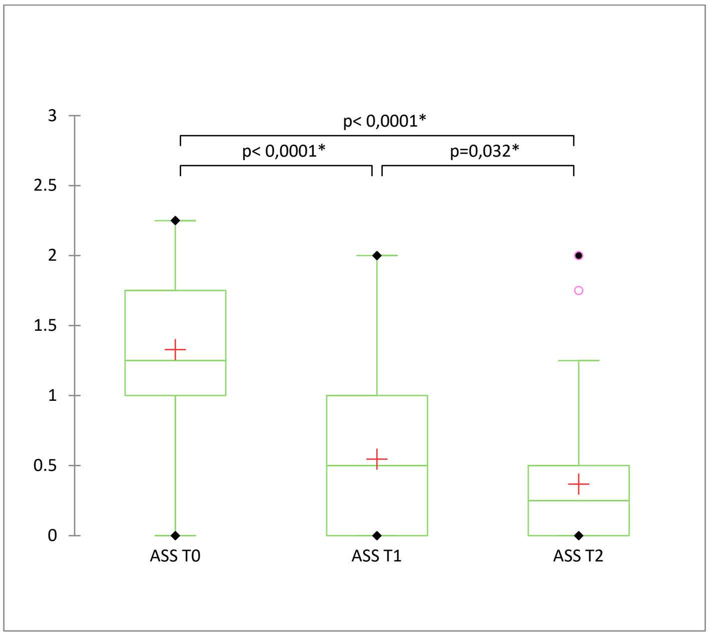

Figure S19. Box plot of the ASS at baseline, and after one and two years of immunotherapy in patients with complete two-year AIT data.

Boxes represent the interquartile range (IQR), the central horizontal line indicates the median, and whiskers denote minimum and maximum values. Red crosses indicate mean values, and pink symbols represent outliers. Pairwise comparisons between time points are shown with corresponding p-values. *Statistically significant differences compared with baseline (α = 0.05). ASS: Asthma Symptom Score.

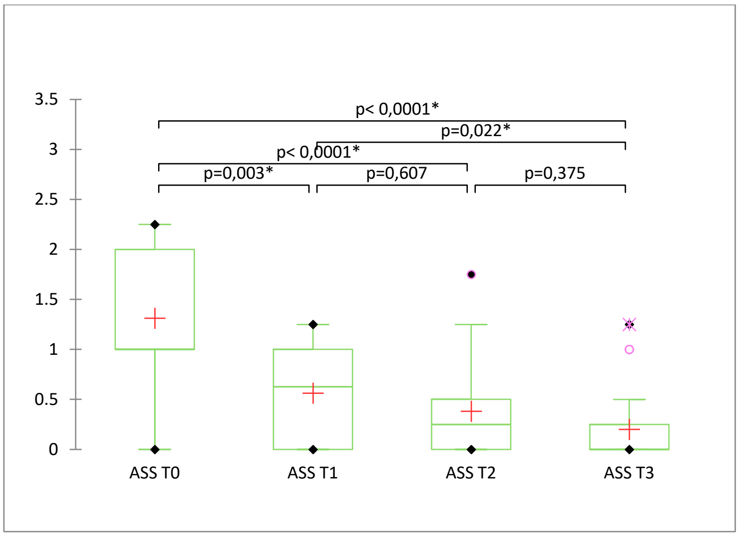

Figure S20. Box plot of the ASS at baseline, and after one, two, and three years of immunotherapy in patients with complete three-year AIT data.

Boxes represent the interquartile range (IQR), the central horizontal line indicates the median, and whiskers denote minimum and maximum values. Red crosses indicate mean values, and pink symbols represent outliers. Pairwise comparisons between time points are shown with corresponding p-values. *Statistically significant differences compared with baseline (α = 0.05). ASS: Asthma Symptom Score.

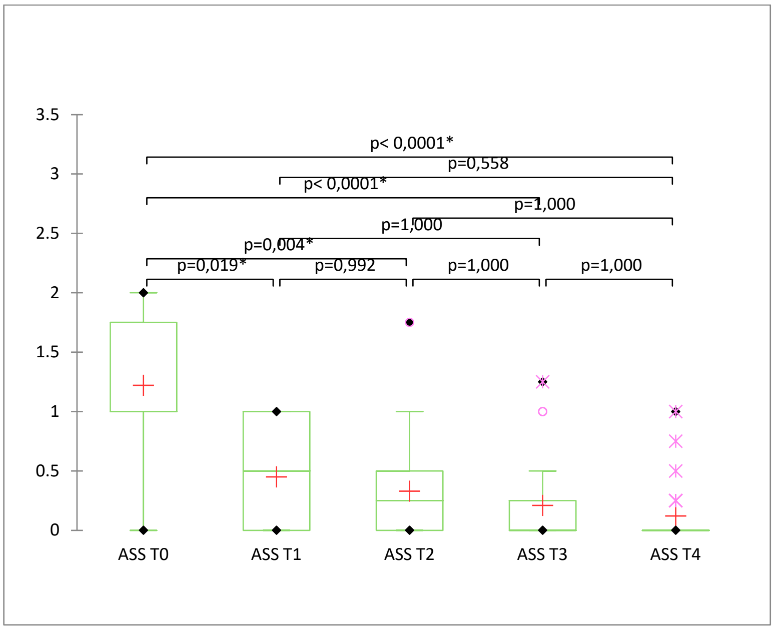

Figure S21. Box plot of the ASS at baseline, and after one, two, three and four years of immunotherapy in patients with complete four-year AIT data.

Boxes represent the interquartile range (IQR), the central horizontal line indicates the median, and whiskers denote minimum and maximum values. Red crosses indicate mean values, and pink symbols represent outliers. Pairwise comparisons between time points are shown with corresponding p-values. *Statistically significant differences compared with baseline (α = 0.05). ASS: Asthma Symptom Score.

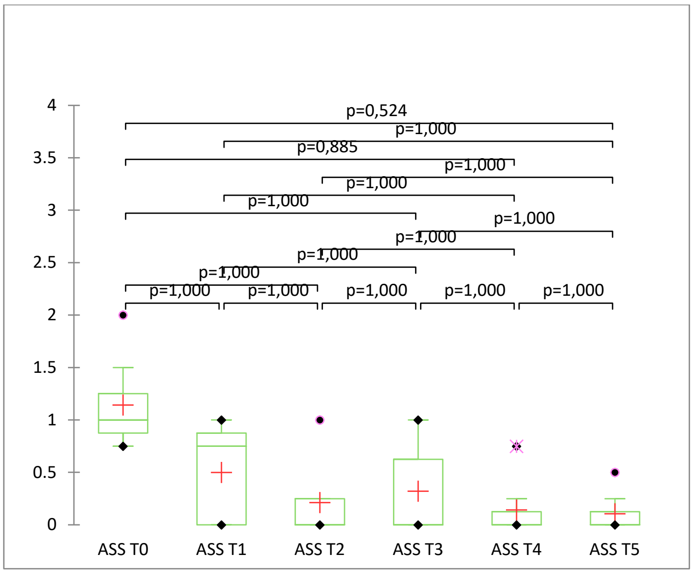

Figure S22. Box plot of the ASS at baseline, and after one, two, three, four and five years of immunotherapy in patients with complete five-year AIT data.

Boxes represent the interquartile range (IQR), the central horizontal line indicates the median, and whiskers denote minimum and maximum values. Red crosses indicate mean values, and pink symbols represent outliers. Pairwise comparisons between time points are shown with corresponding p-values. *Statistically significant differences compared with baseline (α = 0.05). ASS: Asthma Symptom Score.

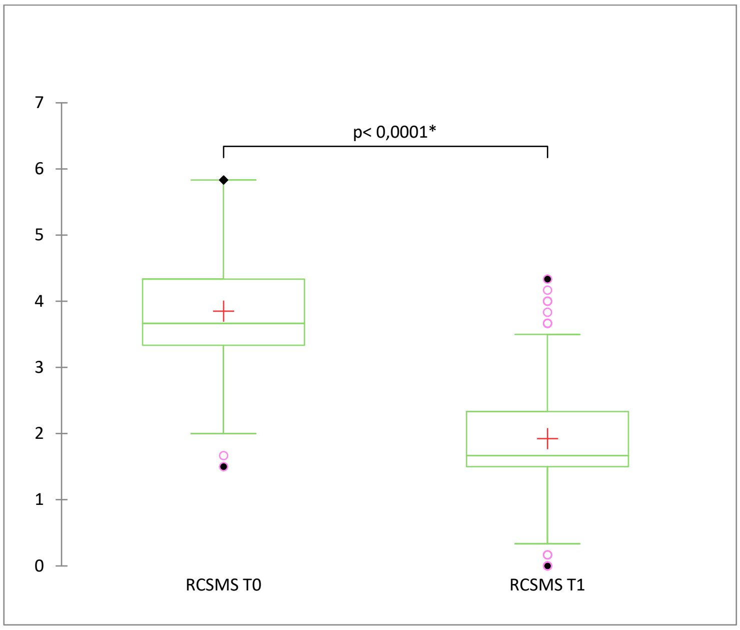

Figure S23. Box plot of the RCSMS at baseline (T0) and after one year of allergen immunotherapy (T1).

Boxes represent the interquartile range (IQR), the central horizontal line indicates the median, and whiskers denote minimum and maximum values. Red crosses indicate mean values, and pink symbols represent outliers. *Statistically significant differences compared with baseline (α = 0.05). RCSMS: Rhinoconjunctivitis Symptom and Medication Score.

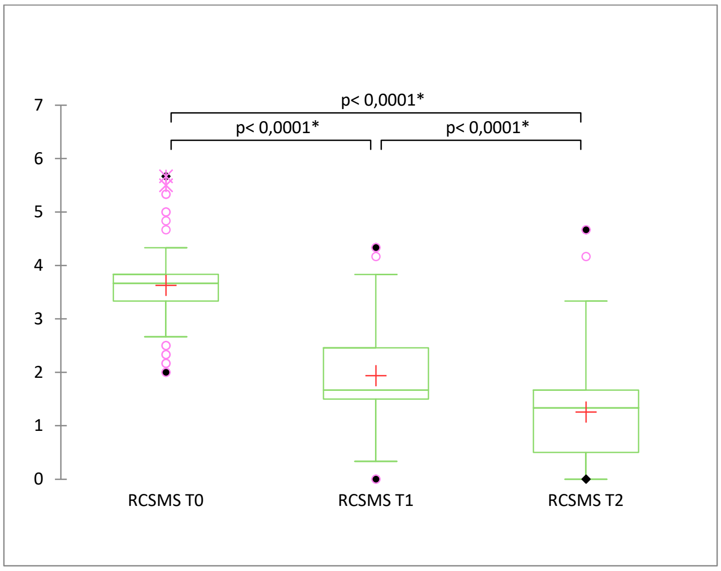

Figure S24. Box plot of the RCSMS at baseline, and after one and two years of immunotherapy in patients with complete two-year AIT data.

Boxes represent the interquartile range (IQR), the central horizontal line indicates the median, and whiskers denote minimum and maximum values. Red crosses indicate mean values, and pink symbols represent outliers. Pairwise comparisons between time points are shown with corresponding p-values. *Statistically significant differences compared with baseline (α = 0.05). RCSMS: Rhinoconjunctivitis Symptom and Medication Score.

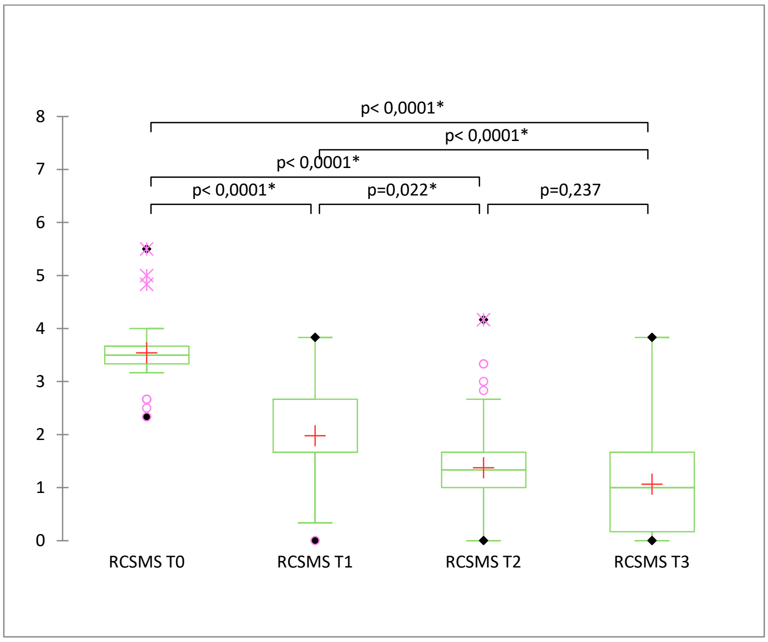

Figure S25. Box plot of the RCSMS at baseline, and after one, two, and three years of immunotherapy in patients with complete three-year AIT data.

Boxes represent the interquartile range (IQR), the central horizontal line indicates the median, and whiskers denote minimum and maximum values. Red crosses indicate mean values, and pink symbols represent outliers. Pairwise comparisons between time points are shown with corresponding p-values. *Statistically significant differences compared with baseline (α = 0.05). RCSMS: Rhinoconjunctivitis Symptom and Medication Score.

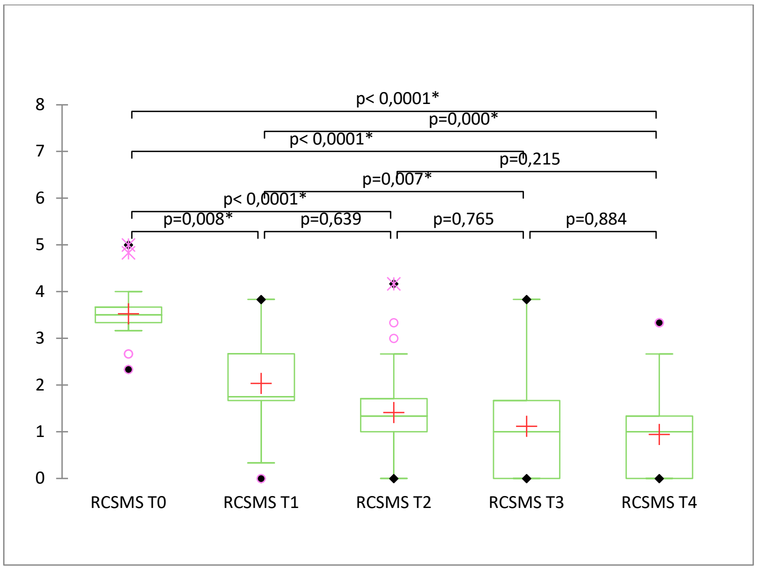

Figure S26. Box plot of the RCSMS at baseline, and after one, two, three and four years of immunotherapy in patients with complete four-year AIT data.

Boxes represent the interquartile range (IQR), the central horizontal line indicates the median, and whiskers denote minimum and maximum values. Red crosses indicate mean values, and pink symbols represent outliers. *Statistically significant differences compared with baseline (α = 0.05). RCSMS: Rhinoconjunctivitis Symptom and Medication Score.

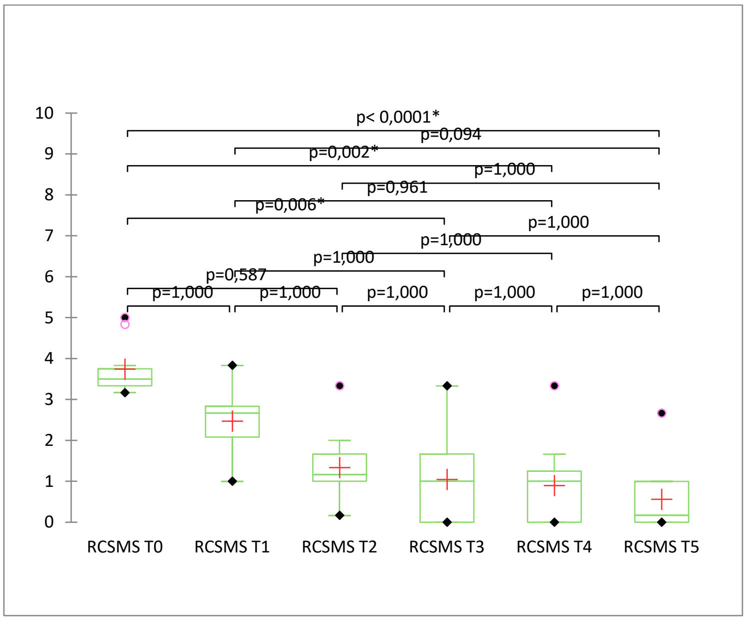

Figure S27. Box plot of the RCSMS at baseline, and after one, two, three, four and five years of immunotherapy in patients with complete five-year AIT data.

Boxes represent the interquartile range (IQR), the central horizontal line indicates the median, and whiskers denote minimum and maximum values. Red crosses indicate mean values, and pink symbols represent outliers. Pairwise comparisons between time points are shown with corresponding p-values. *Statistically significant differences compared with baseline (α = 0.05). RCSMS: Rhinoconjunctivitis Symptom and Medication Score.

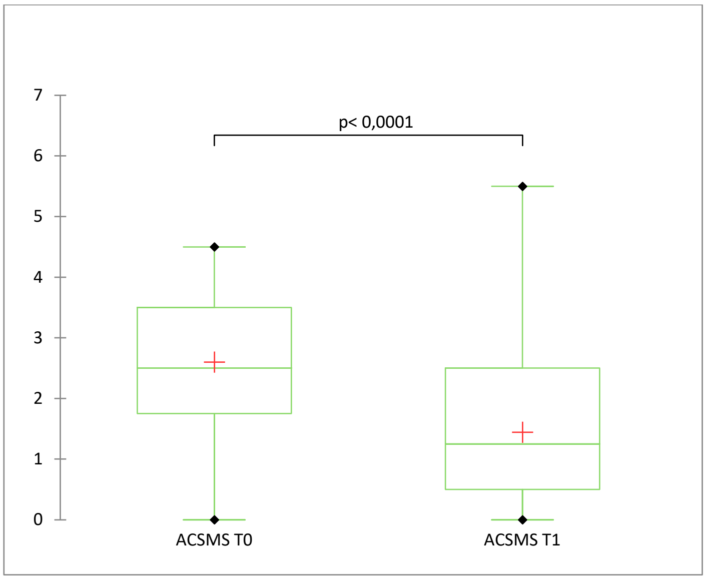

Figure S28. Box plot of the ACSMS at baseline (T0) and after one year of allergen immunotherapy (T1).

Boxes represent the interquartile range (IQR), the central horizontal line indicates the median, and whiskers denote minimum and maximum values. Red crosses indicate mean values, and pink symbols represent outliers. Pairwise comparisons between time points are shown with corresponding p-values. *Statistically significant differences compared with baseline (α = 0.05). ACSMS: Asthma Symptom and Medication Score.

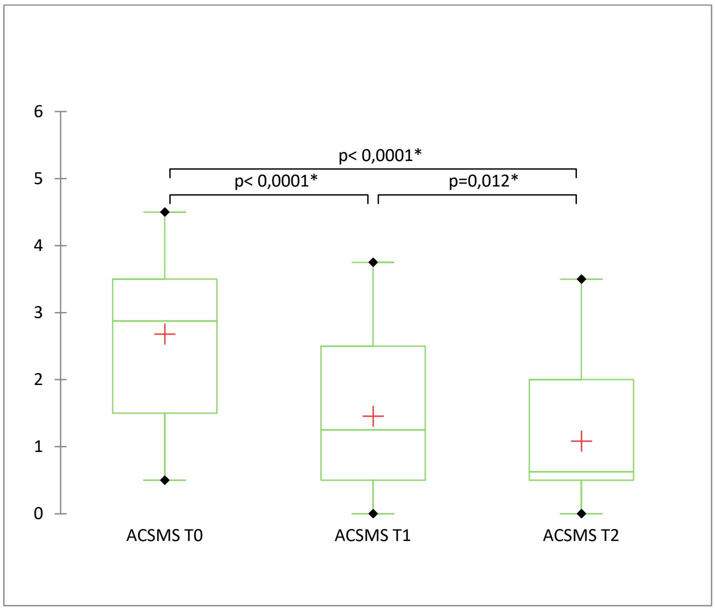

Figure S29. Box plot of the ACSMS at baseline, and after one and two years of immunotherapy in patients with complete two-year AIT data.

Boxes represent the interquartile range (IQR), the central horizontal line indicates the median, and whiskers denote minimum and maximum values. Red crosses indicate mean values, and pink symbols represent outliers. Pairwise comparisons between time points are shown with corresponding p-values. *Statistically significant differences compared with baseline (α = 0.05). ACSMS: Asthma Symptom and Medication Score.

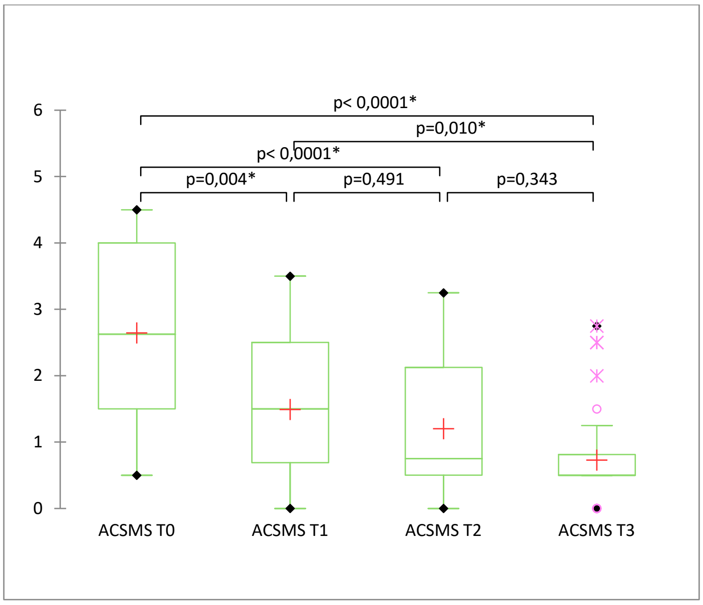

Figure S30. Box plot of the ACSMS at baseline, and after one, two, and three years of immunotherapy in patients with complete three-year AIT data.

Boxes represent the interquartile range (IQR), the central horizontal line indicates the median, and whiskers denote minimum and maximum values. Red crosses indicate mean values, and pink symbols represent outliers. Pairwise comparisons between time points are shown with corresponding p-values. *Statistically significant differences compared with baseline (α = 0.05). ACSMS: Asthma Symptom and Medication Score.

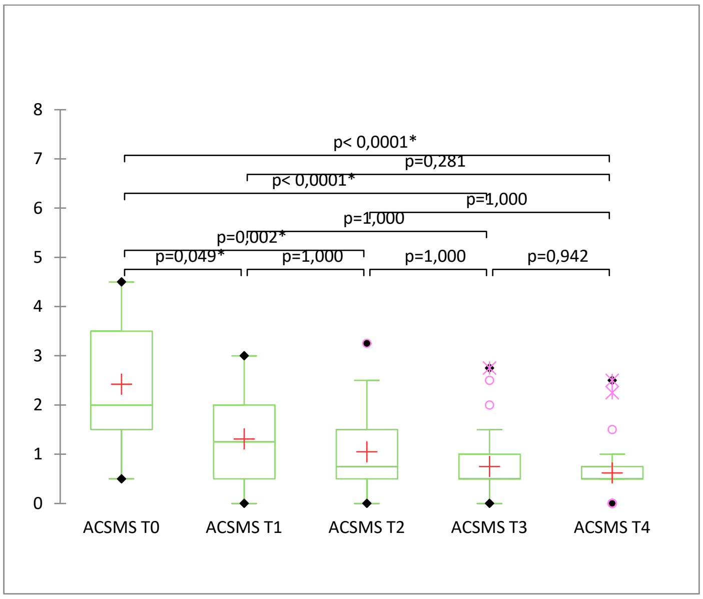

Figure S31. Box plot of the ACSMS at baseline, and after one, two, three and four years of immunotherapy in patients with complete four-year AIT data.

Boxes represent the interquartile range (IQR), the central horizontal line indicates the median, and whiskers denote minimum and maximum values. Red crosses indicate mean values, and pink symbols represent outliers. Pairwise comparisons between time points are shown with corresponding p-values. *Statistically significant differences compared with baseline (α = 0.05). ACSMS: Asthma Symptom and Medication Score.

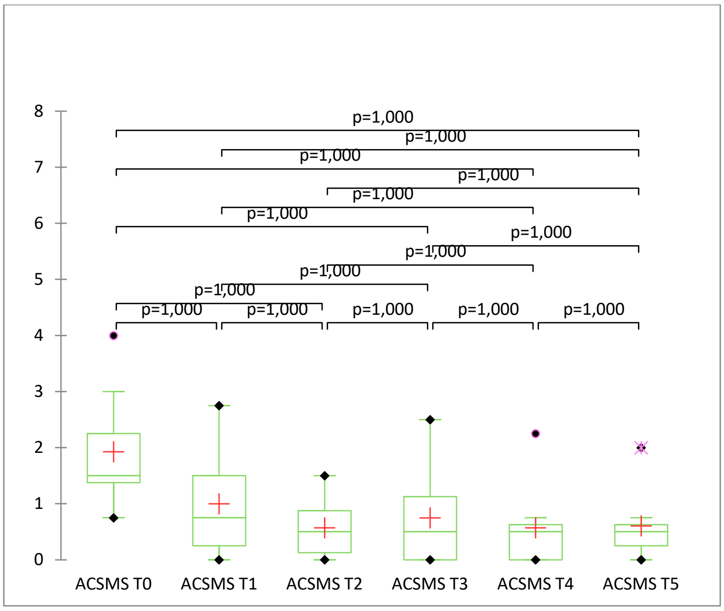

Figure S32. Box plot of the ACSMS at baseline, and after one, two, three, four and five years of immunotherapy in patients with complete five-year AIT data.

Boxes represent the interquartile range (IQR), the central horizontal line indicates the median, and whiskers denote minimum and maximum values. Red crosses indicate mean values, and pink symbols represent outliers. Pairwise comparisons between time points are shown with corresponding p-values. *Statistically significant differences compared with baseline (α = 0.05). ACSMS: Asthma Symptom and Medication Score.

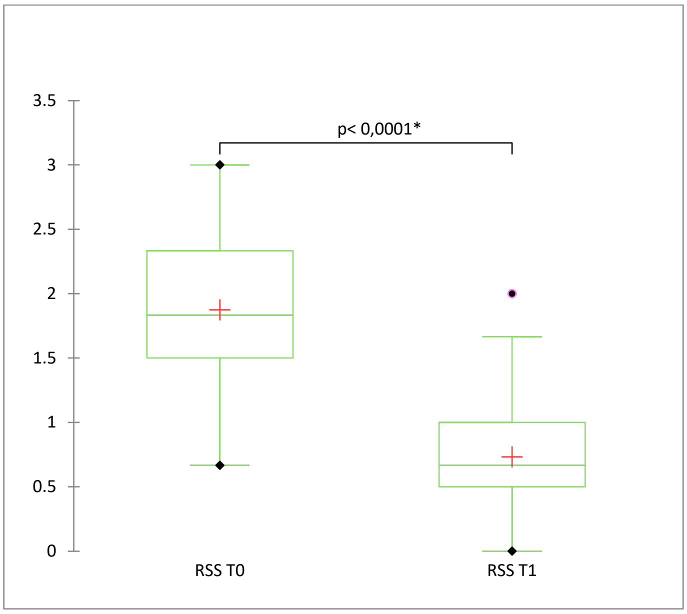

Figure S33. Box plot of the RSS at baseline (T0) and after one year of allergen immunotherapy (T1) in pediatric patients.

Boxes represent the interquartile range (IQR), the central horizontal line indicates the median, and whiskers denote minimum and maximum values. Red crosses indicate mean values, and pink symbols represent outliers. Pairwise comparisons between time points are shown with corresponding p-values. *Statistically significant differences compared with baseline (α = 0.05). RSS: Rhinoconjunctivitis Symptom Score.

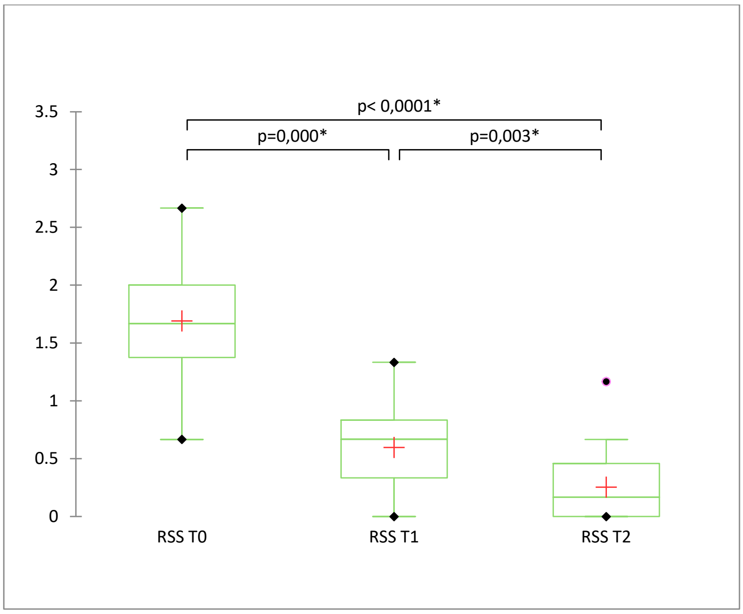

Figure S34. Box plot of the RSS at baseline, and after one and two years of immunotherapy in pediatric patients with complete two-year AIT data.

Boxes represent the interquartile range (IQR), the central horizontal line indicates the median, and whiskers denote minimum and maximum values. Red crosses indicate mean values, and pink symbols represent outliers. Pairwise comparisons between time points are shown with corresponding p-values. *Statistically significant differences compared with baseline (α = 0.05). RSS: Rhinoconjunctivitis Symptom Score.

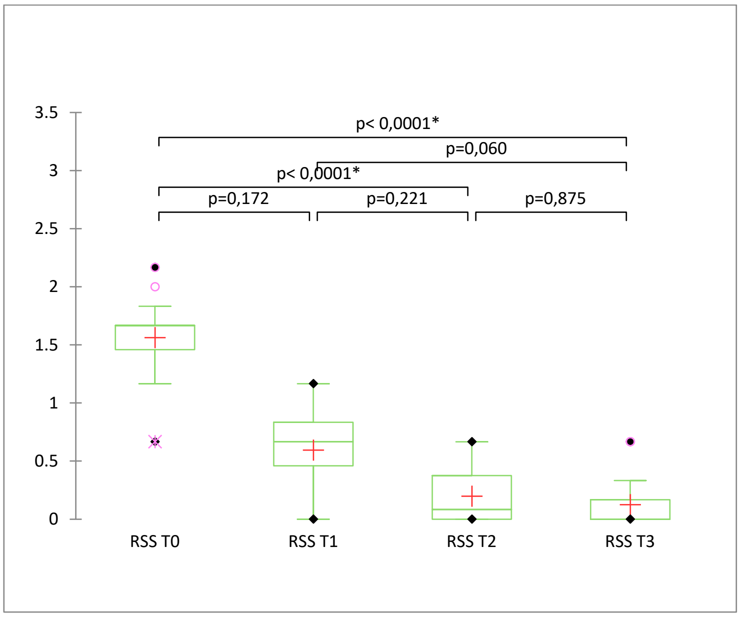

Figure S35. Box plot of the RSS at baseline, and after one, two, and three years of immunotherapy in pediatric patients with complete three-year AIT data.

Boxes represent the interquartile range (IQR), the central horizontal line indicates the median, and whiskers denote minimum and maximum values. Red crosses indicate mean values, and pink symbols represent outliers. *Statistically significant differences compared with baseline (α = 0.05). RSS: Rhinoconjunctivitis Symptom Score.

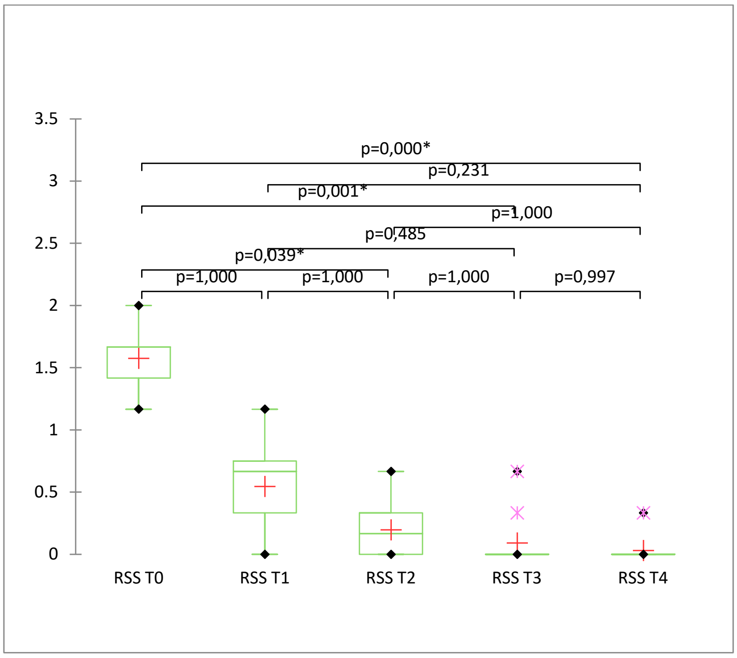

Figure S36. Box plot of the RCSMS at baseline, and after one, two, three and four years of immunotherapy in pediatric patients with complete four-year AIT data.

Boxes represent the interquartile range (IQR), the central horizontal line indicates the median, and whiskers denote minimum and maximum values. Red crosses indicate mean values, and pink symbols represent outliers. Pairwise comparisons between time points are shown with corresponding p-values. *Statistically significant differences compared with baseline (α = 0.05). RSS: Rhinoconjunctivitis Symptom Score.

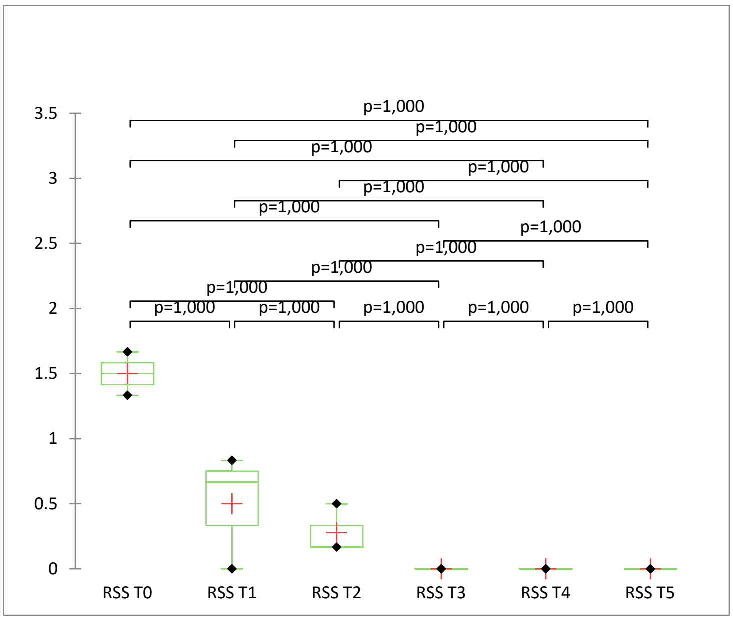

Figure S37. Box plot of the RSS at baseline, and after one, two, three, four and five years of immunotherapy in pediatric patients with complete five-year AIT data.

Boxes represent the interquartile range (IQR), the central horizontal line indicates the median, and whiskers denote minimum and maximum values. Red crosses indicate mean values, and pink symbols represent outliers. Pairwise comparisons between time points are shown with corresponding p-values. *Statistically significant differences compared with baseline (α = 0.05). RSS: Rhinoconjunctivitis Symptom Score.

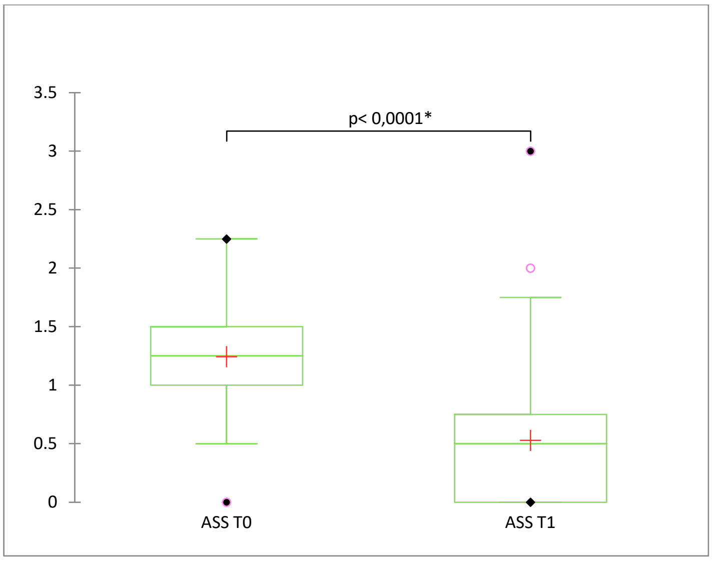

Figure S38. Box plot of the ASS at baseline (T0) and after one year of allergen immunotherapy (T1) in pediatric patients.

Boxes represent the interquartile range (IQR), the central horizontal line indicates the median, and whiskers denote minimum and maximum values. Red crosses indicate mean values, and pink symbols represent outliers. Pairwise comparisons between time points are shown with corresponding p-values. *Statistically significant differences compared with baseline (α = 0.05). ASS: Asthma Symptom Score.

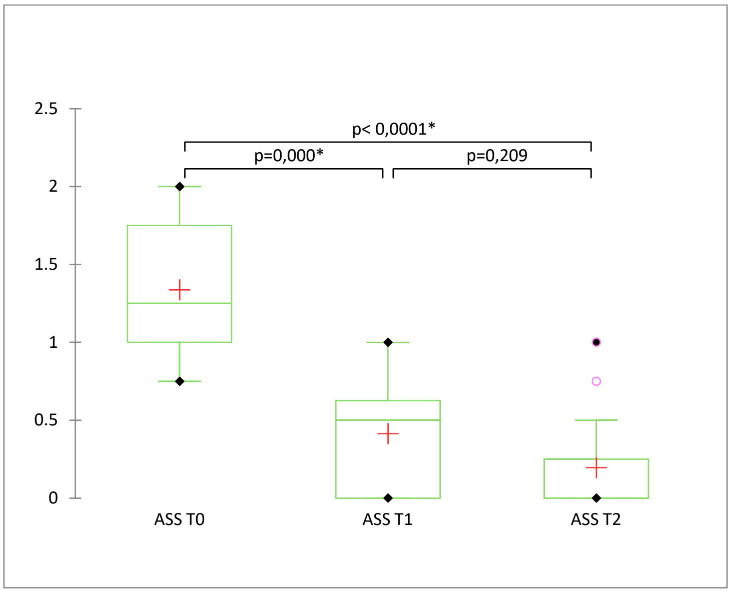

Figure S39. Box plot of the ASS at baseline, and after one and two years of immunotherapy in pediatric patients with complete two-year AIT data.

Boxes represent the interquartile range (IQR), the central horizontal line indicates the median, and whiskers denote minimum and maximum values. Red crosses indicate mean values, and pink symbols represent outliers. Pairwise comparisons between time points are shown with corresponding p-values. *Statistically significant differences compared with baseline (α = 0.05). ASS: Asthma Symptom Score.

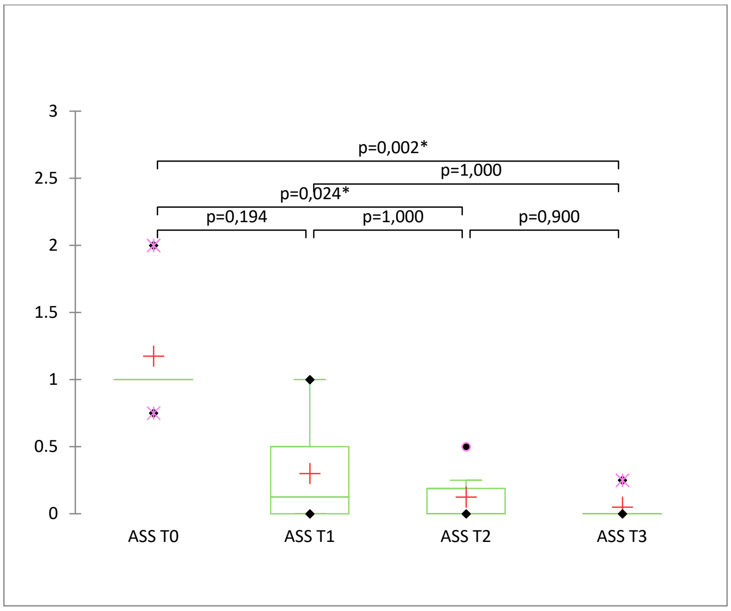

Figure S40. Box plot of the ASS at baseline, and after one, two, and three years of immunotherapy in pediatric patients with complete three-year AIT data.

Boxes represent the interquartile range (IQR), the central horizontal line indicates the median, and whiskers denote minimum and maximum values. Red crosses indicate mean values, and pink symbols represent outliers. Pairwise comparisons between time points are shown with corresponding p-values. *Statistically significant differences compared with baseline (α = 0.05). ASS: Asthma Symptom Score.

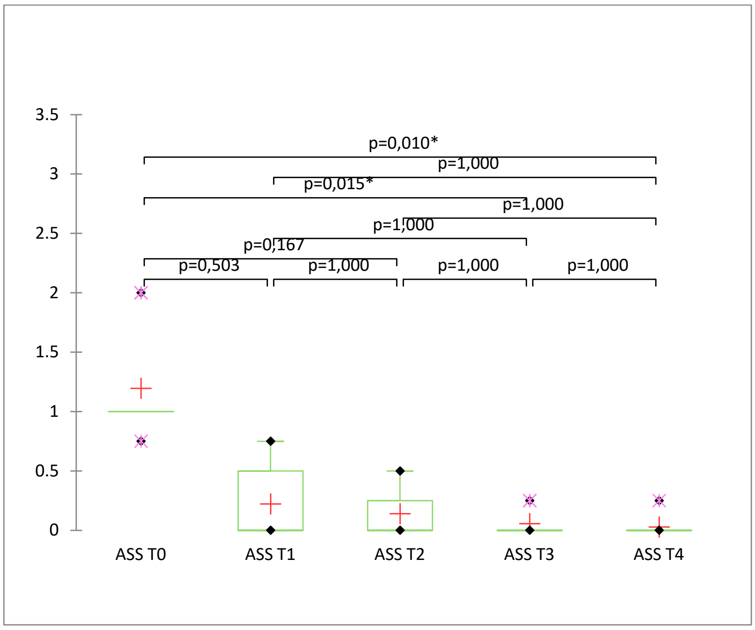

Figure S41. Box plot of the ASS at baseline, and after one, two, three and four years of immunotherapy in pediatric patients with complete four-year AIT data.

Boxes represent the interquartile range (IQR), the central horizontal line indicates the median, and whiskers denote minimum and maximum values. Red crosses indicate mean values, and pink symbols represent outliers. Pairwise comparisons between time points are shown with corresponding p-values. *Statistically significant differences compared with baseline (α = 0.05). ASS: Asthma Symptom Score.

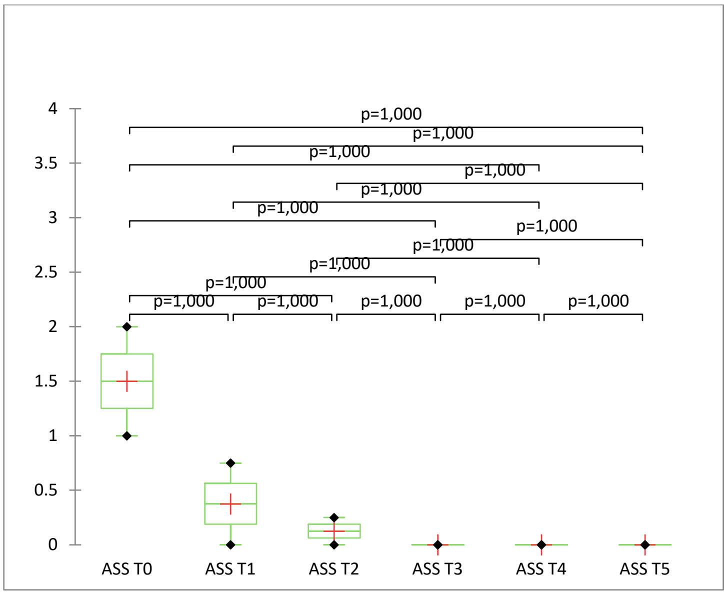

Figure S42. Box plot of the ASS at baseline, and after one, two, three, four and five years of immunotherapy in pediatric patients with complete five-year AIT data.

Boxes represent the interquartile range (IQR), the central horizontal line indicates the median, and whiskers denote minimum and maximum values. Red crosses indicate mean values, and pink symbols represent outliers. Pairwise comparisons between time points are shown with corresponding p-values. *Statistically significant differences compared with baseline (α = 0.05). ASS: Asthma Symptom Score.

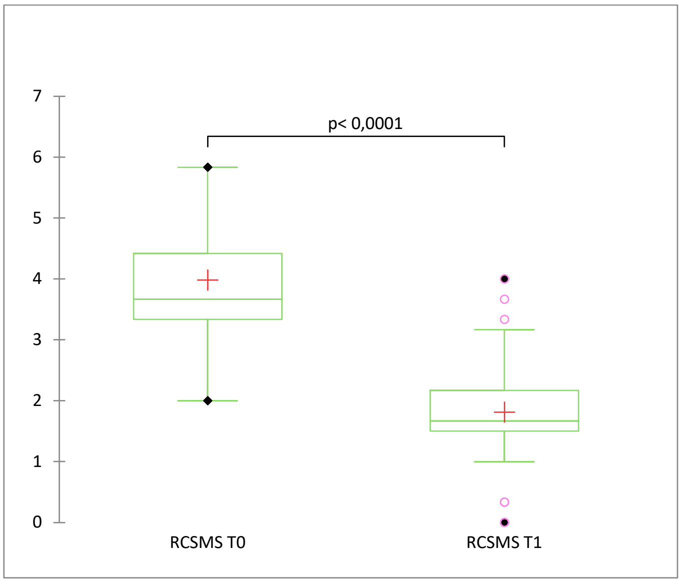

Figure S43. Box plot of the RCSMS at baseline (T0) and after one year of allergen immunotherapy (T1) in pediatric patients.

Boxes represent the interquartile range (IQR), the central horizontal line indicates the median, and whiskers denote minimum and maximum values. Red crosses indicate mean values, and pink symbols represent outliers. Pairwise comparisons between time points are shown with corresponding p-values. *Statistically significant differences compared with baseline (α = 0.05). RCSMS: Rhinoconjunctivitis Symptom and Medication Score.

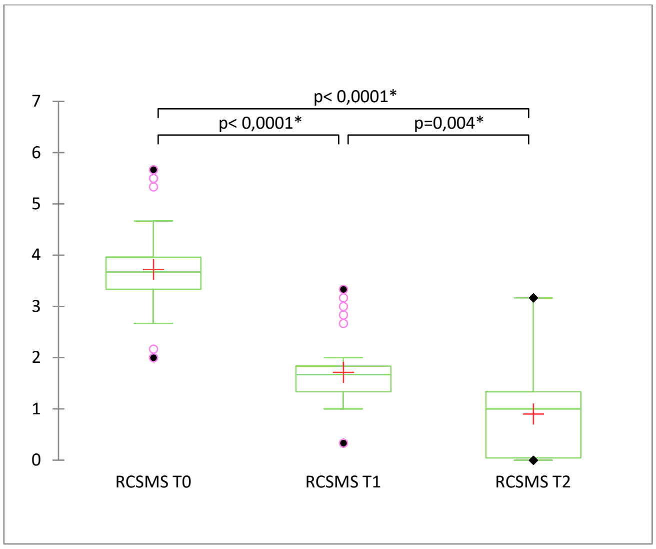

Figure S44. Box plot of the RCSMS at baseline, and after one and two years of immunotherapy in pediatric patients with complete two-year AIT data.

Boxes represent the interquartile range (IQR), the central horizontal line indicates the median, and whiskers denote minimum and maximum values. Red crosses indicate mean values, and pink symbols represent outliers. Pairwise comparisons between time points are shown with corresponding p-values. *Statistically significant differences compared with baseline (α = 0.05). RCSMS: Rhinoconjunctivitis Symptom and Medication Score.

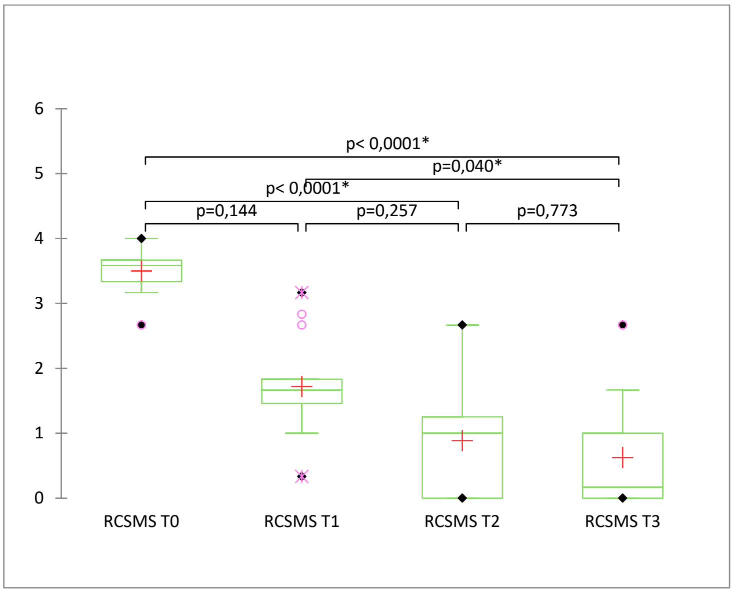

Figure S45. Box plot of the RCSMS at baseline, and after one, two, and three years of immunotherapy in pediatric patients with complete three-year AIT data.

Boxes represent the interquartile range (IQR), the central horizontal line indicates the median, and whiskers denote minimum and maximum values. Red crosses indicate mean values, and pink symbols represent outliers. Pairwise comparisons between time points are shown with corresponding p-values. *Statistically significant differences compared with baseline (α = 0.05). RCSMS: Rhinoconjunctivitis Symptom and Medication Score.

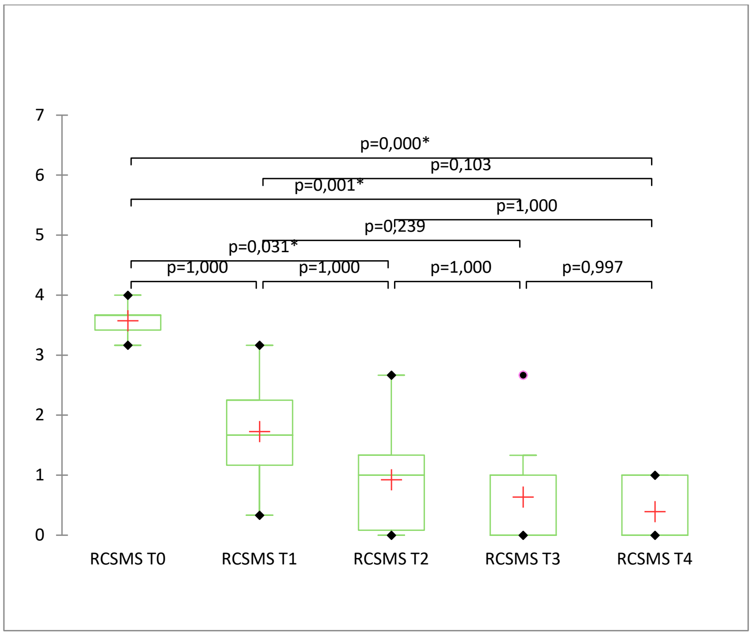

Figure S46. Box plot of the RCSMS at baseline, and after one, two, three and four years of immunotherapy in pediatric patients with complete four-year AIT data.

Boxes represent the interquartile range (IQR), the central horizontal line indicates the median, and whiskers denote minimum and maximum values. Red crosses indicate mean values, and pink symbols represent outliers. Pairwise comparisons between time points are shown with corresponding p-values. *Statistically significant differences compared with baseline (α = 0.05). RCSMS: Rhinoconjunctivitis Symptom and Medication Score.

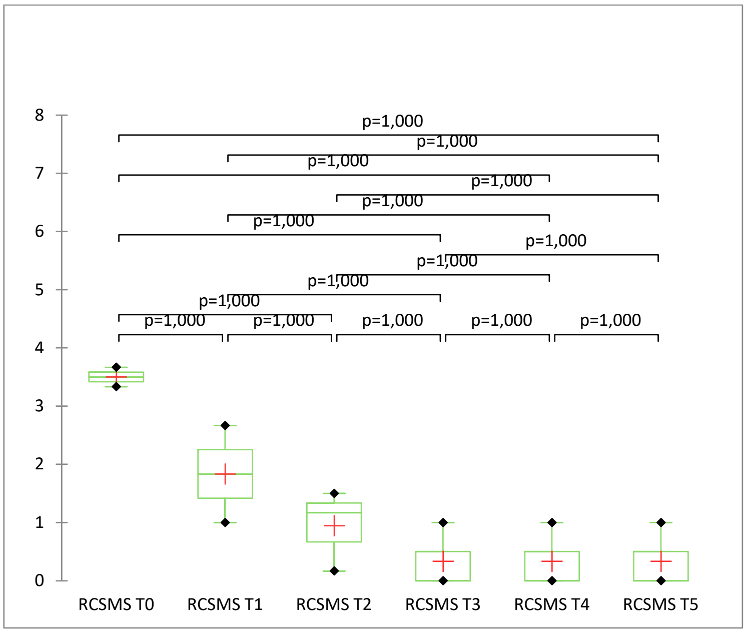

Figure S47. Box plot of the RCSMS at baseline, and after one, two, three, four and five years of immunotherapy in pediatric patients with complete five-year AIT data.

Boxes represent the interquartile range (IQR), the central horizontal line indicates the median, and whiskers denote minimum and maximum values. Red crosses indicate mean values, and pink symbols represent outliers. Pairwise comparisons between time points are shown with corresponding p-values. *Statistically significant differences compared with baseline (α = 0.05). RCSMS: Rhinoconjunctivitis Symptom and Medication Score.

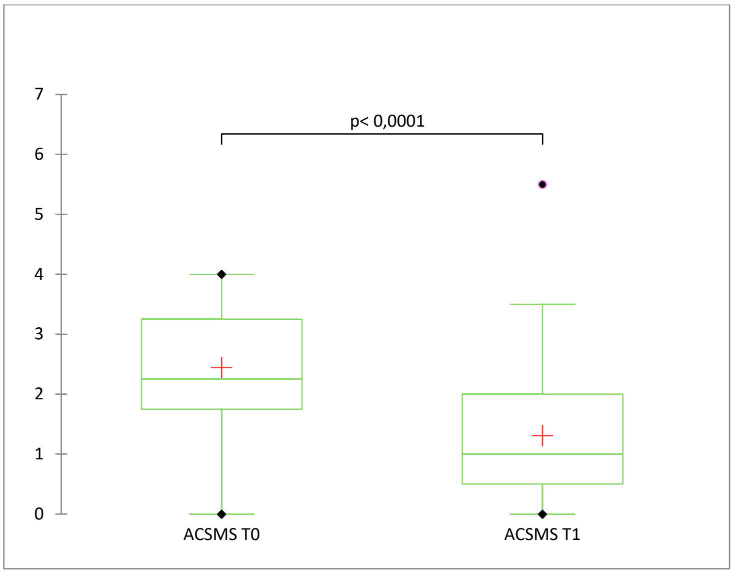

Figure S48. Box plot of the ACSMS at baseline (T0) and after one year of allergen immunotherapy (T1) in pediatric patients.

Boxes represent the interquartile range (IQR), the central horizontal line indicates the median, and whiskers denote minimum and maximum values. Red crosses indicate mean values, and pink symbols represent outliers. Pairwise comparisons between time points are shown with corresponding p-values. *Statistically significant differences compared with baseline (α = 0.05). ACSMS: Asthma Symptom and Medication Score.

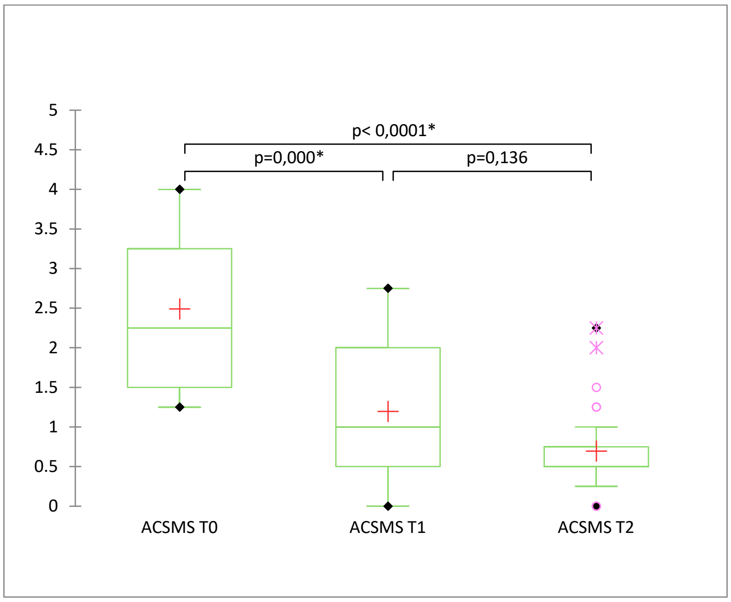

Figure S49. Box plot of the ACSMS at baseline, and after one and two years of immunotherapy in pediatric patients with complete two-year AIT data.

Boxes represent the interquartile range (IQR), the central horizontal line indicates the median, and whiskers denote minimum and maximum values. Red crosses indicate mean values, and pink symbols represent outliers. Pairwise comparisons between time points are shown with corresponding p-values. *Statistically significant differences compared with baseline (α = 0.05). ACSMS: Asthma Symptom and Medication Score.

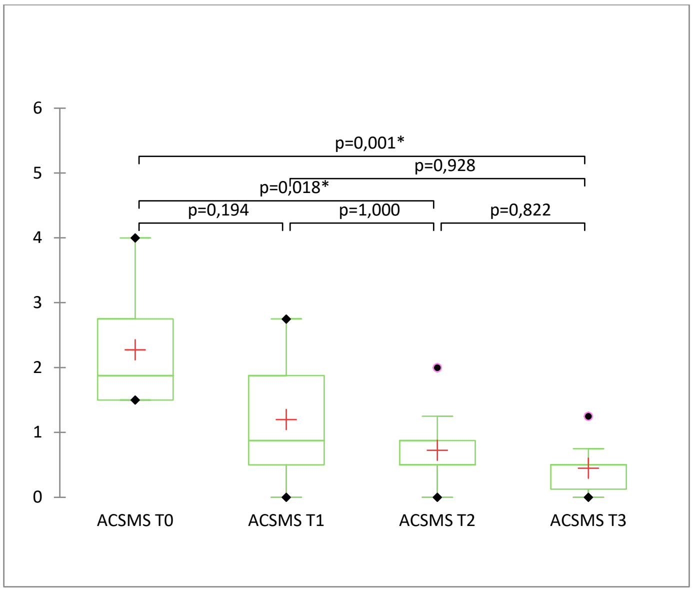

Figure S50. Box plot of the ACSMS at baseline, and after one, two, and three years of immunotherapy in pediatric patients with complete three-year AIT data.

Boxes represent the interquartile range (IQR), the central horizontal line indicates the median, and whiskers denote minimum and maximum values. Red crosses indicate mean values, and pink symbols represent outliers. Pairwise comparisons between time points are shown with corresponding p-values. *Statistically significant differences compared with baseline (α = 0.05). ACSMS: Asthma Symptom and Medication Score.

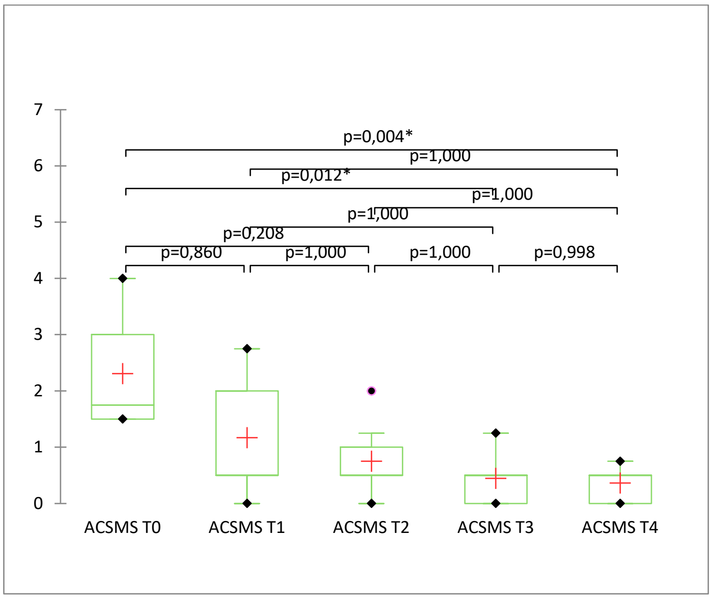

Figure S51. Box plot of the ACSMS at baseline, and after one, two, three and four years of immunotherapy in pediatric patients with complete four-year AIT data.

Boxes represent the interquartile range (IQR), the central horizontal line indicates the median, and whiskers denote minimum and maximum values. Red crosses indicate mean values, and pink symbols represent outliers. Pairwise comparisons between time points are shown with corresponding p-values. *Statistically significant differences compared with baseline (α = 0.05). ACSMS: Asthma Symptom and Medication Score.

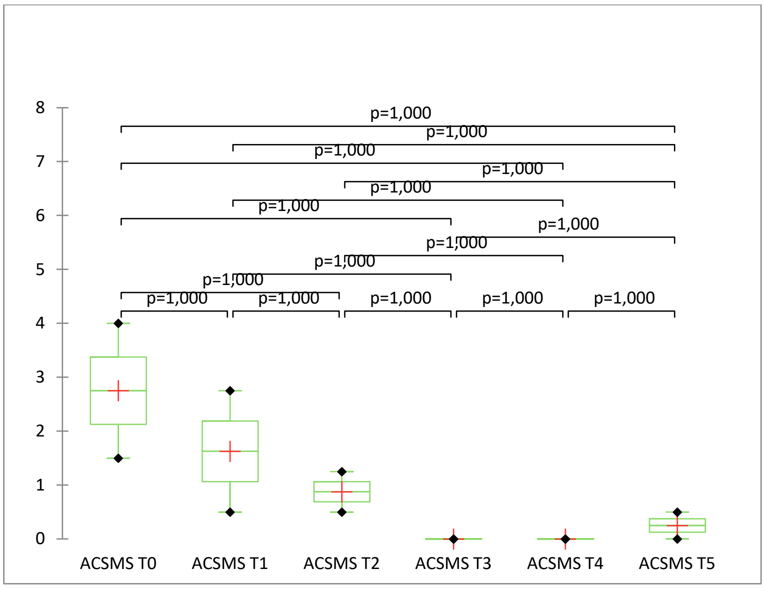

Figure S52. Box plot of the ACSMS at baseline, and after one, two, three, four and five years of immunotherapy in pediatric patients with complete five-year AIT data.

Boxes represent the interquartile range (IQR), the central horizontal line indicates the median, and whiskers denote minimum and maximum values. Red crosses indicate mean values, and pink symbols represent outliers. Pairwise comparisons between time points are shown with corresponding p-values. *Statistically significant differences compared with baseline (α = 0.05). ACSMS: Asthma Symptom and Medication Score.

Table S4. Distribution of symptom and medication severity categories over the study period.

	T0	T1	T2	T3	T4	T5
			RSS Classification, n (%)			
**No symptoms**	0 (0)	7 (3.5)	34 (32.1)	33 (54.1)	23 (57.5)	8 (72.7)
**Mild**	10 (5)	149 (74.9)	64 (60.4)	25 (41)	15 (37.5)	3 (27.3)
**Moderate**	121 (60.8)	39 (19.6)	6 (5.7)	3 (4.9)	2 (5)	0 (0)
**Severe**	68 (34.2)	4 (2)	2 (1.9)	0 (0)	0 (0)	0 (0)
**n**	199	199	106	61	40	11
			ASS Classification, n (%)			
**No symptoms**	2 (1.6)	29 (23.2)	29 (41.4)	26 (66.7)	21 (75.0)	5 (71.4)
**Mild**	56 (44.8)	88 (70.4)	38 (54.3)	12 (30.8)	7 (25.0)	2 (28.6)
**Moderate**	65 (52.0)	7 (5.6)	3 (4.3)	1 (2.6)	0 (0.0)	0 (0.0)
**Severe**	2 (1.6)	1 (0.8)	0 (0.0)	0 (0.0)	0 (0.0)	0 (0.0)
**n**	125	125	72	39	28	7
**Score**			RMS Classification, n (%)			
**0**	0 (0.0)	13 (6.5)	28 (26.4)	23 (37.7)	17 (42.5)	7 (63.6)
**1**	29 (14.6)	147 (73.9)	67 (63.2)	32 (52.5)	20 (50.0)	3 (27.3)
**2**	140 (70.4)	39 (19.6)	11 (10.4)	6 (9.8)	3 (7.5)	1 (9.1)
**3**	30 (15.1)	0 (0.0)	0 (0.0)	0 (0.0)	0 (0.0)	0 (0.0)
**n**	199	199	106	61	40	11
**Score**			AMS Classification, n (%)			
**0**	2 (1.6)	25 (20.0)	16 (22.9)	11 (28.2)	9 (32.1)	2 (28.6)
**0.5**	30 (24.0)	41 (32.8)	31 (44.3)	22 (56.4)	15 (53.6)	4 (57.1)
**1**	26 (20.8)	22 (17.6)	3 (4.3)	2 (5.1)	2 (7.1)	0 (0.0)
**1.5**	26 (20.8)	18 (14.4)	17 (24.3)	4 (10.3)	2 (7.1)	1 (14.3)
**2**	32 (25.6)	16 (12.8)	3 (4.3)	0 (0.0)	0 (0.0)	0 (0.0)
**2.5**	8 (6.4)	3 (2.4)	0 (0.0)	0 (0.0)	0 (0.0)	0 (0.0)
**3**	1 (0.8)	0 (0.0)	0 (0.0)	0 (0.0)	0 (0.0)	0 (0.0)
**n**	125	125	70	39	28	7

RSS: Rhinoconjunctivitis Symptom Score. ASS: Asthma Symptom Score. RMS: Rhinoconjunctivitis Medication Score. AMS: Asthma Medication Score.

Table S5. Distribution of symptom and medication severity categories over the study period in pediatric patients.

	T0	T1	T2	T3	T4	T5
			**RSS Classification, n (%)**			
**No symptoms**	0 (0.0)	5 (6.6)	15 (44.1)	12 (66.7)	11 (73.3)	3 (100)
**Mild**	5 (6.6)	56 (73.7)	18 (52.9)	6 (33.3)	4 (26.7)	0 (0.0)
**Moderate**	42 (55.3)	15 (19.7)	1 (2.9)	0 (0.0)	0 (0.0)	0 (0.0)
**Severe**	29 (38.2)	0 (0.0)	0 (0.0)	0 (0.0)	0 (0.0)	0 (0.0)
**n**	76	76	34	18	15	3
			**ASS Classification, n (%)**			
**No symptoms**	1 (2.0)	15 (30.0)	13 (56.5)	10 (83.3)	10 (83.3)	2 (100.0)
**Mild**	22 (44.0)	31 (62.0)	10 (43.5)	2 (16.7)	2 (16.7)	0 (0.0)
**Moderate**	26 (52.0)	3 (6.0)	0 (0.0)	0 (0.0)	0 (0.0)	0 (0.0)
**Severe**	1 (2.0)	1 (2.0)	0 (0.0)	0 (0.0)	0 (0.0)	0 (0.0)
**n**	50	50	23	12	12	2
**Score**			**RMS Classification, n (%)**			
**0**	0 (0.0)	4 (5.3)	15 (44.1)	11 (61.1)	11 (73.3)	2 (66.7)
**1**	7 (9.2)	62 (81.6)	16 (47.1)	6 (33.3)	4 (26.7)	1 (33.3)
**2**	54 (71.1)	10 (13.2)	3 (8.8)	1 (5.6)	0 (0.0)	0 (0.0)
**3**	15 (19.7)	0 (0.0)	0 (0.0)	0 (0.0)	0 (0.0)	0 (0.0)
**n**	76	76	34	18	15	3
**Score**			**AMS Classification, n (%)**			
**0**	1 (2.0)	9 (18.0)	6 (26.1)	5 (41.7)	6 (50.0)	1 (50.0)
**0.5**	13 (26.0)	20 (40.0)	13 (56.5)	6 (50.0)	6 (50.0)	1 (50.0)
**1**	16 (32.0)	10 (20.0)	2 (8.7)	1 (8.3)	0 (0.0)	0 (0.0)
**1.5**	8 (16.0)	7 (14.0)	2 (8.7)	0 (0.0)	0 (0.0)	0 (0.0)
**2**	10 (20.0)	3 (6.0)	0 (0.0)	0 (0.0)	0 (0.0)	0 (0.0)
**2.5**	1 (2.0)	1 (2.0)	0 (0.0)	0 (0.0)	0 (0.0)	0 (0.0)
**3**	1 (2.0)	0 (0.0)	0 (0.0)	0 (0.0)	0 (0.0)	0 (0.0)
**n**	50	50	23	12	12	2

RSS: Rhinoconjunctivitis Symptom Score. ASS: Asthma Symptom Score. RMS: Rhinoconjunctivitis Medication Score. AMS: Asthma Medication Score.

Table S6. Descriptive statistics of the RCSMS at baseline (T0) and after one year of allergen immunotherapy (T1).

	RCSMS	
	T0	T1
**Mean**	3.85	1.92
**95% lower CI**	3.72	1.81
**95% upper CI**	3.97	2.04
**SD**	0.89	0.83
**Median**	3.67	1.67
**Q1**	3.33	1.5
**Q3**	4.33	2.33
**Maximum**	5.83	4.33
**Minimum**	1.5	0
**n**	199	199
**Reduction vs. T0 (median)**		54.50%

CI: Confidence Interval. SD: Standard Deviation. Q1: First quartile. Q3: Third quartile. RCSMS: Rhinoconjunctivitis Combined Symptom and Medication Score.

Table S7. Descriptive statistics of the RCSMS in the subset of patients who completed two years of AIT.

	RCSMS		
	T0	T1	T2
**Mean**	3.63	1.94	1.25
**95% lower CI**	3.48	1.78	1.08
**95% upper CI**	3.77	2.09	1.43
**SD**	0.74	0.8	0.93
**Median**	3.67	1.67	1.33
**Q1**	3.33	1.5	0.5
**Q3**	3.83	2.46	1.67
**Maximum**	5.67	4.33	4.67
**Minimum**	2	0	0
**n**	106	106	106
**Reduction vs. T0 (median)**		54.50%	63.76%

CI: Confidence Interval. SD: Standard Deviation. Q1: First quartile. Q3: Third quartile. RCSMS: Rhinoconjunctivitis Combined Symptom and Medication Score.

Table S8. Descriptive statistics of the RCSMS in the subset of patients who completed three years of AIT.

	RCSMS			
	T0	T1	T2	T3
**Mean**	3.56	1.97	1.37	0.98
**95% lower CI**	3.43	1.78	1.13	0.75
**95% upper CI**	3.7	2.16	1.62	1.22
**SD**	0.52	0.74	0.91	0.94
**Median**	3.58	1.67	1.33	1
**Q1**	3.33	1.67	1	0
**Q3**	3.67	2.63	1.67	1.63
**Maximum**	5.5	3.83	4.17	3.83
**Minimum**	2.33	0	0	0
**n**	62	62	57	62
**Reduction vs. T0 (median)**		53.42%	62.90%	72.11%

CI: Confidence Interval. SD: Standard Deviation. Q1: First quartile. Q3: Third quartile. RCSMS: Rhinoconjunctivitis Combined Symptom and Medication Score.

Table S9. Descriptive statistics of the RCSMS in the subset of patients who completed four years of AIT.

	RCSMS				
	T0	T1	T2	T3	T4
**Mean**	3.57	2.03	1.41	1.12	0.86
**95% lower CI**	3.4	1.77	1.1	0.79	0.58
**95% upper CI**	3.74	2.28	1.73	1.45	1.14
**SD**	0.52	0.81	0.93	0.97	0.88
**Median**	3.5	1.75	1.33	1	1
**Q1**	3.33	1.67	1	0	0
**Q3**	3.71	2.67	1.71	1.67	1.33
**Maximum**	5	3.83	4.17	3.83	3.33
**Minimum**	2.33	0	0	0	0
**n**	40	40	36	36	40
**Reduction vs. T0 (median)**		50.00%	62.00%	71.43%	71.43%

CI: Confidence Interval. SD: Standard Deviation. Q1: First quartile. Q3: Third quartile. RCSMS: Rhinoconjunctivitis Combined Symptom and Medication Score.

Table S10. Descriptive statistics of the RCSMS in the subset of patients who completed five years of AIT.

	RCSMS					
	T0	T1	T2	T3	T4	T5
**Mean**	3.74	2.47	1.33	1.05	0.89	0.56
**95% lower CI**	3.33	1.95	0.74	0.35	0.21	0.01
**95% upper CI**	4.15	2.99	1.92	1.74	1.58	1.12
**SD**	0.61	0.78	0.88	1.04	1.02	0.82
**Median**	3.5	2.67	1.17	1	1	0.17
**Q1**	3.33	2.08	1	0	0	0
**Q3**	3.75	2.84	1.66	1.67	1.25	1
**Maximum**	5	3.83	3.33	3.33	3.33	2.67
**Minimum**	3.17	1	0.17	0	0	0
**n**	11	11	11	11	11	11
**Reduction vs. T0 (median)**		23.71%	66.57%	71.43%	71.43%	95.14%

CI: Confidence Interval. SD: Standard Deviation. Q1: First quartile. Q3: Third quartile. RCSMS: Rhinoconjunctivitis Combined Symptom and Medication Score.

Table S11. Descriptive statistics of the ACSMS at baseline (T0) and after one year of allergen immunotherapy (T1).

	ACSMS	
	T0	T1
**Mean**	2.6	1.44
**95% lower CI**	2.42	1.25
**95% upper CI**	2.78	1.64
**SD**	1.02	1.09
**Median**	2.5	1.25
**Q1**	1.75	0.5
**Q3**	3.5	2.5
**Maximum**	4.5	5.5
**Minimum**	0	0
**n**	125	125
**Reduction vs. T0 (median)**		50.00%

CI: Confidence Interval. SD: Standard Deviation. Q1: First quartile. Q3: Third quartile. ACSMS: Asthma Combined Symptom and Medication Score.

Table S12. Descriptive statistics of the ACSMS in the subset of patients who completed two years of AIT.

	ACSMS		
	T0	T1	T2
**Mean**	2.68	1.45	1.08
**95% lower CI**	2.43	1.21	0.85
**95% upper CI**	2.93	1.7	1.31
**SD**	1.06	1.04	0.96
**Median**	2.88	1.25	0.62
**Q1**	1.5	0.5	0.5
**Q3**	3.5	2.5	2
**Maximum**	4.5	3.75	3.5
**Minimum**	0.5	0	0
**n**	70	70	70
**Reduction vs. T0 (median)**		56.52%	78.26%

CI: Confidence Interval. SD: Standard Deviation. Q1: First quartile. Q3: Third quartile. ACSMS: Asthma Combined Symptom and Medication Score.

Table S13. Descriptive statistics of the ACSMS in the subset of patients who completed three years of AIT.

	ACSMS			
	T0	T1	T2	T3
**Mean**	2.61	1.53	1.2	0.67
**95% lower CI**	2.23	1.2	0.87	0.44
**95% upper CI**	2.99	1.85	1.53	0.91
**SD**	1.18	1.01	0.97	0.72
**Median**	2.5	1.5	0.75	0.5
**Q1**	1.5	0.75	0.5	0
**Q3**	3.75	2.5	2.12	0.75
**Maximum**	4.5	3.5	3.25	2.75
**Minimum**	0.5	0	0	0
**n**	39	39	36	39
**Reduction vs. T0 (median)**		40.00%	70.00%	80.00%

CI: Confidence Interval. SD: Standard Deviation. Q1: First quartile. Q3: Third quartile. ACSMS: Asthma Combined Symptom and Medication Score.

Table S14. Descriptive statistics of the ACSMS in the subset of patients who completed four years of AIT.

	ACSMS				
	T0	T1	T2	T3	T4
**Mean**	2.42	1.33	1.05	0.75	0.56
**95% lower CI**	1.96	0.96	0.68	0.44	0.32
**95% upper CI**	2.88	1.7	1.42	1.06	0.8
**SD**	1.18	0.94	0.91	0.75	0.62
**Median**	2	1.38	0.75	0.5	0.5
**Q1**	1.5	0.5	0.5	0.5	0
**Q3**	3.31	2	1.5	1	0.56
**Maximum**	4.5	3	3.25	2.75	2.5
**Minimum**	0.5	0	0	0	0
**n**	28	28	25	25	28
**Reduction vs. T0 (median)**		31.25%	62.50%	75.00%	75.00%

CI: Confidence Interval. SD: Standard Deviation. Q1: First quartile. Q3: Third quartile. ACSMS: Asthma Combined Symptom and Medication Score.

Table S15. Descriptive statistics of the ACSMS in the subset of patients who completed five years of AIT.

	ACSMS					
	T0	T1	T2	T3	T4	T5
**Mean**	1.93	1	0.57	0.75	0.57	0.61
**95% lower CI**	0.87	0.08	0.03	-0.13	-0.17	-0.02
**95% upper CI**	2.99	1.92	1.12	1.63	1.31	1.23
**SD**	1.14	0.99	0.59	0.95	0.8	0.67
**Median**	1.5	0.75	0.5	0.5	0.5	0.5
**Q1**	1.38	0.25	0.12	0	0	0.25
**Q3**	2.25	1.5	0.88	1.12	0.62	0.62
**Maximum**	4	2.75	1.5	2.5	2.25	2
**Minimum**	0.75	0	0	0	0	0
**n**	7	7	7	7	7	7
**Reduction vs. T0 (median)**		50.00%	66.67%	66.67%	66.67%	66.67%

CI: Confidence Interval. SD: Standard Deviation. Q1: First quartile. Q3: Third quartile. ACSMS: Asthma Combined Symptom and Medication Score.

Table S16. Descriptive statistics of the RSS at baseline (T0) and after one year of allergen immunotherapy (T1).

	RSS	
	**T0**	**T1**
**Mean**	1.84	0.79
**95% lower CI**	1.77	0.72
**95% upper CI**	1.92	0.86
**SD**	0.54	0.49
**Median**	1.83	0.67
**Q1**	1.5	0.5
**Q3**	2.33	1
**Maximum**	3	3
**Minimum**	0.5	0
**n**	199	199
**Reduction vs. T0 (median)**		63.39%

CI: Confidence Interval. SD: Standard Deviation. Q1: First quartile. Q3: Third quartile. RSS: Rhinoconjunctivitis Symptom Score.

Table S17. Descriptive statistics of the RSS in the subset of patients who completed two years of AIT.

	RSS		
	**T0**	**T1**	**T2**
**Mean**	1.71	0.77	0.41
**95% lower CI**	1.62	0.68	0.33
**95% upper CI**	1.8	0.85	0.5
**SD**	0.47	0.46	0.46
**Median**	1.67	0.67	0.33
**Q1**	1.33	0.5	0
**Q3**	2	1	0.67
**Maximum**	3	2.83	2.67
**Minimum**	0.67	0	0
**n**	106	106	106
**Reduction vs. T0 (median)**		59.88%	80.24%

CI: Confidence Interval. SD: Standard Deviation. Q1: First quartile. Q3: Third quartile. RSS: Rhinoconjunctivitis Symptom Score.

Table S18. Descriptive statistics of the RSS in the subset of patients who completed three years of AIT.

	RSS			
	T0	T1	T2	T3
**Mean**	1.66	0.77	0.41	0.28
**95% lower CI**	1.54	0.66	0.29	0.18
**95% upper CI**	1.77	0.88	0.53	0.38
**SD**	0.45	0.43	0.46	0.4
**Median**	1.67	0.67	0.33	0
**Q1**	1.33	0.67	0	0
**Q3**	1.83	0.83	0.67	0.67
**Maximum**	3	2.83	2.17	1.83
**Minimum**	0.67	0	0	0
**n**	61	61	57	61
**Reduction vs. T0 (median)**		59.88%	80.24%	100.00%

CI: Confidence Interval. SD: Standard Deviation. Q1: First quartile. Q3: Third quartile. RSS:

Table S19. Descriptive statistics of the RSS in the subset of patients who completed four years of AIT.

	RSS				
	T0	T1	T2	T3	T4
**Mean**	1.67	0.8	0.44	0.32	0.21
**95% lower CI**	1.52	0.64	0.28	0.17	0.1
**95% upper CI**	1.82	0.96	0.6	0.46	0.32
**SD**	0.47	0.49	0.47	0.43	0.34
**Median**	1.58	0.67	0.33	0	0
**Q1**	1.33	0.67	0	0	0
**Q3**	1.71	1	0.67	0.67	0.33
**Maximum**	3	2.83	2.17	1.83	1.33
**Minimum**	1.17	0	0	0	0
**n**	40	40	36	36	40
**Reduction vs. T0 (median)**		57.73%	79.18%	100.00%	100.00%

CI: Confidence Interval. SD: Standard Deviation. Q1: First quartile. Q3: Third quartile. RSS: Rhinoconjunctivitis Symptom Score.

Table S20. Descriptive statistics of the RSS in the subset of patients who completed five years of AIT.

	RSS					
	**T0**	**T1**	**T2**	**T3**	**T4**	**T5**
**Mean**	1.83	0.92	0.42	0.32	0.26	0.11
**95% lower CI**	1.37	0.44	0.13	0.02	-0.02	-0.04
**95% upper CI**	2.3	1.4	0.72	0.62	0.54	0.25
**SD**	0.69	0.71	0.44	0.45	0.42	0.22
**Median**	1.5	0.67	0.17	0	0	0
**Q1**	1.33	0.67	0.17	0	0	0
**Q3**	2.25	1	0.66	0.67	0.33	0.08
**Maximum**	3	2.83	1.33	1.33	1.33	0.67
**Minimum**	1.17	0	0	0	0	0
**n**	11	11	11	11	11	11
**Reduction vs. T0 (median)**		55.33%	88.67%	100.00%	100.00%	100.00%

CI: Confidence Interval. SD: Standard Deviation. Q1: First quartile. Q3: Third quartile. RSS: Rhinoconjunctivitis Symptom Score.

Table S21. Descriptive statistics of the ASS at baseline (T0) and after one year of allergen immunotherapy (T1).

	ASS	
	**T0**	**T1**
**Mean**	1.27	0.57
**95% lower CI**	1.18	0.48
**95% upper CI**	1.35	0.66
**SD**	0.48	0.51
**Median**	1.25	0.5
**Q1**	1	0.25
**Q3**	1.75	1
**Maximum**	2.25	3
**Minimum**	0	0
**n**	125	125
**Reduction vs. T0 (median)**		60.00%

CI: Confidence Interval. SD: Standard Deviation. Q1: First quartile. Q3: Third quartile. ASS: Asthma Symptom Score.

Table S22. Descriptive statistics of the ASS in the subset of patients who completed two years of AIT.

	ASS		
	T0	T1	T2
**Mean**	1.33	0.55	0.37
**95% lower CI**	1.22	0.44	0.26
**95% upper CI**	1.44	0.66	0.47
**SD**	0.47	0.46	0.45
**Median**	1.25	0.5	0.25
**Q1**	1	0	0
**Q3**	1.75	1	0.5
**Maximum**	2.25	2	2
**Minimum**	0	0	0
**n**	70	70	70
**Reduction vs. T0 (median)**		60.00%	80.00%

CI: Confidence Interval. SD: Standard Deviation. Q1: First quartile. Q3: Third quartile. ASS: Asthma Symptom Score.

Table S23. Descriptive statistics of the ASS in the subset of patients who completed three years of AIT.

	ASS			
	T0	T1	T2	T3
**Mean**	1.3	0.59	0.38	0.19
**95% lower CI**	1.12	0.43	0.23	0.08
**95% upper CI**	1.48	0.75	0.54	0.3
**SD**	0.55	0.49	0.46	0.34
**Median**	1	0.5	0.25	0
**Q1**	1	0	0	0
**Q3**	2	1	0.5	0.25
**Maximum**	2.25	2	1.75	1.25
**Minimum**	0	0	0	0
**n**	39	39	36	39
**Reduction vs. T0 (median)**		50.00%	75.00%	100.00%

CI: Confidence Interval. SD: Standard Deviation. Q1: First quartile. Q3: Third quartile. ASS: Asthma Symptom Score.

Table S24. Descriptive statistics of the ASS in the subset of patients who completed four years of AIT.

	ASS				
	T0	T1	T2	T3	T4
**Mean**	1.21	0.46	0.33	0.21	0.12
**95% lower CI**	1.01	0.29	0.14	0.06	0.02
**95% upper CI**	1.4	0.62	0.52	0.36	0.21
**SD**	0.5	0.43	0.45	0.37	0.25
**Median**	1	0.5	0.25	0	0
**Q1**	1	0	0	0	0
**Q3**	1.56	1	0.5	0.25	0.06
**Maximum**	2	1	1.75	1.25	1
**Minimum**	0	0	0	0	0
**n**	28	28	25	25	28
**Reduction vs. T0 (median)**		50.00%	75.00%	100.00%	100.00%

CI: Confidence Interval. SD: Standard Deviation. Q1: First quartile. Q3: Third quartile. ASS: Asthma Symptom Score.

Table S25. Descriptive statistics of the ASS in the subset of patients who completed five years of AIT.

	ASS					
	T0	T1	T2	T3	T4	T5
**Mean**	1.14	0.5	0.21	0.32	0.14	0.11
**95% lower CI**	0.72	0.06	-0.12	-0.12	-0.12	-0.07
**95% upper CI**	1.56	0.94	0.55	0.76	0.41	0.29
**SD**	0.45	0.48	0.37	0.47	0.28	0.2
**Median**	1	0.75	0	0	0	0
**Q1**	0.88	0	0	0	0	0
**Q3**	1.25	0.88	0.25	0.62	0.12	0.12
**Maximum**	2	1	1	1	0.75	0.5
**Minimum**	0.75	0	0	0	0	0
**n**	7	7	7	7	7	7
**Reduction vs. T0 (median)**		25.00%	100.00%	100.00%	100.00%	100.00%

CI: Confidence Interval. SD: Standard Deviation. Q1: First quartile. Q3: Third quartile. ASS: Asthma Symptom Score.

Table S26. Descriptive statistics of the RCSMS at baseline (T0) and after one year of allergen immunotherapy (T1) in pediatric patients.

	RCSMS	
	T0	T1
**Mean**	3.98	1.81
**95% lower CI**	3.78	1.65
**95% upper CI**	4.18	1.98
**SD**	0.88	0.72
**Median**	3.67	1.67
**Q1**	3.33	1.5
**Q3**	4.42	2.17
**Maximum**	5.83	4
**Minimum**	2	0
**n**	76	76
**Reduction vs. T0 (median)**		54.50%

CI: Confidence Interval. SD: Standard Deviation. Q1: First quartile. Q3: Third quartile. RCSMS: Rhinoconjunctivitis Combined Symptom and Medication Score.

Table S27. Descriptive statistics of the RCSMS in the subset of pediatric patients who completed two years of AIT.

	RCSMS		
	T0	T1	T2
**Mean**	3.72	1.72	0.9
**95% lower CI**	3.43	1.49	0.61
**95% upper CI**	4.01	1.94	1.2
**SD**	0.84	0.64	0.84
**Median**	3.67	1.67	1
**Q1**	3.33	1.33	0.04
**Q3**	3.96	1.83	1.33
**Maximum**	5.67	3.33	3.17
**Minimum**	2	0.33	0
**n**	34	34	34
**Reduction vs. T0 (median)**		54.50%	72.75%

CI: Confidence Interval. SD: Standard Deviation. Q1: First quartile. Q3: Third quartile. RCSMS: Rhinoconjunctivitis Combined Symptom and Medication Score.

Table S28. Descriptive statistics of the RCSMS in the subset of pediatric patients who completed three years of AIT.

	RCSMS			
	**T0**	**T1**	**T2**	**T3**
**Mean**	3.6	1.77	0.89	0.54
**95% lower CI**	3.42	1.45	0.41	0.18
**95% upper CI**	3.78	2.09	1.36	0.91
**SD**	0.38	0.67	0.9	0.75
**Median**	3.67	1.67	1	0.17
**Q1**	3.42	1.5	0	0
**Q3**	3.75	2	1.25	1
**Maximum**	4.33	3.17	2.67	2.67
**Minimum**	2.67	0.33	0	0
**n**	19	19	16	19
**Reduction vs. T0 (median)**		54.50%	72.75%	95.37%

CI: Confidence Interval. SD: Standard Deviation. Q1: First quartile. Q3: Third quartile. RCSMS: Rhinoconjunctivitis Combined Symptom and Medication Score.

Table S29. Descriptive statistics of the RCSMS in the subset of pediatric patients who completed four years of AIT.

	RCSMS				
	**T0**	**T1**	**T2**	**T3**	**T4**
**Mean**	3.68	1.78	0.93	0.64	0.32
**95% lower CI**	3.46	1.36	0.36	0.06	0.08
**95% upper CI**	3.9	2.2	1.49	1.22	0.56
**SD**	0.4	0.76	0.84	0.86	0.43
**Median**	3.67	1.67	1	0	0.17
**Q1**	3.5	1.42	0.08	0	0
**Q3**	3.75	2.25	1.34	1	0.66
**Maximum**	4.83	3.17	2.67	2.67	1
**Minimum**	3.17	0.33	0	0	0
**n**	15	15	11	11	15
**Reduction vs. T0 (median)**		54.50%	72.75%	100.00%	95.37%

CI: Confidence Interval. SD: Standard Deviation. Q1: First quartile. Q3: Third quartile. RCSMS: Rhinoconjunctivitis Combined Symptom and Medication Score.

Table S30. Descriptive statistics of the RCSMS in the subset of pediatric patients who completed five years of AIT.

	RCSMS					
	T0	T1	T2	T3	T4	T5
**Mean**	3.5	1.83	0.95	0.33	0.33	0.33
**95% lower CI**	3.08	-0.24	-0.77	-1.1	-1.1	-1.1
**95% upper CI**	3.92	3.91	2.67	1.77	1.77	1.77
**SD**	0.17	0.84	0.69	0.58	0.58	0.58
**Median**	3.5	1.83	1.17	0	0	0
**Q1**	3.42	1.42	0.67	0	0	0
**Q3**	3.58	2.25	1.34	0.5	0.5	0.5
**Maximum**	3.67	2.67	1.5	1	1	1
**Minimum**	3.33	1	0.17	0	0	0
**n**	3	3	3	3	3	3
**Reduction vs. T0 (median)**		47.71%	66.57%	100.00%	100.00%	100.00%

CI: Confidence Interval. SD: Standard Deviation. Q1: First quartile. Q3: Third quartile. RCSMS: Rhinoconjunctivitis Combined Symptom and Medication Score.

Table S31. Descriptive statistics of the ACSMS at baseline (T0) and after one year of allergen immunotherapy (T1) in pediatric patients.

	ACSMS	
	T0	T1
**Mean**	2.6	1.44
**95% lower CI**	2.42	1.25
**95% upper CI**	2.78	1.64
**SD**	1.02	1.09
**Median**	2.5	1.25
**Q1**	1.75	0.5
**Q3**	3.5	2.5
**Maximum**	4.5	5.5
**Minimum**	0	0
**n**	125	125
**Reduction vs. T0 (median)**		50.00%

CI: Confidence Interval. SD: Standard Deviation. Q1: First quartile. Q3: Third quartile. ACSMS: Asthma Combined Symptom and Medication Score.

Table S32. Descriptive statistics of the ACSMS in the subset of pediatric patients who completed two years of AIT.

	ACSMS		
	T0	T1	T2
**Mean**	2.68	1.45	1.08
**95% lower CI**	2.43	1.21	0.85
**95% upper CI**	2.93	1.7	1.31
**SD**	1.06	1.04	0.96
**Median**	2.88	1.25	0.62
**Q1**	1.5	0.5	0.5
**Q3**	3.5	2.5	2
**Maximum**	4.5	3.75	3.5
**Minimum**	0.5	0	0
**n**	70	70	70
**Reduction vs. T0 (median)**		56.52%	78.26%

CI: Confidence Interval. SD: Standard Deviation. Q1: First quartile. Q3: Third quartile. ACSMS: Asthma Combined Symptom and Medication Score.

Table S33. Descriptive statistics of the ACSMS in the subset of pediatric patients who completed three years of AIT.

	ACSMS			
	T0	T1	T2	T3
**Mean**	2.61	1.53	1.2	0.67
**95% lower CI**	2.23	1.2	0.87	0.44
**95% upper CI**	2.99	1.85	1.53	0.91
**SD**	1.18	1.01	0.97	0.72
**Median**	2.5	1.5	0.75	0.5
**Q1**	1.5	0.75	0.5	0
**Q3**	3.75	2.5	2.12	0.75
**Maximum**	4.5	3.5	3.25	2.75
**Minimum**	0.5	0	0	0
**n**	39	39	36	39
**Reduction vs. T0 (median)**		40.00%	70.00%	80.00%

CI: Confidence Interval. SD: Standard Deviation. Q1: First quartile. Q3: Third quartile. ACSMS: Asthma Combined Symptom and Medication Score.

Table S34. Descriptive statistics of the ACSMS in the subset of pediatric patients who completed four years of AIT.

	ACSMS				
	**T0**	**T1**	**T2**	**T3**	**T4**
**Mean**	2.42	1.33	1.05	0.75	0.56
**95% lower CI**	1.96	0.96	0.68	0.44	0.32
**95% upper CI**	2.88	1.7	1.42	1.06	0.8
**SD**	1.18	0.94	0.91	0.75	0.62
**Median**	2	1.38	0.75	0.5	0.5
**Q1**	1.5	0.5	0.5	0.5	0
**Q3**	3.31	2	1.5	1	0.56
**Maximum**	4.5	3	3.25	2.75	2.5
**Minimum**	0.5	0	0	0	0
**n**	28	28	25	25	28
**Reduction vs. T0 (median)**		31.25%	62.50%	75.00%	75.00%

CI: Confidence Interval. SD: Standard Deviation. Q1: First quartile. Q3: Third quartile. ACSMS: Asthma Combined Symptom and Medication Score.

Table S35. Descriptive statistics of the ACSMS in the subset of pediatric patients who completed five years of AIT.

	ACSMS					
	**T0**	**T1**	**T2**	**T3**	**T4**	**T5**
**Mean**	1.93	1	0.57	0.75	0.57	0.61
**95% lower CI**	0.87	0.08	0.03	-0.13	-0.17	-0.02
**95% upper CI**	2.99	1.92	1.12	1.63	1.31	1.23
**SD**	1.14	0.99	0.59	0.95	0.8	0.67
**Median**	1.5	0.75	0.5	0.5	0.5	0.5
**Q1**	1.38	0.25	0.12	0	0	0.25
**Q3**	2.25	1.5	0.88	1.12	0.62	0.62
**Maximum**	4	2.75	1.5	2.5	2.25	2
**Minimum**	0.75	0	0	0	0	0
**n**	7	7	7	7	7	7
**Reduction vs. T0 (median)**		50.00%	66.67%	66.67%	66.67%	66.67%

CI: Confidence Interval. SD: Standard Deviation. Q1: First quartile. Q3: Third quartile. ACSMS: Asthma Combined Symptom and Medication Score.

Table S36. Descriptive statistics of the RSS at baseline (T0) and after one year of allergen immunotherapy (T1) in pediatric patients.

	RSS	
	**T0**	**T1**
**Mean**	1.88	0.73
**95% lower CI**	1.76	0.63
**95% upper CI**	1.99	0.83
**SD**	0.52	0.43
**Median**	1.83	0.67
**Q1**	1.5	0.5
**Q3**	2.33	1
**Maximum**	3	2
**Minimum**	0.67	0
**n**	76	76
**Reduction vs. T0 (median)**		63.39%

CI: Confidence Interval. SD: Standard Deviation. Q1: First quartile. Q3: Third quartile. RSS: Rhinoconjunctivitis Symptom Score.

Table S37. Descriptive statistics of the RSS in the subset of pediatric patients who completed two years of AIT.

	RSS		
	**T0**	**T1**	**T2**
**Mean**	1.69	0.6	0.26
**95% lower CI**	1.53	0.48	0.15
**95% upper CI**	1.85	0.71	0.36
**SD**	0.46	0.33	0.29
**Median**	1.67	0.67	0.17
**Q1**	1.37	0.33	0
**Q3**	2	0.83	0.46
**Maximum**	2.67	1.33	1.17
**Minimum**	0.67	0	0
**n**	34	34	34
**Reduction vs. T0 (median)**		59.88%	89.82%

CI: Confidence Interval. SD: Standard Deviation. Q1: First quartile. Q3: Third quartile. RSS: Rhinoconjunctivitis Symptom Score.

Table S38. Descriptive statistics of the RSS in the subset of pediatric patients who completed three years of AIT.

	RSS			
	T0	T1	T2	T3
**Mean**	1.64	0.64	0.2	0.13
**95% lower CI**	1.44	0.47	0.06	0.02
**95% upper CI**	1.84	0.81	0.33	0.24
**SD**	0.39	0.34	0.25	0.23
**Median**	1.67	0.67	0.08	0
**Q1**	1.5	0.5	0	0
**Q3**	1.79	0.83	0.37	0.17
**Maximum**	2.33	1.33	0.67	0.67
**Minimum**	0.67	0	0	0
**n**	18	18	16	18
**Reduction vs. T0 (median)**		59.88%	94.91%	100.00%

CI: Confidence Interval. SD: Standard Deviation. Q1: First quartile. Q3: Third quartile. RSS: Rhinoconjunctivitis Symptom Score.

Table S39. Descriptive statistics of the RSS in the subset of pediatric patients who completed four years of AIT.

	RSS				
	T0	T1	T2	T3	T4
**Mean**	1.68	0.64	0.2	0.09	0.06
**95% lower CI**	1.46	0.43	0.03	-0.05	0
**95% upper CI**	1.9	0.86	0.36	0.24	0.11
**SD**	0.4	0.39	0.25	0.22	0.1
**Median**	1.67	0.67	0.17	0	0
**Q1**	1.5	0.42	0	0	0
**Q3**	1.75	0.83	0.34	0	0.08
**Maximum**	2.83	1.33	0.67	0.67	0.33
**Minimum**	1.17	0	0	0	0
**n**	15	15	11	11	15
**Reduction vs. T0 (median)**		59.88%	89.82%	100.00%	100.00%

CI: Confidence Interval. SD: Standard Deviation. Q1: First quartile. Q3: Third quartile. RSS: Rhinoconjunctivitis Symptom Score.

Table S40. Descriptive statistics of the RSS in the subset of pediatric patients who completed five years of AIT.

	RSS					
	T0	T1	T2	T3	T4	T5
**Mean**	1.5	0.5	0.28	0	0	0
**95% lower CI**	1.08	-0.59	-0.19	0	0	0
**95% upper CI**	1.92	1.59	0.75	0	0	0
**SD**	0.17	0.44	0.19	0	0	0
**Median**	1.5	0.67	0.17	0	0	0
**Q1**	1.42	0.34	0.17	0	0	0
**Q3**	1.58	0.75	0.34	0	0	0
**Maximum**	1.67	0.83	0.5	0	0	0
**Minimum**	1.33	0	0.17	0	0	0
**n**	3	3	3	3	3	3
**Reduction vs. T0 (median)**		55.33%	88.67%	100.00%	100.00%	100.00%

CI: Confidence Interval. SD: Standard Deviation. Q1: First quartile. Q3: Third quartile. RSS: Rhinoconjunctivitis Symptom Score.

Table S41. Descriptive statistics of the ASS at baseline (T0) and after one year of allergen immunotherapy (T1) in pediatric patients.

	ASS	
	T0	T1
**Mean**	1.25	0.53
**95% lower CI**	1.11	0.36
**95% upper CI**	1.38	0.7
**SD**	0.48	0.61
**Median**	1.25	0.5
**Q1**	1	0
**Q3**	1.5	0.75
**Maximum**	2.25	3
**Minimum**	0	0
**n**	50	50
**Reduction vs. T0 (median)**		60.00%

CI: Confidence Interval. SD: Standard Deviation. Q1: First quartile. Q3: Third quartile. ASS: Asthma Symptom Score.

Table S42. Descriptive statistics of the ASS in the subset of pediatric patients who completed two years of AIT.

	ASS		
	T0	T1	T2
**Mean**	1.34	0.41	0.2
**95% lower CI**	1.14	0.25	0.07
**95% upper CI**	1.54	0.57	0.32
**SD**	0.46	0.37	0.28
**Median**	1.25	0.5	0
**Q1**	1	0	0
**Q3**	1.75	0.62	0.25
**Maximum**	2	1	1
**Minimum**	0.75	0	0
**n**	23	23	23
**Reduction vs. T0 (median)**		60.00%	100.00%

CI: Confidence Interval. SD: Standard Deviation. Q1: First quartile. Q3: Third quartile. ASS: Asthma Symptom Score.

Table S43. Descriptive statistics of the ASS in the subset of pediatric patients who completed three years of AIT.

	ASS			
	T0	T1	T2	T3
**Mean**	1.25	0.46	0.12	0.04
**95% lower CI**	0.95	0.08	-0.03	-0.02
**95% upper CI**	1.55	0.83	0.28	0.1
**SD**	0.46	0.59	0.21	0.1
**Median**	1	0.38	0	0
**Q1**	1	0	0	0
**Q3**	1.44	0.56	0.19	0
**Maximum**	2	2	0.5	0.25
**Minimum**	0.75	0	0	0
**n**	12	12	10	12
**Reduction vs. T0 (median)**		62.50%	100.00%	100.00%

CI: Confidence Interval. SD: Standard Deviation. Q1: First quartile. Q3: Third quartile. ASS: Asthma Symptom Score.

Table S44. Descriptive statistics of the ASS in the subset of pediatric patients who completed four years of AIT.

	ASS				
	T0	T1	T2	T3	T4
**Mean**	1.17	0.29	0.14	0.06	0.04
**95% lower CI**	0.91	0.1	-0.03	-0.03	-0.02
**95% upper CI**	1.42	0.48	0.31	0.14	0.1
**SD**	0.4	0.3	0.22	0.11	0.1
**Median**	1	0.25	0	0	0
**Q1**	1	0	0	0	0
**Q3**	1.06	0.5	0.25	0	0
**Maximum**	2	0.75	0.5	0.25	0.25
**Minimum**	0.75	0	0	0	0
**n**	12	12	9	9	12
**Reduction vs. T0 (median)**		75.00%	100.00%	100.00%	100.00%

CI: Confidence Interval. SD: Standard Deviation. Q1: First quartile. Q3: Third quartile. ASS: Asthma Symptom Score.

Table S45. Descriptive statistics of the ASS in the subset of pediatric patients who completed five years of AIT.

	ASS					
	T0	T1	T2	T3	T4	T5
**Mean**	1.5	0.38	0.12	0	0	0
**95% lower CI**	-4.85	-4.39	-1.46	0	0	0
**95% upper CI**	7.85	5.14	1.71	0	0	0
**SD**	0.71	0.53	0.18	0	0	0
**Median**	1.5	0.38	0.12	0	0	0
**Q1**	1.25	0.19	0.06	0	0	0
**Q3**	1.75	0.56	0.19	0	0	0
**Maximum**	2	0.75	0.25	0	0	0
**Minimum**	1	0	0	0	0	0
**n**	2	2	2	2	2	2
**Reduction vs. T0 (median)**		75.00%	91.67%	100.00%	100.00%	100.00%

CI: Confidence Interval. SD: Standard Deviation. Q1: First quartile. Q3: Third quartile. ASS: Asthma Symptom Score.
